# Synthesis of σ Receptor Ligands with a Spirocyclic System Connected with a Tetrahydroisoquinoline Moiety via Different Linkers

**DOI:** 10.1002/cmdc.202000861

**Published:** 2021-02-02

**Authors:** Melanie Bergkemper, Dirk Schepmann, Bernhard Wünsch

**Affiliations:** ^1^ Institut für Pharmazeutische und Medizinische Chemie Westfälische Wilhelms-Universität Münster Corrensstr. 48 48149 Münster Germany

**Keywords:** acyl linkers, alkyl linkers, sigma receptor affinity, spirocyclic sigma ligands, structure-affinity relationships, tetrahydroisoquinoline

## Abstract

With the aim to develop new σ_2_ receptor ligands, spirocyclic piperidines or cyclohexanamines with 2‐benzopyran and 2‐benzofuran scaffolds were connected to the 6,7‐dimethoxy‐1,2,3,4‐tetrahydroisoquinoline moiety by variable linkers. In addition to flexible alkyl chains, linkers containing an amide as functional group were synthesized. The 2‐benzopyran and 2‐benzofuran scaffold of the spirocyclic compounds were synthesized from 2‐bromobenzaldehyde. The amide linkers were constructed by acylation of amines with chloroacetyl chloride and subsequent nucleophilic substitution, the alkyl linkers were obtained by LiAlH_4_ reduction of the corresponding amides. For the development of σ_2_ receptor ligands, the spirocyclic 2‐benzopyran scaffold is more favorable than the ring‐contracted 2‐benzofuran system. Compounds bearing an alkyl chain as linker generally show higher σ affinity than acyl linkers containing an amide as functional group. A higher σ_1_ affinity for the *cis*‐configured cyclohexanamines than for the *trans*‐configured derivatives was found. The highest σ_2_ affinity was observed for *cis*‐configured spiro[[2]benzopyran‐1,1′‐cyclohexan]‐4′‐amine connected to the tetrahydroisoquinoline system by an ethylene spacer (*cis*‐**31**, *K*_i_ (σ_2_)=200 nM; the highest σ_1_ affinity was recorded for the corresponding 2‐benzofuran derivative with a CH_2_C=O linker (*cis*‐**29**, *K*_i_ (σ_1_)=129 nM).

## Graphical abstract

**No O for affinity**: Numerous σ_2_ receptor ligands inhibit tumor cell growth. In this project, two σ_2_ pharmacophoric elements were connected via various linkers to achieve high‐affinity σ_2_ ligands. Structure‐affinity relationship studies revealed that compounds with an ethylene linker exhibit higher σ_2_ affinity than compounds containing a carbonyl moiety within the linker.

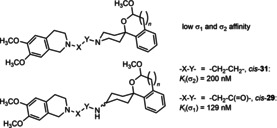

## 1 Introduction

σ Receptors, initially classified as class of opioid receptors, are well established as unique class of receptors without any homology to opioid receptors or NMDA receptors.^[1]^ Based on the results of comprehensive radioligand binding studies and biochemical analysis, the class of σ receptors was further divided into two distinct subtypes, which were termed σ_1_ and σ_2_ receptor.^[2]^

**Figure 1:**
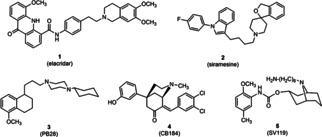
Elacridar and some prototypical σ_2_ receptor ligands.

The σ_1_ receptor has been cloned from different species, including human, rat, mouse, and guinea pig. The crystal structure of the human σ_1_ receptor was recently reported by Kruse et al.[^3^, ^4^] In contrast to the σ_1_ receptor, details concerning the σ_2_ receptor have been rather vague for many years. As a result from photoaffinity labeling studies a molecular weight of 21.5 kDa was postulated for the σ_2_ receptor.^[5]^ Xu and co‐workers utilized a photoaffinity probe to label σ_2_ receptors in rat liver and proposed that the σ_2_ receptor binding site resides within the progesterone receptor membrane component 1 (PGRMC1) complex.^[6]^ During the following years, the correlation between the σ_2_ receptor and PGRMC1 protein complex was considered controversial.^[7]^ In 2017, the σ_2_ receptor was isolated from calf liver tissue and identified as the endoplasmic reticulum (ER)‐resident membrane protein TMEM97, which is also described as MAC30 (meningioma‐associated protein 30). Subsequent molecular cloning and binding experiments confirmed this result. Mutagenesis studies identified two aspartate residues as crucial for binding of [^3^H]DTG, a radioligand frequently used in σ_2_ receptor binding assays. Furthermore, it was demonstrated that the TMEM97 ligands elacridar (**1**; Figure 1) and Ro 48‐8071 showed the same *K*_i_ values towards cell membranes from Sf9 cells overexpressing the TMEM97 protein and σ_2_ receptor overexpressing MCF‐7 cells.^[8]^ According to these findings, the σ_2_ receptor is now often termed σ_2_ receptor/TMEM97. In 2018, Riad et al. demonstrated that the σ_2_ receptor/TMEM97 protein, the PGRMC1 protein and the LDL receptor form a ternary complex, which is necessary for the rapid internalization of LDL.^[9]^ In contrast to the σ_1_ receptor, no crystal structure of the σ_2_ receptor protein has been published so far.

For a variety of tumor cells an overexpression of σ_2_ receptors was demonstrated, including breast cancer, lung cancer, colon cancer, leukemia and prostate cancer.[^10^, ^11^, ^12^, ^13^, ^14^, ^15^] It was also shown that σ_2_ receptor agonists are capable of killing tumor cells via apoptotic and non‐apoptotic mechanisms. For example, several derivatives of the high affinity σ_2_ receptor agonist PB28 (**3**; Figure 1) are able to inhibit the growth of pancreatic cancer cells and the neuroblastoma SK−N‐SH cell line.[^16^, ^17^] Very recently, it has been found that the potent and selective σ_2_ receptor ligand PB221 inhibits the proliferation of brain tumor murine astrocytoma cells (ALTS1C1).^[18]^ Haloperidol and its homopiperazine analog SYA013 exhibit high σ_2_ affinity and, furthermore, antiproliferative effects on different tumor cell lines, including Panc‐1.^[19]^ Therefore, the development of σ_2_ receptor ligands is a very promising goal. However, very recently it was reported that σ_2_ receptor ligands could also induce cytotoxic effects in σ_2_/TMEM97 knock out and σ_2_/TMEM97 and PGRMC1 double knock out cell lines. It was concluded that the cytotoxic effects of these σ_2_ ligands could not be mediated by the σ_2_ receptor, but other mechanisms have to be responsible for these cytotoxic effects.^[20]^

In Figure 1, some prototypical σ_2_ receptor ligands are shown. The spirocyclic benzofuran siramesine (**2**) displays a considerable selectivity for the σ_2_ receptor (*K*_i_=0.12 nM) over the σ_1_ receptor (*K*_i_=17 nM).^[21]^ PB28 (**3**) with the 4‐(cyclohexyl)piperazine substructure is also a potent σ_2_ ligand (*K*_i_=0.68 nM), but exhibits even higher affinity towards the σ_1_ subtype (*K*_i_=0.38 nM).^[22]^ In the group of bicyclic compounds some morphans (e. g., CB184, **4**) and granatanes (e. g., SV119, **5**) display high σ_2_ receptor affinity and high selectivity over the σ_1_ receptor.[^23^, ^24^]

**Figure 2:**
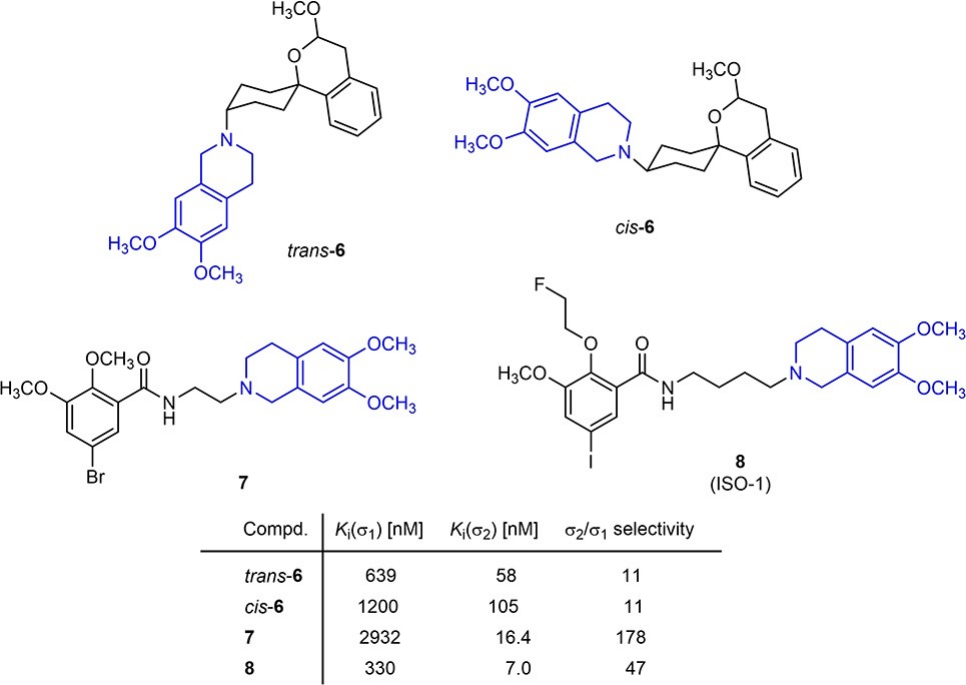
Lead compounds with a tetrahydroisoquinoline ring system showing a preference for σ_2_ receptors over σ_1_ receptors.

The 6,7‐dimethoxy‐1,2,3,4‐tetrahydroisoquinoline residue is a pharmacophoric element present in several σ_2_ receptor ligands.[^25^, ^26^, ^27^, ^28^, ^29^, ^30^, ^31^, ^32^, ^33^] (Figure 2) Mach and co‐workers published a series of benzamides connected to the 6,7‐dimethoxy‐1,2,3,4‐tetrahydroisoquinoline residue by linkers of different chain lengths. Among this series, **7** and **8** (ISO‐1^[32]^) with an ethylene and tetramethylene linker, respectively, showed high σ_2_ affinity and selectivity over the σ_1_ receptor.^[33]^ The same isoquinoline ring system is also a structural element of the σ_2_ ligand **1** (Figure 1).

**Figure 3:**
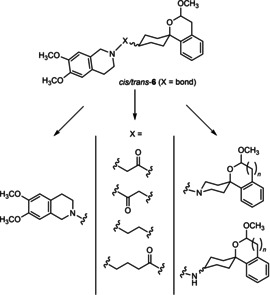
Overview of planned σ_2_ receptor ligands with various linkers.

In a previous study, we have reported that the spirocyclic 2‐benzopyran derivatives *trans*‐**6** and *cis*‐**6** bearing the 6,7‐dimethoxy‐1,2,3,4‐tetrahydroisoquinoline residue without linker show medium to high affinity to the σ_2_ receptor.^[34]^ (Figure 2) However, the selectivity over the σ_1_ subtype is moderate and has room for improvement. Therefore, it was envisaged to synthesize a new set of σ_2_ selective ligands by introducing a linker between the spirocyclic 2‐benzopyran scaffold and the isoquinoline ring system. To exploit further structure affinity relationships, not only alkyl chains were planned as linkers, but also amides with variable chain lengths and different positions of the carbonyl group were designed. Moreover, a ring contraction of the spirocyclic 2‐benzopyran to the spirocyclic 2‐benzofuran ring system was planned as this compound class is also known for its high σ affinity from previous studies. An overview of the structure modifications is presented in Figure 3.

## 2 Results and Discussion

### 2.1 Synthesis

**Scheme 1:**
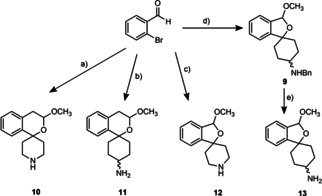
Outline of the synthesis of spirocyclic piperidines **10** ^[35]^ and **12**.^[36]^ and cyclohexanamines **11** ^[34]^ and **13** with 2‐benzopyran and 2‐benzofuran scaffold. a) 5 steps;^[35]^ b) 7 steps;^[34]^ c) 4 steps;^[36]^ d) 4 steps;[^37^, ^38^] e) NH_4_ HCO_2_, Pd/C, CH_3_OH, 17–21 h, 65 °C; *trans*‐**13**, 86 %, *cis*‐**13**, 66 %.

For the synthesis of the designed σ ligands, spirocyclic 2‐benzopyrans and 2‐benzofurans **10**–**13** with endocyclic and exocyclic amino moiety were synthesized starting from 2‐bromobenzaldehyde (Scheme 1).

The spirocyclic piperidines **10** and **12** were prepared by addition of an aryllithium intermediate at *N*‐benzyl‐protected piperidin‐4‐one as previously described.[^35^, ^36^] The exocyclic primary amines *trans*‐**11** and *cis*‐**11** were obtained as reported in ref. [34]. Transfer hydrogenolysis using NH_4_ HCO_2_ in the presence of Pd/C converted *trans*‐ and *cis*‐configured benzylamines *trans*‐**9** and *cis*‐**9**[^37^, ^38^] into the diastereomeric primary amines *trans*‐**13** and *cis*‐**13**. The secondary amine 6,7‐dimethoxy‐1,2,3,4‐tetrahydroisoquinoline HCl (**14** HCl) was commercially available (Scheme 1).

**Scheme 2:**
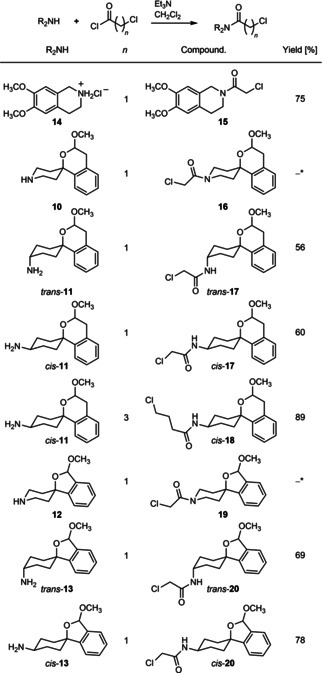
Acylation of amines with chloroacyl chlorides. *The spirocyclic piperidines **16** and **19** were not isolated, but directly used for subsequent nucleophilic substitution with **14**.

The amines **10**–**14** were acylated with α‐chloroacetyl chloride affording chloroacetamides **15** ^[39]^–**17** and **19**–**20**. The homologous 4‐chlorobutyryl derivative *cis*‐**18** was prepared by reaction of *cis*‐**11** with 4‐chlorobutyryl chloride. The amides **15**–**20** were obtained in yields of 56–89 % (Scheme 2). Acylation of tetrahydroisoquinoline **14** with 3‐chloropropionyl chloride did not lead to the desired 3‐chloropropinamide.

The final compounds were obtained by nucleophilic substitution of the terminal chloride in the side chain of the amides **15**–**20**. The acylated spirocyclic 2‐benzopyrans **16**–**18** and 2‐benzofurans **19** and **20** were reacted with the tetrahydroisoquinoline **14**, while the acylated isoquinoline **15** underwent a nucleophilic substitution with the spirocyclic amines **10**–**13**. In Table 1, the products and yields of these transformations are summarized.

**Table 1:** Nucleophilic substitution at chloroamides **15**–**20**.^[a]^

Chloroamide	Amine	Product	Yield [%]
**15**	**10**	**21**	22
**15**	*trans‐* **11**	*trans*‐**22**	–*
**15**	*cis‐* **11**	*cis*‐**22**	60
**15**	**12**	**23**	36
**15**	*trans‐* **13**	*trans*‐**24**	54
**15**	*cis‐* **13**	*cis*‐**24**	43
**16**	**14**	**25**	75
*trans*‐**17**	**14**	*trans*‐**26**	83
*cis*‐**17**	**14**	*cis*‐**26**	62
*cis*‐**18**	**14**	*cis*‐**27**	19
**19**	**14**	**28**	44
*trans*‐**20**	**14**	*trans*‐**29**	66
*cis*‐**20**	**14**	*cis*‐**29**	86

[a] For structures, see Scheme 2 and Table 2. *The product could not be isolated.

The nucleophilic substitution of the 2‐chloroacetylated isoquinoline derivative **15** with spirocyclic amines **10**–**13** in DMF with TBAI as catalyst resulted in the formation of the desired compounds **21**–**24** in satisfactory yields. Due to purification problems, the benzopyran‐based spirocyclic compound *trans*‐**22** could not be isolated in pure form for testing. S_N_2 reaction of spirocyclic chloroacetamides **16**, **17** and **20** with the tetrahydroisoquinoline **14** provided the amides **25**, **26** and **29** in 62–86 % yields. The pure spirocyclic benzofuran **28** was obtained in only 44 % yield, due to purification problems. While the nucleophilic substitution of the 2‐chloroacetylated compounds **16**, **17**, **19**, and **20** with tetrahydroisoquinoline **14** led to clean conversions, he corresponding 4‐chlorobutyramide **18** reacted slower to produce *cis*‐**27**, which was isolated in only 19 % yield (Table 1).

During the reaction to obtain the secondary amines *trans*‐**22**, *cis*‐**22**, *trans*‐**24** and *cis*‐**24**, formation of tertiary amines as side‐products was observed (double nucleophilic substitution). The *R*_f_ values of the tertiary amines was almost identical to the *R*_f_ value of the secondary amines, rendering the fc purification of the desired products very difficult. Although the isolation and purification of the secondary amines *cis*‐**22**, *trans‐* **24** and *cis*‐**24** was successful, *trans*‐**22** could not be isolated in sufficient purity.

**Scheme 3:**
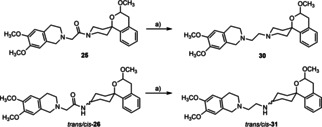
Synthesis of isoquinolines **30**, *trans*‐**31** und *cis*‐**31** with an ethylene linker. a) LiAlH_4_, THF, 2–22 h, 70 °C, 63 % (**30**), 86 % (*trans*‐**31**), 76 % (*cis*‐**31**).

As not only linkers bearing a carbonyl group were planned, derivatives **30**, *trans*‐**31** and *cis*‐**31** with an ethylene linker between the amino moiety of the spirocyclic benzopyran and the tetrahydroisoquinoline were synthesized. (Scheme 3) This type of compounds features two basic amino moieties instead of one and can therefore adopt different orientations within the binding pocket of both σ receptor subtypes. Additionally, the effect of the carbonyl moiety on the binding affinity and selectivity can be studied.

At first, a direct alkylation of the tetrahydroisoquinoline **14** was envisaged. For this purpose, 2‐bromoethanol was oxidized with Dess‐Martin perioidinane to afford 2‐bromoacetaldehyde. The aldehyde should be attached to the isoquinoline **14** in a reductive alkylation with NaBH(OAc)_3_. Unfortunately, after 4 h reaction time the alkylated isoquinoline could not be isolated. Next, a nucleophilic substitution with 1,2‐dibromoethane and K_2_CO_3_ in CH_3_CN was performed. But even after a reaction time of 18 h the desired product could not be obtained. The reaction conditions which led to a successful acylation of the isoquinoline **14** (DMF, Et_3_N and TBAI) also didn′t lead to the formation of the alkylated product. Finally, the desired alkylated amines **30**, *trans*‐**31** and *cis*‐**31** were synthesized by reduction of the corresponding amides **25**, *trans*‐**26** and *cis*‐**26** with LiAlH_4_. (Scheme 3) The piperidine derivative **30** was obtained in 63 % yield after 2 h heating to reflux. *trans*‐**31** and *cis*‐**31** were isolated after 22 h in 86 and 76 % yield, respectively.

### 2.2 σ_1_ and σ_2_ receptor affinity

Competitive binding assays with tritiated radioligands were utilized to determine the σ_1_ and σ_2_ receptor affinity of the synthesized compounds. In the σ_1_ binding assay, [^3^H]‐(+)‐pentazocine was used as radioligand and homogenates of guinea pig brains served as receptor material. The σ_2_ assay was performed with the radioligand [^3^H]‐di(o‐tolyl)guanidine ([^3^H]DTG) and homogenates of rat liver were used as receptor material. The nonselective properties of DTG was compensated by masking σ_1_ receptors with an excess of non‐tritiated (+)‐pentazocine.^[40]^

In Table 2, the receptor affinities of the synthesized compounds are summarized. In comparison to the lead compounds *trans*‐**7** and *cis*‐**7**, the 2‐benzopyran derivatives with an acetyl linker generally show a lower σ_2_ affinity. The highest σ_2_ affinity was observed for *cis*‐**26** with a *K*_i_ value of 371 nM. In this compound, the acyl group is located at the spirocyclic ring system. When the acyl moiety is located at the isoquinoline ring system (*cis*‐**22**), the σ_2_ affinity is reduced (11 % inhibition of radioligand binding). A similar trend was observed for the corresponding piperidine derivatives **25** and **21** with *K*_i_ values of 534 nM and 19 % inhibition of radioligand binding, respectively.

**Table 2:** σ1 and σ2 receptor affinities of synthesized compounds.

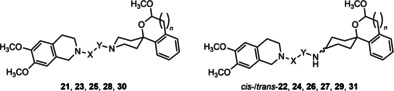					
Compound	X	Y	n	*K*_i_ [nM]±SEM^[a]^	
				σ_1_	σ_2_
trans‐**6**	–	–	1	639	58±27
cis‐**6**	–	–	1	1200	105±8
**21**	C=O	CH_2_	1	319	19 %
cis‐**22**	C=O	CH_2_	1	740	11 %
**23**	C=O	CH_2_	0	0 %	0 %
trans‐**24**	C=O	CH_2_	0	15 %	0 %
cis‐**24**	C=O	CH_2_	0	1600	9 %
**25**	CH_2_	C=O	1	518	534
trans‐**26**	CH_2_	C=O	1	7 %	4 %
cis‐**26**	CH_2_	C=O	1	1000	371
cis‐**27**	(CH_2_)_3_	C=O	1	712	2100
**28**	CH_2_	C=O	0	19 %	3400
trans‐**29**	CH_2_	C=O	0	1300	1900
cis‐**29**	CH_2_	C=O	0	129	0 %
**30**	CH_2_	CH_2_	1	608	348
trans‐**31**	CH_2_	CH_2_	1	1400	499
cis‐**31**	CH_2_	CH_2_	1	251	200

[a] *K*_i_ values are given as means of 3 different experiments; percentage values indicate inhibition of the radioligand at a concentration of 1 μM of the test compound.

The σ_1_ affinity of the piperidine derivative **21** is higher than that of the corresponding cyclohexanamine derivative *cis‐* **22**. A general observation is that the σ_1_ affinity is higher for the compounds bearing the acyl group at the isoquinoline ring. For the development of σ_1_ ligands with the 2‐benzoypran scaffold, it can therefore be concluded that the basic center at the spirocyclic ring system should be retained.

For the derivatives with the 2‐benzofuran scaffold similar observations were made in terms of σ_2_ affinity. The introduction of an acetyl spacer led to loss of σ_2_ affinity, independent of the position of the acyl moiety (e. g., *trans*‐**24**, *cis*‐**24**, **28**). In contrast to the 2‐benzopyrans, the σ_1_ affinity of the piperidine derivatives of the spirocyclic 2‐benzofurans was not higher than the respective cyclohexanamines. A notable exception is *cis*‐**29** with a *K*_i_ value of 129 nM at the σ_1_ receptor. This compound even represents a σ_1_ receptor selective ligand despite the 6,7‐dimethoxy‐1,2,3,4‐tetrahydroisoquinoline structural element.

The elongation of the acyl linker also led to a decrease in σ_2_ affinity, while the σ_1_ affinity was slightly increased. For the butyramide *cis*‐**27** a *K*_i_ value of 2100 nM at the σ_2_ receptor and 712 nM at the σ_1_ receptor was observed.

For the derivatives **30**, *cis*‐**31** and *trans*‐**31** with an ethylene linker an increased σ_2_ affinity in comparison to the corresponding amides (e. g., **21**, *cis*‐**22**) was found. The σ_1_ receptor affinity of the cyclohexanamines *cis*‐**31** and *trans*‐**31** was also increased, resulting in a loss of σ_2_ preference of *cis*‐**31**. The piperidine **30** shows a slight preference for the σ_2_ receptor (*K*_i_ values of 348 nM and 608 nM, respectively).

## 3 Conclusion

The introduction of a spacer between the spirocyclic 2‐benzopyran and 2‐benzofuran scaffold and the tetrahydroisoquinoline system was envisaged to study structure affinity relationships and evaluate possibilities to optimize selectivity of the lead compounds *trans*‐**7** and *cis*‐**7**. A set of compounds with amide and alkyl spacers was synthesized and pharmacologically evaluated in competitive binding assays. Although the introduction of the linker generally resulted in a loss of σ affinity in comparison to the lead compounds **7** without linker, some interesting observations could be made. Compounds containing the 2‐benzopyran scaffold showed a higher affinity than the corresponding 2‐benzofurans. Compounds **30**, *trans*‐**31** and *cis*‐**31** with an ethylene linker displayed higher affinity than compounds with an amide in the side chain. The introduction of the linker in compounds **21** and *cis*‐**29** resulted in an unexpected selectivity for the σ_1_ receptor. In conclusion, the combination of wo promising σ_2_ pharmacophoric elements, that is, the connection of an O‐containing spirocyclic system with the tetrahydroisoquinoline moiety by different spacers, did not provide high‐affinity σ_2_ selective ligands. However, the synthesized σ ligands allow an interesting insight into the limitations of acyl chains as linker between the two pharmacophoric elements. *cis*‐**31** and *trans*‐**31** could serve as a starting point for further structural modifications resulting in higher σ_2_ affinity and selectivity.

## Experimental Section

### Chemistry, General

Unless otherwise noted, moisture sensitive reactions were conducted under dry nitrogen. CH_2_Cl_2_ was distilled over CaH_2_. THF was distilled over sodium/benzophenone. Thin layer chromatography (tlc): Silica gel 60 F254 plates (Merck). Flash chromatography (fc): Silica gel 60, 40–64 μm (Merck); parentheses include: diameter of the column (*d*), length of the stationary phase (*l*), fraction size (*V*), eluent. Melting point: Melting point apparatus Mettler Toledo MP50 melting point system, uncorrected. MS: microTOF−Q II (Bruker Daltonics); APCI, atmospheric pressure chemical ionization; microTof mass spectrometer (Bruker Daltonics); ESI, electrospray ionization. IR: FTIR spectrophotometer MIRacle 10 (Shimadzu) equipped with ATR technique. Nuclear magnetic resonance (NMR) spectra were recorded on Agilent 600‐MR (600 MHz for ^1^H, 151 MHz for ^13^C) or Agilent 400‐MR spectrometer (400 MHz for ^1^H, 101 MHz for ^13^C); *δ* in ppm related to tetramethylsilane and measured referring to CHCl_3_ (*δ*=7.26 ppm (^1^H NMR) and *δ*=77.2 ppm (^13^C NMR)) and CHD_2_OD (*δ*=3.31 ppm (^1^H NMR) and *δ*=49.0 ppm (^13^C NMR)); coupling constants are given with 0.5 Hz resolution; the assignments of ^13^C and ^1^H NMR signals were supported by 2‐D NMR techniques where necessary (data not shown); multiplicities of the signals are abbreviated as follows: s=singlet, d=doublet, t=triplet, q=quartet; dd=doublet of doublets, m=multiplet. HPLC: pump: LPG‐3400SD, degasser: DG‐1210, autosampler: ACC‐3000T, UV‐detector: VWD‐3400RS, interface: DIONEX UltiMate 3000, data acquisition: Chromeleon 7 (Thermo Fisher Scientific); column: LiChrospher® 60 RP‐select B (5 μm), LiChroCART® 250–4 mm cartridge; guard column: LiChrospher® 60 RP‐select B (5 μm), LiChroCART® 4–4 mm cartridge (no.: 1.50963.0001), manu‐CART® NT cartridge holder; flow rate: 1.0 mL/min; injection volume: 5.0 μL; detection at *λ*=210 nm; solvents: A: water with 0.05 % (*v*/*v*) trifluoroacetic acid; B: acetonitrile with 0.05 % (*v*/*v*) trifluoroacetic acid: gradient elution: (A %): 0–4 min: 90 %, 4–29 min: 90→0 %, 29–31 min: 0 %, 31–31.5 min: 0→90 %, 31.5–40 min: 90 %. The purity of all compounds was determined by this method. Unless otherwise mentioned, the purity of all test compounds is higher than 95 %.

### Synthetic procedures

The synthesis of the spirocyclic piperidines **10** and **12** has been reported in the literature.[^35^, ^36^] The synthesis of exocyclic primary amines *trans*‐**11** and *cis*‐**11** was described in ref. [34]. The synthesis of *trans*‐ and *cis*‐configured benzylamines *trans*‐**9** and *cis*‐**9** was reported in ref. [37] and [38].

### *trans*‐3‐Methoxy‐3H‐spiro[[2]benzofuran‐1,1′‐cyclohexan]‐4′‐amine (*trans*‐13)


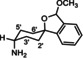


A solution of benzylamine *trans*‐**9** (248 mg, 0.76 mmol), ammonium formate (255 mg, 4.05 mmol, 5.3 equiv) and 10 % Pd/C (35 mg, 0.03 mmol, 4 mol‐%) in CH_3_OH (15 mL) was heated to reflux for 17 h. The mixture was filtered through Celite, washed with CH_2_Cl_2_ (150 mL) and concentrated in vacuo. 1 M NaOH (15 mL) was added and the aqueous layer was extracted with CH_2_Cl_2_ (3×15 mL). The combined organic layers were dried (Na_2_SO_4_), filtered and concentrated in vacuo. Yellow oil, yield 153 mg (86 %). C_14_H_19_NO_2_ (233.3 g/mol). TLC: *R*_f_=0.03 (cyclohexane/ethyl acetate 67 : 33+1 % *N*,*N*‐dimethylethanamine). HRMS (APCI, method 1): *m/z* 234.1477 (calcd. 234.1489 for C_14_H_20_NO_2_ [*M*H^+^]). ^1^H NMR (600 MHz, CD_3_OD): *δ*=1.58–1.64 (m, 1H, 2′‐*H*), 1.65–1.76 (m, 3H, 3′‐*H*, 5′‐*H*, 6′‐*H*), 2.01–2.09 (m, 4H, 2′‐*H*, 3′‐*H*, 5′‐*H*, 6′‐*H*), 3.11–3.15 (m, 1H, 4′‐*H*_equ_), 3.47 (s, 3H, OC*H*_3_), 6.04 (s, 1H, 3‐*H*), 7.34–7.38 (m, 2H, 4‐*H*, 5‐*H*), 7.38–7.42 (m, 1H, 6‐*H*), 7.49 ppm (d, *J*=7.7 Hz, 1H, 7‐*H*). A signal for the NH_2_ protons is not observed in the spectrum. ^13^C NMR (151 MHz, CD_3_OD): *δ*=30.5 (1 C, *C*‐3′ or *C*‐5′), 30.7 (1 C, *C*‐3′ or *C*‐5′), 34.0 (1 C, *C*‐2′), 35.2 (1 C, *C*‐6′), 47.2 (1 C, *C*‐4′), 54.8 (1 C, O*C*H_3_), 88.0 (1 C, *C*‐1), 106.9 (1 C, *C*‐3), 122.6 (1 C, *C*‐7), 124.2 (1 C, *C*‐4), 128.9 (1 C, *C*‐5), 130.3 (1 C, *C*‐6), 138.8 (1 C, *C*‐3a), 148.8 ppm (1 C, *C*‐7a). FTIR (neat): *ν* [cm^−1^]=3364 (N−H), 2924, 2855 (C−H_alkyl_), 1435, 1366 (C=C_arom_). Purity (HPLC): 93.9 %, *t*_R_=8.7 min.

### *cis*‐3‐Methoxy‐3*H*‐spiro[[2]benzofuran‐1,1′‐cyclohexan]‐4′‐amine (*cis*‐13)


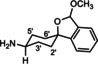


A solution of benzylamine *cis*‐**9** (414 mg, 1.28 mmol), ammonium formate (406 mg, 6.44 mmol, 5.0 equiv) and 10 % Pd/C (55 mg, 0.05 mmol, 4 mol‐%) in CH_3_OH (25 mL) was heated to reflux for 21 h. The mixture was filtered through Celite, washed with CH_2_Cl_2_ (200 mL) and concentrated in vacuo. 1 M NaOH (15 mL) was added and the aqueous phase was extracted with CH_2_Cl_2_ (3×15 mL). The combined organic layers were dried (Na_2_SO_4_), filtered and concentrated in vacuo. Yellow oil, yield 198 mg (66 %). C_14_H_19_NO_2_ (233.3 g/mol). TLC: *R*_f_=0.02 (cyclohexane/ethyl acetate 67 : 33+1 % *N*,*N*‐dimethylethanamine). HRMS (APCI, method 1): *m/z* 234.1479 (calcd. 234.1489 for C_14_H_20_NO_2_ [*M*H^+^]). ^1^H NMR (600 MHz, CD_3_OD): *δ*=1.63–1.68 (m, 1H, 2′‐*H*), 1.68–1.76 (m, 2H, 3′‐*H*, 5′‐*H*), 1.82–1.89 (m, 4H, 3′‐*H*, 5′‐*H*, 6′‐*H*), 1.93 (td, *J*=13.5/4.1 Hz, 1H, 2′‐*H*), 2.82 (tt, *J*=11.5/3.8 Hz, 1H, 4′‐*H*_ax_), 3.49 (s, 3H, OC*H*_3_), 6.05 (s, 1H, 3‐*H*), 7.23 (d, *J*=7.4 Hz, 1H, 7‐*H*), 7.32–7.41 ppm (m, 3H, 4‐*H*, 5‐*H*, 6‐*H*). A signal for the NH_2_ protons is not observed in the spectrum. ^13^C NMR (151 MHz, CD_3_OD): *δ*=32.5 (1 C, *C*‐3′ or *C*‐5′), 32.9 (1 C, *C*‐3′ or *C*‐5′), 37.6 (1 C, *C*‐2′), 38.9 (1 C, *C*‐6′), 50.5 (1 C, *C*‐4′), 54.9 (1 C, O*C*H_3_), 87.1 (1 C, *C*‐1), 107.1 (1 C, *C*‐3), 121.7 (1 C, *C*‐7), 124.2 (1 C, *C*‐4), 128.9 (1 C, *C*‐5), 130.4 (1 C, *C*‐6), 138.7 (1 C, *C*‐3a), 148.6 ppm (1 C, *C*‐7a). FTIR (neat): *ν* [cm^−1^]=3356 (N−H), 2928, 2859 (C−H_alkyl_), 1458, 1439 (C=C_arom_). Purity (HPLC): 99.3 %, *t*_R_=10.5 min.

### 2‐Chloro‐1‐(6,7‐dimethoxy‐3,4‐dihydroisoquinolin‐2(1*H*)‐yl)ethan‐1‐one (15)


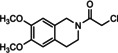


The compound was synthesized according to the literature.^[39]^ 2‐Chloroacetyl chloride (0.12 mL, 1.51 mmol, 1.2 equiv) was slowly added to a solution of isoquinoline **14**⋅HCl (281 mg, 1.22 mmol) and Et_3_N (0.42 mL, 3.03 mmol, 2.5 equiv) in CH_2_Cl_2_ (30 mL) under N_2_ at 0 °C. After stirring for 4.5 h at RT, H_2_O (50 mL) was added and the aqueous layer was extracted with CH_2_Cl_2_ (3×50 mL). The combined organic layers were dried (Na_2_SO_4_), filtered, concentrated in vacuo and the residue was purified by fc (*d*=2.5 cm, *l*=20 cm, *V*=10 mL, cyclohexane/ethyl acetate 67 : 33). Pale yellow solid, m.p. 109 °C, yield 247 mg (75 %). C_13_H_16_ClNO_3_ (269.7 g/mol). *R*_f_=0.26 (cyclohexane/ethyl acetate 50 : 50). HRMS (APCI): *m/z* 270.0864 (calcd. 270.0891 for C_13_H_17_^35^ClNO_3_ [*M*H^+^]). ^1^H NMR (400 MHz, CDCl_3_): *δ*=2.80 (t, *J*=6.0 Hz, 0.8H, 4‐*H*), 2.89 (t, *J*=5.9 Hz, 1.2H, 4‐*H*), 3.73 (t, *J*=5.9 Hz, 1.2H, 3‐*H*), 3.81–3.88 (m, 6.8H, 3‐*H*, 6‐OC*H*_3_, 7‐OC*H*_3_), 4.15 (s, 1.2H, COC*H*_2_Cl), 4.16 (s, 0.8H, COC*H*_2_Cl), 4.62 (s, 0.8H, 1‐*H*), 4.67 (s, 1.2H, 1‐*H*), 6.59–6.65 ppm (m, 2H, 5‐*H*, 8‐*H*). ^13^C NMR (101 MHz, CDCl_3_): *δ*=27.9 (0.4 C, *C*‐4), 29.1 (0.6 C, *C*‐4), 40.7 (0.4 C, *C*‐3), 41.3 (0.6 C, CO*C*H_2_Cl), 41.6 (0.4 C, CO*C*H_2_Cl), 44.1 (0.6 C, *C*‐3), 44.6 (0.6 C, *C*‐1), 47.7 (0.4 C, *C*‐1), 56.13 (1.2 C, 6‐O*C*H_3_, 7‐O*C*H_3_), 56.18 (0.8 C, 6‐O*C*H_3_, 7‐O*C*H_3_), 109.0 (0.4 C, *C*‐8), 109.5 (0.6 C, *C*‐8), 111.4 (0.6 C, *C*‐5), 111.7 (0.4 C, *C*‐5), 123.7 (0.4 C, *C*‐8a), 124.6 (0.6 C, *C*‐8a), 125.6 (0.6 C, *C*‐4a), 126.7 (0.4 C, *C*‐4a), 148.0 (1.2 C, *C*‐6, *C*‐7), 148.2 (0.8 C, *C*‐6, *C*‐7), 165.5 (0.4 C, *C*=O), 165.6 ppm (0.4 C, *C*=O). FTIR (neat): *ν* [cm^−1^]=2974, 2932 (C−H_alkyl_), 1651 (C=O), 1520, 1443 (C=C_arom_). Purity (HPLC): 99.7 %, *t*_R_=15.9 min.

### *trans*‐2‐Chloro‐*N*‐(3‐methoxy‐3,4‐dihydrospiro[[2]benzopyran‐1,1′‐cyclohexan]‐4′‐yl)acetamide (*trans*‐17)


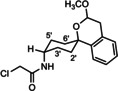


2‐Chloroacetyl chloride (19 μL, 0.24 mmol, 1.2 equiv) was slowly added to a solution of amine *trans*‐**11** (50 mg, 0.20 mmol) and Et_3_N (0.07 mL, 0.50 mmol, 2.5 equiv) in CH_2_Cl_2_ (5 mL) under N_2_ at 0 °C. After stirring for 6.5 h at RT, H_2_O (30 mL) was added and the aqueous layer was extracted with CH_2_Cl_2_ (3×30 mL). The combined organic layers were dried (Na_2_SO_4_), filtered, concentrated in vacuo and the residue was purified by fc (*d*=2 cm, *l*=16 cm, *V*=5 mL, cyclohexane/ethyl acetate 50 : 50). Pale yellow solid, m.p. 144 °C, yield 36 mg (56 %). C_17_H_22_ClNO_3_ (323.8 g/mol). *R*_f_=0.22 (cyclohexane/ethyl acetate 67 : 33). HRMS (APCI): *m/z* 292.1080 (calcd. 292.1099 for C_16_H_19_^35^ClNO_2_ [*M*‐CH_3_OH+H^+^]). ^1^H NMR (600 MHz, CD_3_OD): *δ*=1.68–1.72 (m, 1H, 2′‐*H*), 1.76–1.81 (m, 2H, 3′‐*H*, 5′‐*H*), 1.88 (td, *J*=14.1/3.8 Hz, 1H, 6′‐*H*), 1.92–1.97 (m, 1H, 6′‐*H*), 2.10–2.25 (m, 3H, 2′‐*H*, 3′‐*H*, 5′‐*H*), 2.81 (dd, *J*=15.6/7.4 Hz, 1H, 4‐*H*), 2.94 (dd, *J*=15.6/3.1 Hz, 1H, 4‐*H*), 3.55 (s, 3H, OC*H*_3_), 4.12–4.16 (m, 1H, 4′‐*H*_equ_), 4.13 (s, 2H, COC*H*_2_Cl), 4.92 (dd, *J*=7.4/3.1 Hz, 1H, 3‐*H*), 7.10 (dd, *J*=7.6/1.3 Hz, 1H, 5‐*H*), 7.17 (td, *J*=7.4/1.3 Hz, 1H, 6‐*H*), 7.20–7.23 (m, 1H, 7‐*H*), 7.29 ppm (dd, *J*=7.8/1.3 Hz, 1H, 8‐*H*). A signal for the NH proton is not observed in the spectrum. ^13^C NMR (151 MHz, CD_3_OD): *δ*=26.2 (1 C, *C*‐3′), 26.3 (1 C, *C*‐5′), 32.0 (1 C, *C*‐6′), 34.4 (1 C, *C*‐2′), 36.1 (1 C, *C*‐4), 43.4 (1 C, CO*C*H_2_Cl), 45.8 (1 C, *C*‐4′), 56.4 (1 C, O*C*H_3_), 77.4 (1 C, *C*‐1), 97.9 (1 C, *C*‐3), 125.7 (1 C, *C*‐8), 127.5 (1 C, *C*‐7), 127.7 (1 C, *C*‐6), 130.1 (1 C, *C*‐5), 132.5 (1 C, *C*‐4a), 143.0 (1 C, *C*‐8a), 169.2 ppm (1 C, *C*=O). FTIR (neat): *ν* [cm^−1^]=3321 (N−H), 2928, 2851 (C−H_alkyl_), 1636 (C=O), 1547, 1447 (C=C_arom_). Purity (HPLC): 94.8 %, *t*_R_=18.9 min.

### *cis*‐2‐Chloro‐*N*‐(3‐methoxy‐3,4‐dihydrospiro[[2]benzopyran‐1,1′‐cyclohexan]‐4′‐yl)acetamide (*cis*‐17)


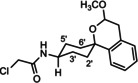


2‐Chloroacetyl chloride (19 μL, 0.24 mmol, 1.2 equiv) was slowly added to a solution of amine *cis*‐**11** (50 mg, 0.20 mmol) and Et_3_N (0.07 mL, 0.50 mmol, 2.5 equiv) in CH_2_Cl_2_ (5 mL) under N_2_ at 0 °C. After stirring for 6 h at RT, H_2_O (30 mL) was added and the aqueous layer was extracted with CH_2_Cl_2_ (3×30 mL). The combined organic layers were dried (Na_2_SO_4_), filtered, concentrated in vacuo and the residue was purified by fc (*d*=2 cm, *l*=20 cm, *V*=10 mL, cyclohexane/ethyl acetate 67 : 33). Colorless solid, m.p. 148 °C, yield 40 mg (60 %). C_17_H_22_ClNO_3_ (323.8 g/mol). *R*_f_=0.44 (cyclohexane/ethyl acetate 50 : 50). HRMS (APCI): *m/z* 292.1072 (calcd. 292.1099 for C_16_H_19_^35^ClNO_2_ [*M*‐CH_3_OH+H^+^]). ^1^H NMR (600 MHz, CD_3_OD): *δ*=1.77–1.91 (m, 5H, 2′‐*H*_ax_, 3′‐*H*, 5′‐*H*, 6′‐*H*_equ_), 1.92–1.97 (m, 1H, 5′‐*H*), 2.08 (td, *J*=13.2/3.9 Hz, 1H, 6′‐*H*_ax_), 2.13 (dq, *J*=14.0/2.9 Hz, 1H, 2′‐*H*_equ_), 2.81 (dd, *J*=15.6/7.5 Hz, 1H, 4‐*H*), 2.94 (dd, *J*=15.8/2.9 Hz, 1H, 4‐*H*), 3.58 (s, 3H, OC*H*_3_), 3.88 (tt, *J*=11.3/4.7 Hz, 1H, 4′‐*H*_ax_), 4.03 (s, 2H, COC*H*_2_Cl), 4.92 (dd, *J*=7.5/3.1 Hz, 1H, 3‐*H*), 7.09 (d, *J*=7.3 Hz, 1H, 5‐*H*), 7.16 (td, *J*=7.2/1.9 Hz, 1H, 6‐*H*), 7.18–7.24 ppm (m, 2H, 7‐*H*, 8‐*H*). A signal for the NH proton is not observed in the spectrum. ^13^C NMR (151 MHz, CD_3_OD): *δ*=28.7 (1 C, *C*‐5′), 28.8 (1 C, *C*‐3′), 36.1 (1 C, *C*‐4), 36.5 (1 C, *C*‐2′), 39.1 (1 C, *C*‐6′), 43.3 (1 C, CO*C*H_2_Cl), 49.7 (1 C, *C*‐4′), 56.4 (1 C, O*C*H_3_), 76.9 (1 C, *C*‐1), 97.9 (1 C, *C*‐3), 125.7 (1 C, *C*‐8), 127.6 (1 C, *C*‐7), 127.7 (1 C, *C*‐6), 130.1 (1 C, *C*‐5), 132.6 (1 C, *C*‐4a), 142.5 (1 C, *C*‐8a), 168.6 ppm (1 C, *C*=O). FTIR (neat): *ν* [cm^−1^]=3302 (N−H), 2932, 2862 (C−H_alkyl_), 1643 (C=O), 1447 (C=C_arom_). Purity (HPLC): 97.1 %, *t*_R_=18.6 min.

### *cis*‐4‐Chloro‐*N*‐(3‐methoxy‐3,4‐dihydrospiro[[2]benzopyran‐1,1′‐cyclohexan]‐4′‐yl)butanamide (*cis*‐18)


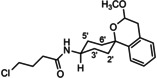


4‐Chlorobutyryl chloride (27.4 μL, 0.24 mmol, 1.1 equiv) was slowly added to a solution of amine *cis*.**11** (54 mg, 0.22 mmol) and Et_3_N (0.07 mL, 0.50 mmol, 2.3 equiv.) in CH_2_Cl_2_ (15 mL) under N_2_ at 0 °C. After stirring for 4 h at RT, H_2_O (30 mL) was added and the aqueous layer was extracted with CH_2_Cl_2_ (3×30 mL). The combined organic layers were dried (Na_2_SO_4_), filtered and concentrated in vacuo. Colorless solid, m.p. 126 °C, yield 69 mg (89 %). C_19_H_26_ClNO_3_ (351.9 g/mol). *R*_f_=0.37 (cyclohexane/ethyl acetate 50 : 50). HRMS (ESI): *m/z* 374.1504 (calcd. 374.1493 for C_19_H_26_^35^ClNO_3_Na [*M*Na^+^]). ^1^H NMR (400 MHz, CD_3_OD): *δ*=1.78–1.94 (m, 6H, 2′‐*H*, 3′‐*H*, 5′‐*H*, 6′‐*H*), 2.02–2.17 (m, 4H, 2′‐H, 6′‐H, COCH_2_C*H*_2_CH_2_Cl), 2.39 (t, *J*=7.3 Hz, 2H, COC*H*_2_CH_2_CH_2_Cl), 2.82 (dd, *J*=15.7/7.5 Hz, 1H, 4‐*H*), 2.94 (dd, *J*=15.7/3.2 Hz, 1H, 4‐*H*), 3.60 (s, 3H, OC*H*_3_), 3.63 (t, *J*=6.5 Hz, 2H, COCH_2_CH_2_C*H*_2_Cl), 3.81–3.91 (m, 1H, 4′‐*H*_ax_), 4.93 (dd, *J*=7.4/3.2 Hz, 1H, 3‐*H*), 7.10 (d, *J*=7.0 Hz, 1H, 5‐*H*), 7.14–7.26 ppm (m, 3H, 6‐*H*, 7‐*H*, 8‐*H*). A signal for the NH proton is not observed in the spectrum. ^13^C NMR (151 MHz, CD_3_OD): *δ*=28.9 (1 C, *C*‐3′ or *C*‐5′), 29.1 (1 C, *C*‐3′ or *C*‐5′), 30.0 (1 C, COCH_2_ *C*H_2_CH_2_Cl), 34.2 (1 C, CO*C*H_2_CH_2_CH_2_Cl), 36.1 (1 C, *C*‐4), 36.5 (1 C, *C*‐2′), 39.1 (1 C, *C*‐6′), 45.1 (1 C, COCH_2_CH_2_ *C*H_2_Cl), 49.1 (1 C, *C*‐4′), 56.4 (1 C, O*C*H_3_), 77.0 (1 C, *C*‐1), 97.8 (1 C, *C*‐3), 125.7 (1 C, *C*‐8), 127.5 (1 C, *C*‐6 or *C*‐7), 127.7 (1 C, *C*‐6 or *C*‐7), 130.1 (1 C, *C*‐5), 132.6 (1 C, *C*‐4a), 142.5 (1 C, *C*‐8a), 174.1 ppm (1 C, *C*=O). FTIR (neat): *ν* [cm^−1^]=3306 (N−H), 2928, 2862 (C−H_alkyl_), 1636 (C=O), 1543, 1443 (C=C_arom_). Purity (HPLC): 72.3 %, *t*_R_=19.6 min.

### *trans*‐2‐Chloro‐*N*‐(3‐methoxy‐3*H*‐spiro[[2]benzopyran‐1,1′‐cyclohexan]‐4′‐yl)acetamide (*trans*‐20)


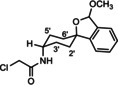


2‐Chloroacetyl chloride (20 μL, 0.25 mmol, 1.2 equiv) was slowly added to a solution of amine *trans*‐**13** (49 mg, 0.21 mmol) and Et_3_N (0.07 mL, 0.50 mmol, 2.4 equiv) in CH_2_Cl_2_ (10 mL) under N_2_ at 0 °C. After stirring for 6 h at RT, H_2_O (30 mL) was added and the aqueous layer was extracted with CH_2_Cl_2_ (3×30 mL). The combined organic layers were dried (Na_2_SO_4_), filtered, concentrated in vacuo and the residue was purified by fc (*d*=2 cm, *l*=20 cm, *V*=5 mL, cyclohexane/ethyl acetate 67 : 33). Colorless solid, m.p. 123 °C, yield 45 mg (69 %). C_16_H_20_ClNO_3_ (309.8 g/mol). *R*_f_=0.50 (cyclohexane/ethyl acetate 50 : 50). HRMS (ESI): *m/z* 332.1037 (calcd. 332.1024 for C_16_H_20_^35^ClNO_3_Na [*M*Na^+^]). ^1^H NMR (600 MHz, CD_3_OD): *δ*=1.61–1.66 (m, 1H, 2′‐*H*), 1.78 (dddd, *J*=13.6/5.7/4.1/1.9 Hz, 1H, 6′‐*H*_equ_), 1.83–1.92 (m, 2H, 3′‐*H*, 5′‐*H*), 1.97–2.15 (m, 4H, 2′‐*H*, 3′‐*H*, 5′‐*H*, 6′‐*H*_ax_), 3.48 (s, 3H, OC*H*_3_), 4.07–4.14 (m, 1H, 4′‐*H*_equ_), 4.11 (s, 2H, COC*H*_2_Cl), 6.05 (s, 1H, 3‐*H*), 7.35–7.38 (m, 2H, 4‐*H*, 5‐*H*), 7.38–7.43 ppm (m, 2H, 6‐*H*, 7‐*H*). A signal for the NH proton is not observed in the spectrum. ^13^C NMR (151 MHz, CD_3_OD): *δ*=27.4 (1 C, *C*‐3′ or *C*‐5′), 27.6 (1 C, *C*‐3′ or *C*‐5′), 34.0 (1 C, *C*‐2′), 35.3 (1 C, *C*‐6′), 43.4 (1 C, CO*C*H_2_Cl), 46.6 (1 C, *C*‐4′), 55.0 (1 C, O*C*H_3_), 87.3 (1 C, *C*‐1), 107.0 (1 C, *C*‐3), 122.1 (1 C, *C*‐7), 124.3 (1 C, *C*‐4), 129.0 (1 C, *C*‐5), 130.4 (1 C, *C*‐6), 138.9 (1 C, *C*‐3a), 148.5 (1 C, *C*‐7a), 169.0 ppm (1 C, *C*=O). FTIR (neat): *ν* [cm^−1^]=3302 (N−H), 2932 (C−H_alkyl_), 1643 (C=O), 1543, 1443 (C=C_arom_). Purity (HPLC): 97.6 %, *t*_R_=14.6 min.

### *cis*‐2‐Chloro‐*N*‐(3‐methoxy‐3*H*‐spiro[[2]benzopyran‐1,1′‐cyclohexan]‐4′‐yl)acetamide (*cis*‐20)


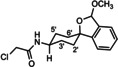


2‐Chloroacetyl chloride (20 μL, 0.25 mmol, 1.2 equiv) was slowly added to a solution of amine *cis*‐**13** (50 mg, 0.21 mmol) and Et_3_N (0.07 mL, 0.50 mmol, 2.4 equiv) in CH_2_Cl_2_ (8 mL) under N_2_ at 0 °C. After stirring for 6 h at RT, H_2_O (30 mL) was added and the aqueous layer was extracted with CH_2_Cl_2_ (3×30 mL). The combined organic layers were dried (Na_2_SO_4_), filtered, concentrated in vacuo and the residue was purified by fc (*d*=2 cm, *l*=18 cm, *V*=10 mL, cyclohexane/ethyl acetate 33 : 67). Colorless solid, m.p. 165 °C, yield 51 mg (78 %). C_16_H_20_ClNO_3_ (309.8 g/mol). *R*_f_=0.48 (cyclohexane/ethyl acetate 50 : 50). HRMS (ESI): *m/z* 332.1014 (calcd. 332.1024 for C_16_H_20_^35^ClNO_3_Na [*M*Na^+^]). ^1^H NMR (600 MHz, CD_3_OD): *δ*=1.69 (dq, *J*=13.6/3.0 Hz, 1H, 2′‐*H*_equ_), 1.82–1.94 (m, 6H, 3′‐*H*, 5′‐*H*, 6′‐*H*), 2.00 (td, *J*=13.5/4.1 Hz, 1H, 2′‐*H*_ax_), 3.49 (s, 3H, OC*H*_3_), 3.87 (tt, *J*=11.1/3.9 Hz, 1H, 4′‐*H*_ax_), 4.03 (s, 2H, COC*H*_2_Cl), 6.07 (s, 1H, 3‐*H*), 7.26 (d, *J*=7.5 Hz, 1H, 7‐*H*), 7.33–7.38 (m, 2H, 4‐*H*, 5‐*H*), 7.39–7.42 ppm (m, 1H, 6‐*H*). A signal for the NH proton is not observed in the spectrum. ^13^C NMR (151 MHz, CD_3_OD): *δ*=29.3 (1 C, *C*‐3′), 29.7 (1 C, *C*‐5′), 37.5 (1 C, *C*‐2′), 38.7 (1 C, *C*‐6′), 43.3 (1 C, CO*C*H_2_Cl), 49.5 (1 C, *C*‐4′), 54.9 (1 C, O*C*H_3_), 86.8 (1 C, *C*‐1), 107.2 (1 C, *C*‐3), 121.8 (1 C, *C*‐7), 124.2 (1 C, *C*‐4), 129.0 (1 C, *C*‐5), 130.5 (1 C, *C*‐6), 138.6 (1 C, *C*‐3a), 148.3 (1 C, *C*‐7a), 168.6 ppm (1 C, *C*=O). FTIR (neat): *ν* [cm^−1^]=3271 (N−H), 2978, 2940, 2866 (C−H_alkyl_), 1651 (C=O), 1555, 1462, 1431 (C=C_arom_). Purity (HPLC): 98.1 %, *t*_R_=15.1 min.

### 1‐(6,7‐Dimethoxy‐3,4‐dihydroisoquinolin‐2(1*H*)‐yl)‐2‐{3‐methoxy‐3,4‐dihydrospiro[[2]benzopyran‐1,4′‐piperidin]‐1′‐yl}ethan‐1‐one (21)


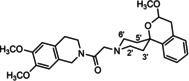


A solution of piperidine **10** (43 mg, 0.18 mmol), chloroacetamide **15** (58 mg, 0.22 mmol, 1.2 equiv), Et_3_N (0.09 mL, 0.65 mmol, 3.6 equiv) and TBAI (9 mg, 0.02 mmol, 0.1 equiv) in DMF (4 mL) was stirred at RT for 18 h. H_2_O (70 mL) was added and the aqueous layer was extracted with CH_2_Cl_2_ (3×60 mL). The combined organic layers were dried (Na_2_SO_4_), filtered, concentrated in vacuo and the residue was purified twice by fc (*d*=2 cm, *l*=25 cm, *V*=10 mL, CH_2_Cl_2_/CH_3_OH 99 : 1+1 % *N*,*N*‐dimethylethanamine; *d*=1 cm, *l*=20 cm, *V*=3 mL, cyclohexane/ethyl acetate 67 : 33+1 % *N*,*N*‐dimethylethanamine→1 : 1+1 % *N*,*N*‐dimethylethanamine). Pale yellow oil, yield 31 mg (22 %). C_27_H_34_N_2_O_5_ (466.6 g/mol). *R*_f_=0.46 (CH_2_Cl_2_/CH_3_OH 99 : 1+1 % *N*,*N*‐dimethylethanamine). HRMS (APCI): *m/z* 467.2531 (calcd. 467.2540 for C_27_H_35_N_2_O_5_ [*M*H^+^]). ^1^H NMR (400 MHz, CD_3_OD): *δ*=1.70 (dq, *J*=13.5/2.8 Hz, 0.5H, 3′‐*H*_equ_), 1.75–1.85 (m, 1H, 3′‐*H*, 5′‐*H*), 1.95 (dq, *J*=13.9/2.6 Hz, 0.5H, 5′‐*H*_equ_), 2.00–2.09 (m, 1.5H, 3′‐*H*, 5′‐*H*), 2.29 (td, *J*=13.1/4.5 Hz, 0.5H, 3′‐*H*_ax_), 2.57–2.73 (m, 2H, 2′‐*H*, 6′‐*H*), 2.73–3.00 (m, 6H, 4‐*H*, 2′‐*H*, 6′‐*H*, 4‐*H*_isoquinoline_), 3.38–3.52 (m, 2H, COC*H*_2_N), 3.54 (s, 1.5H, 3‐OC*H*_3_), 3.57 (s, 1.5H, 3‐OC*H*_3_), 3.81–3.90 (m, 8H, 3‐*H*_isoquinoline_, 6‐OC*H*_3_, 7‐OC*H*_3_), 4.66 (s, 1H, 1‐*H*_isoquinoline_), 4.80 (s, 1H, 1‐*H*_isoquinoline_), 4.90 (dd, *J*=7.2/3.2 Hz, 0.5H, 3‐*H*), 4.95 (dd, *J*=7.2/3.2 Hz, 0.5H, 3‐*H*), 6.76–6.83 (m, 1.5H, 5‐*H*_isoquinoline_, 8‐*H*_isoquinoline_), 6.86 (s, 0.5H, 8‐*H*_isoquinoline_), 7.01–7.05 (m, 0.5H, 8‐*H*), 7.06–7.14 (m, 1H, 5‐*H*) 7.14–7.26 ppm (m, 2.5H, 6‐*H*, 7‐*H*, 8‐*H*). ^13^C NMR (101 MHz, CD_3_OD): *δ*=28.6 (0.5 C, *C*‐4_isoquinoline_), 29.7 (0.5 C, *C*‐4_isoquinoline_), 35.86 (0.5 C, *C*‐4), 35.89 (0.5 C, *C*‐4), 37.18 (0.5 C, *C*‐5′), 37.20 (0.5 C, *C*‐5′), 39.6 (0.5 C, *C*‐3′), 39.7 (0.5 C, *C*‐3′), 41.7 (0.5 C, *C*‐3_isoquinoline_), 44.4 (0.5 C, *C*‐3_isoquinoline_), 44.9 (0.5 C, *C*‐1_isoquinoline_), 48.2 (0.5 C, *C*‐1_isoquinoline_), 50.2–50.7 (m, 2 C, *C*‐2′, *C*‐6′), 56.2–56.4 (m, 3 C, 3‐O*C*H_3_, 6‐O*C*H_3_, 7‐O*C*H_3_), 61.68 (0.5 C, CO*C*H_2_N), 61.74 (0.5 C, CO*C*H_2_N), 75.2 (0.5 C, *C*‐1), 75.4 (0.5 C, *C*‐1), 97.55 (0.5 C, *C*‐3), 97.59 (0.5 C, *C*‐3), 110.6 (0.5 C, *C*‐8_isoquinoline_), 110.9 (0.5 C, *C*‐8_isoquinoline_), 112.9 (0.5 C, *C*‐5_isoquinoline_), 113.1 (0.5 C, *C*‐5_isoquinoline_), 125.6 (1 C, *C*‐8), 126.1 (1 C, *C*‐8a_isoquinoline_), 126.9 (1 C, *C*‐4a_isoquinoline_), 127.37 (0.5 C, *C*‐7), 127.39 (0.5 C, *C*‐7), 127.56 (0.5 C, *C*‐6), 127.62 (0.5 C, *C*‐6), 129.9 (0.5 C, *C*‐5), 130.0 (0.5 C, *C*‐5), 132.4 (0.5 C, *C*‐4a), 132.5 (0.5 C, *C*‐4a), 141.89 (0.5 C, *C*‐8a), 141.92 (0.5 C, *C*‐8a), 149.0–149.3 (m, 2 C, *C*‐6_isoquinoline_, *C*‐7_isoquinoline_), 170.7 (0.5 C, *C*=O), 170.8 ppm (0.5 C, *C*=O). FTIR (neat): *ν* [cm^−1^]=2978, 2835 (C−H_alkyl_), 1624 (C=O), 1516, 1454 (C=C_arom_). Purity (HPLC): 92.1 %, *t*_R_=17.4 min.

### *cis*‐1‐(6,7‐Dimethoxy‐3,4‐dihydroisoquinolin‐2(1*H*)‐yl)‐2‐[*N*‐(3‐methoxy‐3,4‐dihydrospiro[[2]benzopyran‐1,1′‐cyclohexan]‐4′‐yl)amino]ethan‐1‐one (*cis*‐22)


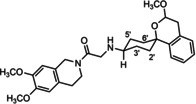


A solution of chloroacetamide **15** (32 mg, 0.12 mmol), amine *cis*‐**11** (36 mg, 0.15 mmol, 1.2 equiv), Et_3_N (0.06 mL, 0.43 mmol, 2.9 equiv) and TBAI (5 mg, 0.01 mmol, 0.1 equiv) in DMF (4 mL) was stirred at RT for 7 d. H_2_O (80 mL) was added and the aqueous layer was extracted with CH_2_Cl_2_ (3×60 mL). The combined organic layers were dried (Na_2_SO_4_), filtered, concentrated in vacuo and the residue was purified by fc (*d*=2 cm, *l*=29 cm, *V*=10 mL, CH_2_Cl_2_/CH_3_OH 99 : 1+1 % *N*,*N*‐dimethylethanamine). Pale yellow oil, yield 35 mg (60 %). C_28_H_36_N_2_O_5_ (480.6 g/mol). *R*_f_=0.29 (CH_2_Cl_2_/CH_3_OH 95 : 5+1 % *N*,*N*‐dimethylethanamine). HRMS (ESI): *m/z* 481.2700 (calcd. 481.2697 for C_28_H_37_N_2_O_5_ [*M*H^+^]). ^1^H NMR (600 MHz, CD_3_OD): *δ*=1.64–1.78 (m, 2H, 2′‐*H*, 3′‐*H*), 1.78–1.92 (m, 4H, 3′‐*H*, 5′‐*H*, 6′‐*H*), 1.92–2.02 (m, 1H, 6′‐*H*), 2.07–2.13 (m, 1H, 2′‐*H*), 2.63–2.72 (m, 1H, 4′‐*H*_ax_), 2.77–2.82 (m, 2H, 4‐*H*, 4‐*H*_isoquinoline_), 2.87 (t, *J*=6.0 Hz, 1H, 4‐*H*_isoquinoline_), 2.89–2.95 (m, 1H, 4‐*H*), 3.56 (s, 1.5H, OC*H*_3_), 3.57 (s, 1.5H, 3‐OC*H*_3_), 3.65 (s, 2H, COC*H*_2_NH), 3.70 (t, *J*=5.9 Hz, 1H, 3‐*H*_isoquinoline_), 3.80–3.83 (m, 1H, 3‐*H*_isoquinoline_), 3.82 (s, 6H, 6‐OC*H*_3_, 7‐OC*H*_3_), 4.61 (s, 1H, 1‐*H*_isoquinoline_), 4.66 (s, 1H, 1‐*H*_isoquinoline_), 4.89–4.92 (m, 1H, 3‐*H*), 6.76–6.79 (m, 1.5H, 5‐*H*_isoquinoline_, 8‐*H*_isoquinoline_), 6.80 (s, 0.5H, 8‐*H*_isoquinoline_), 7.06–7.09 (m, 1H, 5‐*H*), 7.12–7.20 ppm (m, 3H, 6‐*H*, 7‐*H*, 8‐*H*). A signal for the NH proton is not observed in the spectrum. ^13^C NMR (151 MHz, CD_3_OD): *δ*=28.8 (0.5 C, *C*‐4_isoquinoline_), 29.1 (1 C, *C*‐3′ or *C*‐5′), 29.3 (1 C, *C*‐3′ or *C*‐5′), 29.6 (0.5 C, *C*‐4_isoquinoline_), 36.2 (1 C, *C*‐4), 36.4 (1 C, *C*‐2′), 38.9 (1 C, *C*‐6′), 41.6 (0.5 C, *C*‐3_isoquinoline_), 43.6 (0.5 C, *C*‐3_isoquinoline_), 45.2 (0.5 C, *C*‐1_isoquinoline_), 46.9 (0.5 C, *C*‐1_isoquinoline_), 48.1 (0.5 C, CO*C*H_2_NH), 48.3 (0.5 C, CO*C*H_2_NH), 56.4–56.6 (m, 3 C, 3‐O*C*H_3_, 6‐O*C*H_3_, 7‐O*C*H_3_), 57.47 (0.5 C, *C*‐4′), 57.49 (0.5 C, *C*‐4′), 77.37 (0.5 C, *C*‐1), 77.39 (0.5 C, *C*‐1), 97.8 (1 C, *C*‐3), 110.9 (0.5 C, *C*‐8_isoquinoline_), 111.0 (0.5 C, *C*‐8_isoquinoline_), 113.0 (0.5 C, *C*‐5_isoquinoline_), 113.1 (0.5 C, *C*‐5_isoquinoline_), 125.6 (0.5 C, *C*‐8), 125.7 (0.5 C, *C*‐8), 125.8 (0.5 C, *C*‐8a_isoquinoline_), 126.3 (0.5 C, *C*‐8a_isoquinoline_), 127.5 (1 C, *C*‐7), 127.67 (1 C, *C*‐6), 127.74 (0.5 C, *C*‐4a_isoquinoline_), 128.2 (0.5 C, *C*‐4a_isoquinoline_), 130.1 (1 C, *C*‐5), 132.6 (1 C, *C*‐4a), 142.61 (0.5 C, *C*‐8a), 142.62 (0.5 C, *C*‐8a), 149.2–149.6 (m, 2 C, *C*‐6_isoquinoline_, *C*‐7_isoquinoline_), 171.4 (0.5 C, *C*=O), 171.5 ppm (0.5 C, *C*=O). FTIR (neat): *ν* [cm^−1^]=3375 (N−H), 2978, 2936, 2909 (C−H_alkyl_), 1640 (C=O), 1516, 1435 (C=C_arom_). Purity (HPLC): 98.0 %, *t*_R_=17.7 min.

### 1‐(6,7‐Dimethoxy‐3,4‐dihydroisoquinolin‐2(1*H*)‐yl)‐2‐{3‐methoxy‐3H‐spiro[[2]benzofuran‐1,4′‐piperidin]‐1′‐yl}ethan‐1‐one (23)


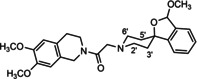


A solution of piperidine **12** (40 mg, 0.18 mmol), chloroacetamide **15** (50 mg, 0.19 mmol, 1.0 equiv), Et_3_N (0.09 mL, 0.65 mmol, 3.6 equiv) and TBAI (7 mg, 0.02 mmol, 0.1 equiv) in DMF (5 mL) was stirred at RT for 19 h. H_2_O (70 mL) was added, and the aqueous layer was extracted with CH_2_Cl_2_ (3×60 mL). The combined organic layers were dried (Na_2_SO_4_), filtered, and concentrated in vacuo, and the residue was purified twice by fc (*d*=2 cm, *l*=25 cm, *V*=10 mL, CH_2_Cl_2_/CH_3_OH 95 : 5; *d*=2 cm, *l*=20 cm, *V*=10 mL, cyclohexane/ethyl acetate 50 : 50+1 % *N*,*N*‐dimethylethanamine). Pale yellow solid, m.p. 103 °C, yield 30 mg (36 %). C_26_H_32_N_2_O_5_ (452.6 g/mol). *R*_f_=0.30 (CH_2_Cl_2_/CH_3_OH 95 : 5+1 % *N*,*N*‐dimethylethanamine). HRMS (APCI): *m/z* 453.2406 (calcd. 453.2384 for C_26_H_33_N_2_O_5_ [*M*H^+^]). ^1^H NMR (600 MHz, CD_3_OD): *δ*=1.52 (dq, *J*=13.5/2.7 Hz, 0.5H, 3′‐*H*_equ_), 1.61 (dq, *J*=13.6/2.8 Hz, 0.5H, 3′‐*H*_equ_), 1.70 (dq, *J*=13.5/2.7 Hz, 0.5H, 5′‐*H*_equ_), 1.78 (dq, *J*=13.6/2.8 Hz, 0.5H, 5′‐*H*_equ_), 1.88–2.00 (m, 1H, 3′‐*H*_ax_, 5′‐*H*_ax_), 2.09 (td, *J*=13.1/4.5 Hz, 0.5H, 5′‐*H*_ax_), 2.17 (td, *J*=13.2/4.5 Hz, 0.5H, 3′‐*H*_ax_), 2.55–2.66 (m, 2H, 2′‐*H*, 6′‐*H*), 2.81 (t, *J*=6.1 Hz, 1H, 4‐*H*_isoquinoline_), 2.84–2.89 (m, 1H, 2′‐*H* or 6′‐*H*), 2.90–2.96 (m, 2H, 2′‐*H* or 6′‐*H*, 4‐*H*_isoquinoline_), 3.40–3.43 (m, 2H, COC*H*_2_N), 3.47 (s, 1.5H, 3‐OC*H*_3_), 3.49 (s, 1.5H, 3‐OC*H*_3_), 3.79–3.87 (m, 8H, 3‐*H*_isoquinoline_, 6‐OC*H*_3_, 7‐OC*H*_3_), 4.65 (s, 1H, 1‐*H*_isoquinoline_), 4.76 (d, *J*=15.9 Hz, 0.5H, 1‐*H*_isoquinoline_), 4.79 (d, *J*=15.9 Hz, 0.5H, 1‐*H*_isoquinoline_), 6.04 (s, 0.5H, 3‐*H*), 6.06 (s, 0.5H, 3‐*H*), 6.77 (s, 1H, 5‐*H*_isoquinoline_, 8‐*H*_isoquinoline_), 6.79 (s, 0.5H, 5‐*H*_isoquinoline_), 6.83 (s, 0.5H, 8‐*H*_isoquinoline_), 7.15 (d, *J*=7.6 Hz, 0.5H, 4‐*H*), 7.29 (d, *J*=7.6 Hz, 0.5H, 4‐*H*), 7.32–7.43 ppm (m, 3H, 5‐*H*, 6‐*H*, 7‐*H*). ^13^C NMR (151 MHz, CD_3_OD): *δ*=28.8 (0.5 C, *C*‐4_isoquinoline_), 30.0 (0.5 C, *C*‐4_isoquinoline_), 38.07 (0.5 C, *C*‐3′), 38.14 (0.5 C, *C*‐3′), 39.60 (0.5 C, *C*‐5′), 39.64 (0.5 C, *C*‐5′), 41.8 (0.5 C, *C*‐3_isoquinoline_), 44.6 (0.5 C, *C*‐3_isoquinoline_), 45.2 (0.5 C, *C*‐1_isoquinoline_), 48.3 (0.5 C, *C*‐1_isoquinoline_), 50.9–51.7 (m, 2 C, *C*‐2′, *C*‐6′), 54.98 (0.5 C, 3‐O*C*H_3_), 55.01 (0.5 C, 3‐O*C*H_3_), 56.5–56.6 (m, 2 C, 6‐O*C*H_3_, 7‐O*C*H_3_), 61.89 (0.5 C, CO*C*H_2_N), 61.92 (0.5 C, CO*C*H_2_N), 85.5 (1 C, *C*‐1), 107.1 (0.5 C, *C*‐3), 107.2 (0.5 C, *C*‐3), 110.9 (0.5 C, *C*‐8_isoquinoline_), 111.0 (0.5 C, *C*‐8_isoquinoline_), 113.1 (0.5 C, *C*‐5_isoquinoline_), 113.3 (0.5 C, *C*‐5_isoquinoline_), 121.7 (0.5 C, *C*‐4), 121.8 (0.5 C, *C*‐4), 124.21 (0.5 C, *C*‐7), 124.25 (0.5 C, *C*‐7), 126.3 (0.5 C, *C*‐8a_isoquinoline_), 126.9 (0.5 C, *C*‐4a_isoquinoline_), 127.8 (0.5 C, *C*‐8a_isoquinoline_), 128.0 (0.5 C, *C*‐4a_isoquinoline_), 129.08 (0.5 C, *C*‐6), 129.12 (0.5 C, *C*‐6), 130.49 (0.5 C, *C*‐5), 130.51 (0.5 C, *C*‐5), 138.86 (0.5 C, *C*‐7a), 138.92 (0.5 C, *C*‐7a), 148.01 (0.5 C, *C*‐3a), 148.02 (0.5 C, *C*‐3a), 149.2–149.5 (m, 2 C, *C*‐6_isoquinoline_, *C*‐7_isoquinoline_), 170.9 (0.5 C, *C*=O), 171.0 ppm (0.5 C, *C*=O). FTIR (neat): *ν* [cm^−1^]=2913, 2735 (C−H_alkyl_), 1636 (C=O), 1516, 1447 (C=C_arom_). Purity (HPLC): 99.7 %, *t*_R_=14.9–17.0 min.

### *trans*‐1‐(6,7‐Dimethoxy‐3,4‐dihydroisoquinolin‐2(1*H*)‐yl)‐2‐[*N*‐(3‐methoxy‐3*H*‐spiro[[2]benzofuran‐1,1′‐cyclohexan]‐4′‐yl)amino]ethan‐1‐one (*trans*‐24)


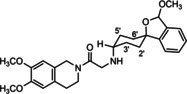


A solution of chloroacetamide **15** (30 mg, 0.11 mmol), amine *trans*‐**13** (26 mg, 0.11 mmol, 1.0 equiv), Et_3_N (0.05 mL, 0.36 mmol, 3.3 equiv.) and TBAI (5 mg, 0.01 mmol, 0.1 equiv) in DMF (4 mL) was stirred at RT for 3 d. H_2_O (80 mL) was added and the aqueous layer was extracted with CH_2_Cl_2_ (3×60 mL). The combined organic layers were dried (Na_2_SO_4_), filtered, concentrated in vacuo and the residue was purified twice by fc (*d*=2 cm, *l*=18 cm, *V*=10 mL, cyclohexane/ethyl acetate 33 : 67+1 % *N*,*N*‐dimethylethanamine→20 : 80+1 % *N*,*N*‐dimethylethanamine; *d*=2 cm, *l*=31 cm, *V*=10 mL, CH_2_Cl_2_/CH_3_OH 95 : 5+1 % *N*,*N*‐dimethylethanamine). Colorless solid, m.p. 66 °C, yield 28 mg (54 %). C_27_H_34_N_2_O_5_ (466.6 g/mol). *R*_f_=0.33 (CH_2_Cl_2_/CH_3_OH 95 : 5+1 % *N*,*N*‐dimethylethanamine). HRMS (ESI): *m/z* 467.2533 (calcd. 467.2540 for C_27_H_35_N_2_O_5_ [*M*H^+^]). ^1^H NMR (600 MHz, CD_3_OD): *δ*=1.52–1.60 (m, 1H, 2′‐*H*), 1.66–1.74 (m, 1H, 6′‐*H*), 1.77–1.86 (m, 2H, 3′‐*H*, 5′‐*H*), 2.01–2.13 (m, 4H, 2′‐*H*, 3′‐*H*, 5′‐*H*, 6′‐*H*), 2.81 (t, *J*=6.0 Hz, 1H, 4‐*H*_isoquinoline_), 2.81 (t, *J*=6.0 Hz, 1H, 4‐*H*_isoquinoline_), 2.90–2.94 (m, 1H, 4′‐*H*_equ_), 3.46 (s, 1.5H, 3‐OC*H*_3_), 3.47 (s, 1.5H, 3‐OC*H*_3_), 3.65 (s, 2H, COC*H*_2_NH), 3.73 (t, *J*=5.9 Hz, 1H, 3‐*H*_isoquinoline_), 3.80–3.86 (m, 7H, 3‐*H*_isoquinoline_, 6‐OC*H*_3_, 7‐OC*H*_3_), 4.67 (s, 1H, 1‐*H*_isoquinoline_), 4.68 (s, 1H, 1‐*H*_isoquinoline_), 6.04 (s, 0.5H, 3‐*H*), 6.05 (s, 0.5H, 3‐*H*), 6.77 (s, 0.5H, 5‐*H*_isoquinoline_), 6.78 (s, 0.5H, 5‐*H*_isoquinoline_), 6.79 (s, 0.5H, 8‐*H*_isoquinoline_), 6.81 (s, 0.5H, 8‐*H*_isoquinoline_), 7.30–7.41 (m, 3.5H, 4‐*H*, 5‐*H*, 6‐*H*, 7‐*H*), 7.44 ppm (d, *J*=7.5 Hz, 0.5H, 7‐*H*). A signal for the NH proton is not observed in the spectrum. ^13^C NMR (151 MHz, CD_3_OD): *δ*=27.9 (0.5 C, *C*‐3′ or *C*‐5′), 28.0 (0.5 C, *C*‐3′ or *C*‐5′), 28.11 (0.5 C, *C*‐3′ or *C*‐5′), 28.14 (0.5 C, *C*‐3′ or *C*‐5′), 28.8 (0.5 C, *C*‐4_isoquinoline_), 29.6 (0.5 C, *C*‐4_isoquinoline_), 34.0 (0.5 C, *C*‐2′), 34.1 (0.5 C, *C*‐2′), 35.2 (0.5 C, *C*‐6′), 35.3 (0.5 C, *C*‐6′), 41.5 (0.5 C, *C*‐3_isoquinoline_), 43.7 (0.5 C, *C*‐3_isoquinoline_), 45.2 (0.5 C, *C*‐1_isoquinoline_), 46.9 (0.5 C, *C*‐1_isoquinoline_), 49.7 (1 C, CO*C*H_2_NH), 54.2 (0.5 C, *C*‐4′), 54.3 (0.5 C, *C*‐4′), 54.8 (1 C, 3‐O*C*H_3_), 56.47 (0.5 C, 6‐O*C*H_3_ or 7‐O*C*H_3_), 56.50 (0.5 C, 6‐O*C*H_3_ or 7‐O*C*H_3_), 56.5 (0.5 C, 6‐O*C*H_3_ or 7‐O*C*H_3_), 56.6 (0.5 C, 6‐O*C*H_3_ or 7‐O*C*H_3_), 88.1 (1 C, *C*‐1), 106.9 (1 C, *C*‐3), 110.9 (0.5 C, *C*‐8_isoquinoline_), 111.0 (0.5 C, *C*‐8_isoquinoline_), 113.0 (0.5 C, *C*‐5_isoquinoline_), 113.1 (0.5 C, *C*‐5_isoquinoline_), 122.4 (0.5 C, *C*‐7), 122.5 (0,5 C, *C*‐7), 124.16 (0.5 C, *C*‐6), 124.19 (0.5 C, *C*‐6), 125.8 (0.5 C, *C*‐8a_isoquinoline_), 126.3 (0.5 C, *C*‐8a_isoquinoline_), 127.8 (0.5 C, *C*‐4a_isoquinoline_), 128.2 (0.5 C, *C*‐4a_isoquinoline_), 128.88 (0.5 C, *C*‐5), 128.90 (0.5 C, *C*‐5), 130.30 (0.5 C, *C*‐4), 130.31 (0.5 C, *C*‐4), 138.7 (0.5 C, *C*‐3a), 138.8 (0.5 C, *C*‐3a), 148.7 (1 C, *C*‐7a), 149.3 (0.5 C, *C*‐6_isoquinoline_ or C‐7_isoquinoline_), 149.38 (0.5 C, *C*‐6_isoquinoline_ or C‐7_isoquinoline_), 149.41 (0.5 C, *C*‐6_isoquinoline_ or C‐7_isoquinoline_), 149.5 (0.5 C, *C*‐6_isoquinoline_ or C‐7_isoquinoline_), 171.7 (0.5 C, *C*=O), 171.8 ppm (0.5 C, *C*=O). FTIR (neat): *ν* [cm^−1^]=3321 (N−H), 2978, 2928 (C−H_alkyl_), 1643 (C=O), 1516, 1435 (C=C_arom_). Purity (HPLC): 95.3 %, *t*_R_=14.5 min.

### *cis*‐1‐(6,7‐Dimethoxy‐3,4‐dihydroisoquinolin‐2(1*H*)‐yl)‐2‐[*N*‐(3‐methoxy‐3H‐spiro[[2]benzofuran‐1,1′‐cyclohexan]‐4′‐yl)amino]ethan‐1‐one (*cis*‐24)


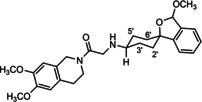


A solution of chloroacetamide **15** (47 mg, 0.17 mmol, 1.3 equiv), amine *cis*‐**13** (32 mg, 0.14 mmol), Et_3_N (0.06 mL, 0.43 mmol, 3.1 equiv.) and TBAI (6 mg, 0.02 mmol, 0.1 equiv) in DMF (4 mL) was stirred at RT for 6 d. H_2_O (80 mL) was added and the aqueous layer was extracted with CH_2_Cl_2_ (3×60 mL). The combined organic layers were dried (Na_2_SO_4_), filtered, concentrated in vacuo and the residue was purified by fc (*d*=2 cm, *l*=29 cm, *V*=10 mL, CH_2_Cl_2_/CH_3_OH 99 : 1+1 % *N*,*N*‐dimethylethanamine). Yellow oil, yield 28 mg (43 %). C_27_H_34_N_2_O_5_ (466.6 g/mol). *R*_f_=0.23 (CH_2_Cl_2_/CH_3_OH 95 : 5+1 % *N*,*N*‐dimethylethanamine). HRMS (ESI): *m/z* 467.2534 (calcd. 467.2540 for C_27_H_35_N_2_O_5_ [*M*H^+^]). ^1^H NMR (600 MHz, CD_3_OD): *δ*=1.64–1.70 (m, 1H, 2′‐*H*), 1.72–1.83 (m, 3H, 3′‐*H*, 5′‐*H*, 6′‐*H*), 1.83–1.93 (m, 2H, 2′‐*H*, 6′‐*H*), 1.93–2.00 (m, 2H, 3′‐*H*, 5′‐*H*), 2.64–2.72 (m, 1H, 4′‐*H*_ax_), 2.81 (t, *J*=6.2 Hz, 1H, 4‐*H*_isoquinoline_), 2.87 (t, *J*=6.0 Hz, 1H, 4‐*H*_isoquinoline_), 3.49 (s, 1.5H, 3‐OC*H*_3_), 3.49 (s, 1.5H, 3‐OC*H*_3_), 3.67 (s, 2H, COC*H*_2_N), 3.70 (t, *J*=6.0 Hz, 1H, 3‐*H*_isoquinoline_), 3.80–3.85 (m, 7H, 3‐*H*_isoquinoline_, 6‐OC*H*_3_, 7‐OC*H*_3_), 4.61 (s, 1H, 1‐*H*_isoquinoline_), 4.66 (s, 1H, 1‐*H*_isoquinoline_), 6.04 (s, 0.5H, 3‐*H*), 6.05 (s, 0.5H, 3‐*H*), 6.76–6.79 (m, 1.5H, 5‐*H*_isoquinoline_, 8‐*H*_isoquinoline_), 6.80 (s, 0.5H, 8‐*H*_isoquinoline_), 7.18–7.25 (m, 1H, 7‐*H*), 7.31–7.40 ppm (m, 3H, 4‐*H*, 5‐*H*, 6‐*H*). A signal for the NH proton is not observed in the spectrum. ^13^C NMR (151 MHz, CD_3_OD): *δ*=28.8 (0.5 C, *C*‐4_isoquinoline_), 29.6 (0.5 C, *C*‐4_isoquinoline_), 29.7 (1 C, *C*‐3′ or *C*‐5′), 30.0 (1 C, *C*‐3′ or *C*‐5′), 37.3 (1 C, *C*‐2′), 38.7 (1 C, C‐6′), 41.6 (0.5 C, *C*‐3_isoquinoline_), 43.6 (0.5 C, *C*‐3_isoquinoline_), 45.2 (0.5 C, *C*‐1_isoquinoline_), 46.8 (0.5 C, *C*‐1_isoquinoline_), 48.0 (0.5 C, CO*C*H_2_N), 48.2 (0.5 C, CO*C*H_2_N), 55.0 (1 C, 3‐O*C*H_3_), 56.5–56.6 (m, 2 C, 6‐O*C*H_3_, 7‐O*C*H_3_), 57.2 (1 C, *C*‐4′), 87.30 (0.5 C, *C*‐1), 87.32 (0.5 C, *C*‐1), 107.1 (1 C, *C*‐3), 110.9 (0.5 C, *C*‐8_isoquinoline_), 111.0 (0.5 C, *C*‐8_isoquinoline_), 113.0 (0.5 C, *C*‐5_isoquinoline_), 113.1 (0.5 C, *C*‐5_isoquinoline_), 121.68 (0.5 C, *C*‐7), 121.70 (0.5 C, *C*‐7), 124.2 (1 C, *C*‐4), 125.7 (0.5 C, *C*‐8a_isoquinoline_), 126.3 (0.5 C, *C*‐8a_isoquinoline_), 127.7 (0.5 C, *C*‐4a_isoquinoline_), 128.2 (0.5 C, *C*‐4a_isoquinoline_), 129.0 (1 C, *C*‐5), 130.4 (1 C, *C*‐6), 138.8 (1 C, *C*‐3a), 148.5 (1 C, *C*‐7a), 149.2–149.6 (m, 2 C, *C*‐6_isoquinoline_, *C*‐7_isoquinoline_), 171.2 (0.5 C, *C*=O), 171.3 ppm (0.5 C, *C*=O). FTIR (neat): *ν* [cm^−1^]=3402 (N−H), 2928, 2855 (C−H_alkyl_), 1643 (C=O), 1516, 1435 (C=C_arom_). Purity (HPLC): 98.1 %, *t*_R_=15.1 min.

### 2‐(6,7‐Dimethoxy‐3,4‐dihydroisoquinolin‐2(1*H*)‐yl)‐1‐(3‐methoxy‐3,4‐dihydrospiro[[2]benzopyran‐1,4′‐piperidin]‐1′‐yl)ethan‐1‐one (25)


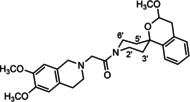


2‐Chloroacetyl chloride (19 μL, 0.24 mmol, 1.2 equiv) was slowly added to a solution of piperidine **10** (46 mg, 0.20 mmol) and Et_3_N (0.07 mL, 0.50 mmol, 2.5 equiv) in CH_2_Cl_2_ (4 mL) under N_2_ at 0 °C. After 5 h of stirring at RT, H_2_O (10 mL) was added, and the aqueous layer was extracted with CH_2_Cl_2_ (3×10 mL). The combined organic layers were dried (Na_2_SO_4_), filtered, and concentrated in vacuo, and the residue was purified by fc (*d*=2 cm, *l*=18 cm, *V*=10 mL, cyclohexane/ethyl acetate 67 : 33→50 : 50). Chloroacetamide **16**: Pale yellow oil, yield 25 mg (40 %). C_16_H_20_ClNO_3_ (309.8 g/mol).

A solution of chloroacetamide **16** (25 mg, 0.08 mmol), isoquinoline **14**⋅HCl (20 mg, 0.09 mmol, 1.1 equiv), Et_3_N (0.03 mL, 0.22 mmol, 2.8 equiv) and TBAI (5 mg, 0.01 mmol, 0.1 equiv) in DMF (4 mL) was stirred at RT for 63 h. H_2_O (80 mL) was added and the aqueous layer was extracted with CH_2_Cl_2_ (3×60 mL). The combined organic layers were dried (Na_2_SO_4_), filtered, concentrated in vacuo and the residue was purified by fc (*d*=2 cm, *l*=20 cm, *V*=10 mL, cyclohexane/ethyl acetate 50 : 50+1 % *N*,*N*‐dimethylethanamine). Pale yellow solid, m.p. 165 °C, yield 30 mg (75 %). C_27_H_34_N_2_O_5_ (466.6 g/mol). *R*_f_=0.26 (cyclohexane/ethyl acetate 50 : 50+1 % *N*,*N*‐dimethylethanamine). HRMS (APCI): *m/z* 467.2521 (calcd. 467.2540 for C_27_H_35_N_2_O_5_ [*M*H^+^]). ^1^H NMR (600 MHz, CD_3_OD): *δ*=1.76–1.86 (m, 1.5H, 3′‐*H*, 5′‐*H*), 1.90–1.96 (m, 0.5H, 5′‐*H*), 1.99–2.10 (m, 1.5H, 3′‐*H*, 5′‐*H*), 2.17 (td, *J*=13.3/4.6 Hz, 0.5H, 5′‐*H*), 2.79–2.93 (m, 5H, 4‐*H*, 3‐*H*_isoquinoline_, 4‐*H*_isoquinoline_), 2.93–2.98 (m, 1H, 4‐*H*), 3.13–3.23 (m, 1H, 2′‐*H*), 3.37 (d, *J*=14.0 Hz, 1H, COC*H*_2_N), 3.54 (s, 1.5H, 3‐OC*H*_3_), 3.55 (s, 1.5H, 3‐OC*H*_3_), 3.56–3.66 (m, 3H, 6′‐*H*, COC*H*_2_N, 1‐*H*_isoquinoline_), 3.67–3.72 (m, 1H, 1‐*H*_isoquinoline_), 3.79–3.82 (m, 6H, 6‐OC*H*_3_, 7‐OC*H*_3_), 4.09–4.14 (m, 1H, 6′‐*H*), 4.50–4.57 (m, 1H, 2′‐*H*), 4.96 (dd, *J*=6.9/3.2 Hz, 0.5H, 3‐*H*), 4.98 (dd, *J*=7.0/3.2 Hz, 0.5H, 3‐*H*), 6.69 (s, 0.5H, 8‐*H*_isoquinoline_), 6.69 (s, 0.5H, 8‐*H*_isoquinoline_), 6.70 (s, 0.5H, 5‐*H*_isoquinoline_), 6.72 (s, 0.5H, 5‐*H*_isoquinoline_), 6.94–6.97 (m, 0.5H, 8‐*H*), 7.00 (dd, *J*=7.5/1.6 Hz, 0.5H, 8‐*H*), 7.08–7.17 ppm (m, 3H, 5‐*H*, 6‐*H*, 7‐*H*). ^13^C NMR (151 MHz, CD_3_OD): *δ*=29.5 (0.5 C, *C*‐4_isoquinoline_), 29.6 (0.5 C, *C*‐4_isoquinoline_), 36.0 (1 C, *C*‐4), 37.6 (0.5 C, *C*‐3′), 38.2 (0.5 C, *C*‐5′), 39.6 (0.5 C, *C*‐2′), 39.7 (0.5 C, *C*‐2′), 39.8 (0.5 C, *C*‐3′), 40.4 (0.5 C, *C*‐5′), 43.48 (0.5 C, *C*‐6′), 43.50 (0.5 C, *C*‐6′), 52.2 (0.5 C, *C*‐3_isoquinoline_), 52.3 (0.5 C, *C*‐3_isoquinoline_), 56.4–56.6 (m, 4 C, *C*‐1_isoquinoline_, 3‐O*C*H_3_, 6‐O*C*H_3_, 7‐O*C*H_3_), 61.28 (0.5 C, CO*C*H_2_N), 61.30 (0.5 C, CO*C*H_2_N), 76.08 (0.5 C, *C*‐1), 76.11 (0.5 C, *C*‐1), 98.15 (0.5 C, *C*‐3), 98.22 (0.5 C, *C*‐3), 111.1 (1 C, *C*‐8_isoquinoline_), 113.1 (0.5 C, *C*‐5_isoquinoline_), 113.2 (0.5 C, *C*‐5_isoquinoline_), 125.7 (1 C, *C*‐8), 127.40 (1 C, *C*‐4a_sioquinoline_ or *C*‐8a_isoquinoline_), 127.44 (1 C, *C*‐4a_sioquinoline_ or *C*‐8a_isoquinoline_), 127.6 (0.5 C, *C*‐7), 127.7 (0.5 C, *C*‐7), 128.0 (1 C, *C*‐6), 130.2 (1 C, *C*‐5), 132.6 (1 C, *C*‐4a), 141.32 (0.5 C, *C*‐8a), 141.34 (0.5 C, *C*‐8a), 148.8 (1 C, *C*‐6_sioquinoline_ or *C*‐7_isoquinoline_), 149.2 (1 C, *C*‐6_sioquinoline_ or *C*‐7_isoquinoline_), 170.4 (0.5 C, *C*=O), 170.5 ppm (0.5 C, *C*=O). FTIR (neat): *ν* [cm^−1^]=2924, 2835 (C−H_alkyl_), 1639 (C=O), 1516, 1443 (C=C_arom_). Purity (HPLC): 99.4 %, *t*_R_=17.4 min.

### *trans*‐2‐(6,7‐Dimethoxy‐3,4‐dihydroisoquinolin‐2(1*H*)‐yl)‐*N*‐(3‐methoxy‐3,4‐dihydrospiro[[2]benzopyran‐1,1′‐cyclohexan]‐4′‐yl)acetamide (*trans*‐26)


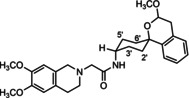


A solution of chloroacetamide *trans*‐**17** (29 mg, 0.09 mmol), isoquinoline **14**⋅HCl (23 mg, 0.10 mmol, 1.1 equiv), Et_3_N (0.04 mL, 0.29 mmol, 3.2 equiv) and TBAI (3 mg, 0.01 mmol, 0.1 equiv) in DMF (4 mL) was stirred at RT for 68 h. H_2_O (80 mL) was added and the aqueous layer was extracted with CH_2_Cl_2_ (3×60 mL). The combined organic layers were dried (Na_2_SO_4_), filtered, concentrated in vacuo and the residue was purified twice by fc (*d*=2 cm, *l*=18 cm, *V*=10 mL, cyclohexane/ethyl acetate 50 : 50+1 % *N*,*N*‐dimethylethanamine; *d*=2 cm, *l*=20 cm, *V*=10 mL, cyclohexane/ethyl acetate 50 : 50+1 % *N*,*N*‐dimethylethanamine). Colorless oil, yield 36 mg (83 %). C_28_H_36_N_2_O_5_ (480.6 g/mol). *R*_f_=0.11 (cyclohexane/ethyl acetate 50 : 50+1 % *N*,*N*‐dimethylethanamine). HRMS (ESI): *m/z* 481.2695 (calcd. 481.2697 for C_28_H_37_N_2_O_5_ [*M*H^+^]). ^1^H NMR (600 MHz, CD_3_OD): *δ*=1.66–1.72 (m, 2H, 2′‐*H*, 6′‐*H*), 1.73–1.78 (m, 2H, 3′‐*H*, 5′‐*H*), 1.90–1.95 (m, 1H, 6′‐*H*), 1.96–2.00 (m, 1H, 2′‐*H*), 2.10–2.17 (m, 1H, 5′‐*H*), 2.17–2.24 (m, 1H, 3′‐*H*), 2.77 (dd, *J*=15.7/7.3 Hz, 1H, 4‐*H*), 2.90 (dd, *J*=15.7/3.1 Hz, 1H, 4‐*H*), 2.92–2.95 (m, 2H, 3‐*H*_isoquinoline_), 2.98 (t, *J*=5.6 Hz, 2H, 4‐*H*_isoquinoline_), 3.30 (s, 2H, COC*H*_2_N), 3.53 (s, 3H, 3‐OC*H*_3_), 3.72 (s, 5H, 1‐*H*_isoquinoline_, 7‐OC*H*_3_), 3.85 (s, 3H, 6‐OC*H*_3_,), 4.19 (quint, *J*=3.3 Hz, 1H, 4′‐*H*_equ_), 4.88 (dd, *J*=7.4/3.1 Hz, 1H, 3‐*H*), 6.68 (s, 1H, 8‐*H*_isoquinoline_), 6.71 (dd, *J*=7.9/1.2 Hz, 1H, 8‐*H)*, 6.80 (s, 1H, 5‐*H*_isoquinoline_), 6.89–6.92 (m, 1H, 7‐*H*), 7.05 (dd, *J*=7.6/1.3 Hz, 1H, 5‐*H*), 7.10 ppm (td, *J*=7.4/1.2 Hz, 1H, 6‐*H*). A signal for the NH proton is not observed in the spectrum. ^13^C NMR (151 MHz, CD_3_OD): *δ*=26.5 (1 C, *C*‐3′), 26.6 (1 C, *C*‐5′), 30.1 (1 C, *C*‐4_isoquinoline_), 32.2 (1 C, *C*‐6′), 34.6 (1 C, *C*‐2′), 36.1 (1 C, *C*‐4), 44.6 (1 C, *C*‐4′), 52.7 (1 C, *C*‐3_isoquinoline_), 56.35 (1 C, 3‐O*C*H_3_), 56.44 (1 C, 7‐O*C*H_3_), 56.5 (1 C, 6‐O*C*H_3_), 56.8 (1 C, *C*‐1_isoquinoline_), 62.3 (1 C, CO*C*H_2_N), 77.1 (1 C, *C*‐1), 97.9 (1 C, *C*‐3), 111.1 (1 C, *C*‐8_isoquinoline_), 113.2 (1 C, *C*‐5_isoquinoline_), 125.4 (1 C, *C*‐8), 127.1 (1 C, *C*‐4a_isoquinoline_), 127.4 (1 C, *C*‐8a_isoquinoline_), 127.5 (1 C, *C*‐7), 127.7 (1 C, *C*‐6), 130.1 (1 C, *C*‐5), 132.4 (1 C, *C*‐4a), 142.6 (1 C, *C*‐8a), 148.9 (1 C, *C*‐7_isoquinoline_), 149.4 (1 C, *C*‐6_isoquinoline_), 172.1 ppm (1 C, *C*=O). FTIR (neat): *ν* [cm^−1^]=3341 (N−H), 2928, 2832 (C−H_alkyl_), 1674 (C=O), 1516, 1447 (C=C_arom_). Purity (HPLC): 90.6 %, *t*_R_=17.0 min.

### *cis*‐2‐(6,7‐Dimethoxy‐3,4‐dihydroisoquinolin‐2(1*H*)‐yl)‐*N*‐(3‐methoxy‐3,4‐dihydrospiro[[2]benzopyran‐1,1′‐cyclohexan]‐4′‐yl)acetamide (*cis*‐26)


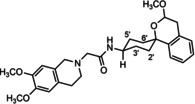


A solution of chloroacetamide *cis*‐**17** (34 mg, 0.10 mmol), isoquinoline **14**⋅HCl (26 mg, 0.11 mmol, 1.1 equiv), Et_3_N (0.04 mL, 0.29 mmol, 2.9 equiv) and TBAI (4 mg, 0.01 mmol, 0.1 equiv) in DMF (4 mL) was stirred at RT for 66 h. H_2_O (80 mL) was added and the aqueous layer was extracted with CH_2_Cl_2_ (3×60 mL). The combined organic layers were dried (Na_2_SO_4_), filtered, concentrated in vacuo and the residue was purified by fc (*d*=2 cm, *l*=20 cm, *V*=10 mL, cyclohexane/ethyl acetate 50 : 50+1 % *N*,*N*‐dimethylethanamine). Colorless solid, m.p. 176 °C, yield 31 mg (62 %). C_28_H_36_N_2_O_5_ (480.6 g/mol). *R*_f_=0.08 (cyclohexane/ethyl acetate 50 : 50+1 % *N*,*N*‐dimethylethanamine). HRMS (APCI): *m/z* 481.2720 (calcd. 481.2697 for C_28_H_37_N_2_O_5_ [*M*H^+^]). ^1^H NMR (600 MHz, CD_3_OD): *δ*=1.77–1.95 (m, 6H, 2′‐*H*, 3′‐*H*, 5′‐*H*, 6′‐*H*), 2.05–2.14 (m, 2H, 2′‐*H*, 6′‐*H*), 2.77–2.84 (m, 3H, 4‐*H*, 3‐*H*_isoquinoline_), 2.86–2.90 (m, 2H, 4‐*H*_isoquinoline_), 2.93 (dd, *J*=15.7/3.1 Hz, 1H, 4‐*H*), 3.22 (s, 2H, COC*H*_2_Cl), 3.54 (s, 3H, 3‐OC*H*_3_), 3.67 (s, 2H, 1‐*H*_isoquinoline_), 3.80 (s, 3H, 7‐OC*H*_3_), 3.81 (s, 3H, 6‐OC*H*_3_), 3.88–3.95 (m, 1H, 4′‐*H*_ax_), 4.91 (dd, *J*=7.4/3.1 Hz, 1H, 3‐*H*), 6.65 (s, 1H, 8‐*H*_isoquinoline_), 6.72 (s, 1H, 5‐*H*_isoquinoline_), 7.09 (d, *J*=7.4 Hz, 1H, 5‐*H*), 7.16 (td, *J*=7.2/1.8 Hz, 1H, 6‐*H*), 7.18–7.24 ppm (m, 2H, 7‐*H*, 8‐*H*). A signal for the NH proton is not observed in the spectrum. ^13^C NMR (151 MHz, CD_3_OD): *δ*=29.0 (1 C, *C*‐3′ or *C*‐5′), 29.2 (1 C, *C*‐3′ or *C*‐5′), 29.4 (1 C, *C*‐4_isoquinoline_), 36.1 (1 C, *C*‐4), 36.6 (1 C, *C*‐2′), 39.1 (1 C, *C*‐6′), 49.0 (1 C, *C*‐4′), 52.3 (1 C, *C*‐3_isoquinoline_), 56.4 (2 C, *C*‐1_isoquinoline_, 3‐O*C*H_3_), 56.47 (1 C, 6‐O*C*H_3_), 56.51 (1 C, 7‐O*C*H_3_), 62.0 (1 C, CO*C*H_2_N), 76.9 (1 C, *C*‐1), 97.9 (1 C, *C*‐3), 111.1 (1 C, *C*‐8_isoquinoline_), 113.1 (1 C, *C*‐5_isoquinoline_), 125.7 (1 C, *C*‐8), 127.2 (1 C, *C*‐4a_isoquinoline_), 127.5 (1 C, *C*‐8a_isoquinoline_), 127.6 (1 C, *C*‐7), 127.7 (1 C, *C*‐6), 130.1 (1 C, *C*‐5), 132.6 (1 C, *C*‐4a), 142.5 (1 C, *C*‐8a), 148.9 (1 C, *C*‐7_isoquinoline_), 149.2 (1 C, *C*‐6_isoquinoline_), 171.9 ppm (1 C, *C*=O). FTIR (neat): *ν* [cm^−1^]=3298 (N−H), 2978, 2924, 2835 (C−H_alkyl_), 1639 (C=O), 1512, 1443 (C=C_arom_). Purity (HPLC): 98.1 %, *t*_R_=17.5 min.

### *cis*‐4‐(6,7‐Dimethoxy‐3,4‐dihydroisoquinolin‐2(1*H*)‐yl)‐*N*‐(3‐methoxy‐3,4‐dihydrospiro[[2]benzopyran‐1,1′‐cyclohexan]‐4′‐yl)butanamide (*cis*‐27)


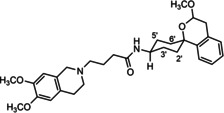


A solution of isoquinoline **14**⋅HCl (27 mg, 0.12 mmol), chlorobutyramide *cis*‐**18** (45 mg, 0.13 mmol, 1.1 equiv), Et_3_N (0.05 mL, 0.36 mmol, 3.0 equiv) and TBAI (5 mg, 0.01 mmol, 0.1 equiv) in DMF (4 mL) was stirred at RT for 6 d. H_2_O (80 mL) was added and the aqueous layer was extracted with CH_2_Cl_2_ (3×60 mL). The combined organic layers were dried (Na_2_SO_4_), filtered, concentrated in vacuo and the residue was purified twice by fc (*d*=2 cm, *l*=32 cm, *V*=10 mL, CH_2_Cl_2_/CH_3_OH 99 : 1+1 % *N*,*N*‐dimethylethanamine; *d*=1 cm, *l*=25 cm, *V*=3 mL, CH_2_Cl_2_/CH_3_OH 95 : 5). Pale yellow oil, yield 11 mg (19 %). C_30_H_40_N_2_O_5_ (508.7 g/mol). *R*_f_=0.19 (CH_2_Cl_2_/CH_3_OH 95 : 5). HRMS (ESI): *m/z* 509.3024 (calcd. 509.3010 for C_30_H_41_N_2_O_5_ [*M*H^+^]). ^1^H NMR (400 MHz, CD_3_OD): *δ*=1.74–1.91 (m, 6H, 2′‐*H*, 3′‐*H*, 5′‐*H*, 6′‐*H*), 1.97 (quint, *J*=7.5 Hz, 2H, COCH_2_C*H*_2_CH_2_N), 2.02–2.09 (m, 1H, 6′‐*H*), 2.09–2.15 (m, 1H, 2′‐*H*), 2.31 (t, *J*=7.3 Hz, 2H, COC*H*_2_CH_2_CH_2_N), 2.65 (t, *J*=7.8 Hz, 2H, COCH_2_CH_2_C*H*_2_N), 2.78–2.88 (m, 3H, 4‐*H*, 3‐*H*_isoquinoline_), 2.88–2.92 (m, 2H, 4‐*H*_isoquinoline_), 2.95 (dd, *J*=15.7/3.2 Hz, 1H, 4‐*H*), 3.58 (s, 3H, 3‐OC*H*_3_), 3.68 (s, 2H, 1‐*H*_isoquinoline_), 3.79–3.90 (m, 1H, 4′‐*H*_ax_), 3.817 (s, 3H, 6‐OC*H*_3_ or 7‐OC*H*_3_), 3.818 (s, 3H, 6‐OC*H*_3_ or 7‐OC*H*_3_), 4.93 (dd, *J*=7.5/3.2 Hz, 1H, 3‐*H*), 6.69 (s, 1H, 8‐*H*_isoquinoline_), 6.73 (s, 1H, 5‐*H*_isoquinoline_), 7.08–7.13 (m, 1H, 5‐*H*), 7.15–7.20 (m, 1H, 6‐*H*), 7.20–7.24 ppm (m, 2H, 7‐*H*, 8‐*H*). A signal for the NH proton is not observed in the spectrum. ^13^C NMR (101 MHz, CD_3_OD): *δ*=23.8 (1 C, COCH_2_ *C*H_2_CH_2_N), 28.8 (1 C, *C*‐4_isoquinoline_), 29.0 (1 C, *C*‐3′ or *C*‐5′), 29.1 (1 C, *C*‐3′ or *C*‐5′), 35.0 (1 C, CO*C*H_2_CH_2_CH_2_N), 36.2 (1 C, *C*‐4), 36.6 (1 C, *C*‐2′), 39.1 (1 C, *C*‐6′), 49.1 (1 C, *C*‐4′), 52.0 (1 C, *C*‐3_isoquinoline_), 56.4 (1 C, *C*‐1_isoquinoline_), 56.4 (1 C, 3‐O*C*H_3_), 56.47 (1 C, 6‐O*C*H_3_ or 7‐O*C*H_3_), 56.53 47 (1 C, 6‐O*C*H_3_ or 7‐O*C*H_3_), 58.4 (1 C, COCH_2_CH_2_ *C*H_2_N), 77.0 (1 C, *C*‐1), 97.9 (1 C, *C*‐3), 111.2 (1 C, *C*‐8_isoquinoline_), 113.0 (1 C, *C*‐5_isoquinoline_), 125.7 (1 C, *C*‐8), 126.9 (1 C, *C*‐8a_isoquinoline_), 127.1 (1 C, *C*‐4a_isoquinoline_), 127.6 (1 C, *C*‐7), 127.7 (1 C, *C*‐6), 130.1 (1 C, *C*‐5), 132.6 (1 C, *C*‐4a), 142.5 (1 C, *C*‐8a), 149.0 (1 C, *C*‐7_isoquinoline_), 149.4 (1 C, *C*‐6_isoquinoline_), 174.8 ppm (1 C, *C*=O). FTIR (neat): *ν* [cm^−1^]=3275 (N−H), 2924, 2855 (C−H_alkyl_), 1639 (C=O), 1516, 1447 (C=C_arom_). Purity (HPLC): 93.5 %, *t*_R_=17.9 min.

### 2‐(6,7‐Dimethoxy‐3,4‐dihydroisoquinolin‐2(1*H*)‐yl)‐1‐{3‐methoxy‐3*H*‐spiro[[2]benzofuran‐1,4′‐piperidin]‐1′‐yl}ethan‐1‐one (28)


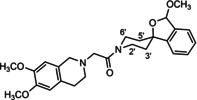


2‐Chloroacetyl chloride (36 μL, 0.45 mmol, 1.2 equiv) was slowly added to a solution of piperidine **12** (83 mg, 0.38 mmol) and Et_3_N (0.12 mL, 0.87 mmol, 2.3 equiv) in CH_2_Cl_2_ (10 mL) under N_2_ at 0 °C. After stirring for 6 h at RT, H_2_O (10 mL) was added and the aqueous layer was extracted with CH_2_Cl_2_ (3×10 mL). The combined organic layers were dried (Na_2_SO_4_), filtered, concentrated in vacuo and the residue was purified by fc (*d*=2 cm, *l*=19 cm, *V*=10 mL, cyclohexane/ethyl acetate 67 : 33). Chloroacetamide **19**: Pale yellow oil, yield 50 mg (44 %). C_15_H_18_ClNO_3_ (295.8 g/mol).

A solution of chloroacetamide **19** (50 mg, 0.17 mmol, 1.2 equiv), isoquinoline **14**⋅HCl (33 mg, 0.14 mmol), Et_3_N (0.07 mL, 0.50 mmol, 3.6 equiv) and TBAI (6 mg, 0.02 mmol, 0.1 equiv) in DMF (6 mL) was stirred at RT for 5 d. H_2_O (70 mL) was added and the aqueous layer was extracted with CH_2_Cl_2_ (4×30 mL). The combined organic layers were dried (Na_2_SO_4_), filtered, concentrated in vacuo and the residue was purified twice by fc (*d*=2 cm, *l*=25 cm, *V*=10 mL, CH_2_Cl_2_/CH_3_OH 95 : 5; *d*=2 cm, *l*=20 cm, *V*=10 mL, cyclohexane/ethyl acetate 50 : 50+1 % *N*,*N*‐dimethylethanamine). Pale yellow oil, yield 31 mg (49 %). C_26_H_32_N_2_O_5_ (452.6 g/mol). *R*_f_=0.16 (cyclohexane/ethyl acetate 50 : 50+1 % *N*,*N*‐dimethylethanamine). HRMS (APCI): *m/z* 453.2403 (calcd. 453.2384 for C_26_H_33_N_2_O_5_ [*M*H^+^]). ^1^H NMR (600 MHz, CD_3_OD): *δ*=1.59–1.67 (m, 1H, 3′‐*H*, 5′‐*H*), 1.77–1.84 (m, 1H, 3′‐*H*, 5′‐*H*), 1.88–1.94 (m, 0.5H, 3′‐*H*), 1.96–2.05 (m, 1H, 3′‐*H*, 5′‐*H*), 2.09 (td, *J*=13.3/4.8 Hz, 0.5H, 5′‐*H*), 2.82–2.85 (m, 2H, 3‐*H*_isoquinoline_), 2.86–2.90 (m, 2H, 4‐*H*_isoquinoline_), 3.13–3.19 (m, 1H, 2′‐*H*), 3.40 (d, *J*=8.3 Hz, 0.5H, COC*H*_2_N), 3.42 (d, *J*=8.3 Hz, 0.5H, COC*H*_2_N), 3.50 (s, 1.5H, 3‐OC*H*_3_), 3.51 (s, 1.5H, 3‐OC*H*_3_), 3.53–3.61 (m, 2H, 6′‐*H*, COC*H*_2_N), 3.66 (s, 1H, 1‐*H*_isoquinoline_), 3.67 (s, 1H, 1‐*H*_isoquinoline_), 3.79–3.82 (m, 6H, 6‐OC*H*_3_, 7‐OC*H*_3_), 4.16–4.21 (m, 1H, 6′‐*H*), 4.56–4.62 (m, 1H, 2′‐*H*), 6.09 (s, 0.5H, 3‐*H*), 6.10 (s, 0.5H, 3‐*H*), 6.67 (s, 0.5H, 8‐*H*_isoquinoline_), 6.68 (s, 0.5H, 8‐*H*_isoquinoline_), 6.71 (s, 0.5H, 5‐*H*_isoquinoline_), 6.72 (s, 0.5H, 5‐*H*_isoquinoline_), 7.11–7.13 (m, 0.5H, 4‐*H*), 7.15–7.17 (m, 0.5H, 4‐*H*), 7.34–7.40 ppm (m, 3H, 5‐*H*, 6‐*H*, 7‐*H*). ^13^C NMR (151 MHz, CD_3_OD): *δ*=29.47 (0.5 C, *C*‐4_isoquinoline_), 29.51 (0.5 C, *C*‐4_isoquinoline_), 38.0 (0.5 C, *C*‐3′), 38.7 (0.5 C, *C*‐5′), 39.5 (0.5 C, *C*‐3′), 40.1 (1 C, *C*‐2′, *C*‐5′), 40.5 (0.5 C, *C*‐2′), 43.9 (0.5 C, *C*‐6′), 44.2 (0.5 C, *C*‐6′), 52.2 (1 C, *C*‐3_isoquinoline_), 55.2 (1 C, 3‐O*C*H_3_), 56.4–56.6 (m, 3 C, *C*‐1_isoquinoline_, 6‐O*C*H_3_, 7‐O*C*H_3_), 61.1 (0.5 C, CO*C*H_2_N), 61.2 (0.5 C, CO*C*H_2_N), 85.9 (1 C, *C*‐1), 107.4 (1 C, *C*‐3), 111.1 (1 C, *C*‐8_isoquinoline_), 113.11 (0.5 C, *C*‐5_isoquinoline_), 113.13 (0.5 C, *C*‐5_isoquinoline_), 121.75 (0.5 C, *C*‐4), 121.77 (0.5 C, *C*‐4), 124.4 (1 C, *C*‐7), 127.38 (0.5 C, *C*‐4a_isoquinoline_), 127.40 (0.5 C, *C*‐4a_isoquinoline_), 127.6 (0.5 C, *C*‐8a_isoquinoline_), 127.7 (0.5 C, *C*‐8a_isoquinoline_), 129.3 (1 C, *C*‐6), 130.58 (0.5 C, *C*‐5), 130.60 (0.5 C, *C*‐5), 138.9 (1 C, *C*‐7a), 147.3 (1 C, *C*‐3a), 148.82 (0.5 C, *C*‐7_isoquinoline_), 148.83 (0.5 C, *C*‐7_isoquinoline_), 149.16 (0.5 C, *C*‐6_isoquinoline_), 149.17 (0.5 C, *C*‐6_isoquinoline_), 170.39 (0.5 C, *C*=O), 170.40 ppm (0.5 C, C=O). FTIR (neat): *ν* [cm^−1^]=2913, 2735 (C−H_alkyl_), 1636 (C=O), 1516, 1447 (C=C_arom_). Purity (HPLC): 98.7 %, *t*_R_=14.3–16.7 min.

### *trans*‐2‐(6,7‐Dimethoxy‐3,4‐dihydroisoquinolin‐2(1*H*)‐yl)‐*N*‐(3‐methoxy‐3*H*‐spiro[[2]benzofuran‐1,1′‐cyclohexan]‐4′‐yl)acetamide (*trans*‐29)


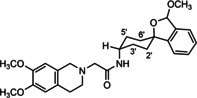


A solution of chloroacetamide *trans*‐**20** (40 mg, 0.13 mmol), isoquinoline **14**⋅HCl (33 mg, 0.14 mmol, 1.1 equiv), Et_3_N (0.05 mL, 0.36 mmol, 2.8 equiv.) and TBAI (48 mg, 0.13 mmol, 1.0 equiv) in DMF (4 mL) was stirred at RT for 66 h. H_2_O (80 mL) was added and the aqueous layer was extracted with CH_2_Cl_2_ (3×60 mL). The combined organic layers were dried (Na_2_SO_4_), filtered, concentrated in vacuo and the residue was purified twice by fc (*d*=2 cm, *l*=20 cm, *V*=10 mL, cyclohexane/ethyl acetate 50 : 50+1 % *N*,*N*‐dimethylethanamine; *d*=2 cm, *l*=18 cm, *V*=10 mL, cyclohexane/ ethyl acetate 50 : 50+1 % *N*,*N*‐dimethylethanamine). Colorless solid, m.p. 107 °C, yield 40 mg (66 %). C_27_H_34_N_2_O_5_ (466.6 g/mol). *R*_f_=0.11 (cyclohexane/ethyl acetate 50 : 50+1 % *N*,*N*‐dimethylethanamine). HRMS (ESI): *m/z* 467.2549 (calcd. 467.2540 for C_27_H_35_N_2_O_5_ [*M*H^+^]). ^1^H NMR (600 MHz, CD_3_OD): *δ*=1.55–1.61 (m, 1H, 2′‐*H*), 1.71–1.77 (m, 1H, 6′‐*H*), 1.79–1.90 (m, 4H, 2′‐*H*, 3′‐*H*, 5′‐*H*, 6′‐*H*), 2.06–2.16 (m, 2H, 3′‐*H*, 5′‐*H*), 2.92 (t, *J*=5.9 Hz, 2H, 3‐*H*_isoquinoline_), 2.97 (t, *J*=5.9 Hz, 2H, 4‐*H*_isoquinoline_), 3.29 (s, 2H, COC*H*_2_N), 3.47 (s, 3H, 3‐OC*H*_3_), 3.70 (s, 2H, 1‐*H*_isoquinoline_), 3.74 (s, 3H, 7‐OC*H*_3_), 3.85 (s, 3H, 6‐OC*H*_3_), 4.17 (quint, *J*=3.7 Hz, 1H, 4′‐*H*_equ_), 6.03 (s, 1H, 3‐*H*), 6.68 (s, 1H, 8‐*H*_isoquinoline_), 6.75 (d, *J*=7.5 Hz, 1H, 7‐*H*), 6.80 (s, 1H, 5‐*H*_isoquinoline_), 7.24 (t, *J*=7.4 Hz, 1H, 6‐*H*), 7.29–7.36 ppm (m, 2H, 4‐*H*, 5‐*H*). A signal for the NH proton is not observed in the spectrum. ^13^C NMR (151 MHz, CD_3_OD): *δ*=27.4 (1 C, *C*‐3′ or *C*‐5′), 27.7 (1 C, *C*‐3′ or *C*‐5′), 30.1 (1 C, *C*‐4_isoquinoline_), 33.8 (1 C, *C*‐2′), 35.1 (1 C, *C*‐6′), 45.1 (1 C, *C*‐4′), 52.6 (1 C, *C*‐3_isoquinoline_), 55.0 (1 C, 3‐O*C*H_3_), 56.4 (1 C, 7‐O*C*H_3_), 56.5 (1 C, 6‐O*C*H_3_), 56.7 (1 C, *C*‐1_isoquinoline_), 62.1 (1 C, CO*C*H_2_N), 87.0 (1 C, *C*‐1), 107.1 (1 C, *C*‐3), 111.1 (1 C, *C*‐8_isoquinoline_), 113.1 (1 C, *C*‐5_isoquinoline_), 121.8 (1 C, *C*‐7), 124.2 (1 C, *C*‐4), 127.2 (1 C, *C*‐4a_isoquinoline_), 127.5 (1 C, *C*‐8a_isoquinoline_), 129.0 (1 C, *C*‐5), 130.4 (1 C, *C*‐6), 138.7 (1 C, *C*‐3a), 148.2 (1 C, *C*‐7a), 149.0 (1 C, *C*‐7_isoquinoline_), 149.4 (1 C, *C*‐6_isoquinoline_), 172.1 ppm (1 C, *C*=O). FTIR (neat): *ν* [cm^−1^]=3341 (N−H), 2932, 2832 (C−H_alkyl_), 1674 (C=O), 1516, 1463, 1451 (C=C_arom_). Purity (HPLC): 92.4 %, *t*_R_=14.0 min.

### *cis*‐2‐(6,7‐Dimethoxy‐3,4‐dihydroisoquinolin‐2(1*H*)‐yl)‐*N*‐(3‐methoxy‐3*H*‐spiro[[2]benzofuran‐1,1′‐cyclohexan]‐4′‐yl)acetamide (*cis*‐29)


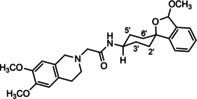


A solution of chloroacetamide *cis*‐**20** (35 mg, 0.11 mmol), isoquinoline **14**⋅HCl (30 mg, 0.13 mmol, 1.2 equiv), Et_3_N (0.05 mL, 0.36 mmol, 3.3 equiv) and TBAI (4 mg, 0.01 mmol, 0.1 equiv) in DMF (4 mL) was stirred at RT for 6 d. H_2_O (80 mL) was added and the aqueous layer was extracted with CH_2_Cl_2_ (3×60 mL). The combined organic layers were dried (Na_2_SO_4_), filtered, concentrated in vacuo and the residue was purified twice by fc (*d*=2 cm, *l*=29 cm, *V*=10 mL, CH_2_Cl_2_/CH_3_OH 99 : 1+1 % *N*,*N*‐dimethylethanamine; *d*=2 cm, *l*=31 cm, *V*=10 mL, CH_2_Cl_2_/CH_3_OH 199 : 1+1 % *N*,*N*‐dimethylethanamine). Yellow oil, yield 45 mg (86 %). C_27_H_34_N_2_O_5_ (466.6 g/mol). *R*_f_=0.31 (CH_2_Cl_2_/CH_3_OH 95 : 5+1 % *N*,*N*‐dimethylethanamine). HRMS (ESI): *m/z* 467.2531 (calcd. 467.2540 for C_27_H_35_N_2_O_5_ [*M*H^+^]). ^1^H NMR (400 MHz, CD_3_OD): *δ*=1.70 (dq, *J*=13.3/2.9 Hz, 1H, 2′‐*H*_equ_), 1.78–1.97 (m, 6H, 3′‐*H*, 5′‐*H*, 6′‐*H*), 2.01 (td, *J*=13.4/4.1 Hz, 1H, 2′‐*H*_ax_), 2.81–2.86 (m, 2H, 3‐*H*_isoquinoline_), 2.87–2.92 (m, 2H, 4‐*H*_isoquinoline_), 3.24 (s, 2H, COC*H*_2_N), 3.49 (s, 3H, 3‐OC*H*_3_), 3.69 (s, 2H, 1‐*H*_isoquinoline_), 3.81 (s, 3H, 7‐OC*H*_3_), 3.83 (s, 3H, 6‐OC*H*_3_), 3.92 (tt, *J*=11.4/4.1 Hz, 1H, 4′‐*H*_ax_), 6.07 (s, 1H, 3‐*H*), 6.67 (s, 1H, 8‐*H*_isoquinoline_), 6.74 (s, 1H, 5‐*H*_isoquinoline_), 7.28 (d, *J*=7.5 Hz, 1H, 7‐*H*), 7.32–7.45 ppm (m, 3H, 4‐*H*, 5‐*H*, 6‐*H*). A signal for the NH proton is not observed in the spectrum. ^13^C NMR (101 MHz, CD_3_OD): *δ*=29.4 (1 C, *C*‐4_isoquinoline_), 29.6 (1 C, *C*‐3′), 30.0 (1 C, *C*‐5′), 37.5 (1 C, *C*‐2′), 38.8 (1 C, *C*‐6′), 48.8 (1 C, *C*‐4′), 52.3 (1 C, *C*‐3_isoquinoline_), 54.9 (1 C, 3‐O*C*H_3_), 56.4 (1 C, *C*‐1_isoquinoline_), 56.48 (1 C, 6‐O*C*H_3_ or 7‐O*C*H_3_), 56.52 (1 C, 6‐O*C*H_3_ or 7‐O*C*H_3_), 62.0 (1 C, CO*C*H_2_N), 86.9 (1 C, *C*‐1), 107.2 (1 C, *C*‐3), 111.1 (1 C, *C*‐8_isoquinoline_), 113.2 (1 C, *C*‐5_isoquinoline_), 121.8 (1 C, *C*‐7), 124.2 (1 C, *C*‐4), 127.2 (1 C, *C*‐4a_isoquinoline_), 127.5 (1 C, *C*‐8a_isoquinoline_), 129.0 (1 C, *C*‐5), 130.5 (1 C, *C*‐6), 138.6 (1 C, *C*‐3a), 148.3 (1 C, *C*‐7a), 148.9 (1 C, *C*‐7_isoquinoline_), 149.2 (1 C, *C*‐6_isoquinoline_), 172.0 ppm (1 C, *C*=O). FTIR (neat): *ν* [cm^−1^]=3333 (N−H), 2932, 2909, 2832 (C−H_alkyl_), 1667 (C=O), 1516, 1439 (C=C_arom_). Purity (HPLC): 88.0 %, *t*_R_=14.4 min.

### 1′‐[2‐(6,7‐Dimethoxy‐3,4‐dihydroisoquinolin‐2(1*H*)‐yl)ethyl]‐3‐methoxy‐3,4‐dihydrospiro[[2]benzopyran‐1,4′‐piperidine] (30)


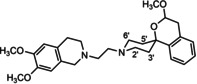


LiAlH_4_ (7 mg, 0.19 mmol, 6.4 equiv) was added to a solution of amide **25** (16 mg, 0.03 mmol) in THF (3 mL) under N_2_. The mixture was heated to reflux for 2 h. After cooling to RT, H_2_O (10 mL) was added, the precipitate was filtered off, and the aqueous phase was extracted with CH_2_Cl_2_ (3×10 mL). The combined organic layers were dried (Na_2_SO_4_), filtered, concentrated in vacuo and the residue was purified twice by fc (*d*=1 cm, *l*=21 cm, *V*=3 mL, CH_2_Cl_2_/CH_3_OH 97 : 3+1 % *N*,*N*‐dimethylethanamine; *d*=1 cm, *l*=21 cm, *V*=3 mL, CH_2_Cl_2_/CH_3_OH 99 : 1+1 % *N*,*N*‐dimethylethanamine). Yellow oil, yield 10 mg (63 %). C_27_H_36_N_2_O_4_ (452.6 g/mol). *R*_f_=0.24 (CH_2_Cl_2_/CH_3_OH 95 : 5+1 % *N*,*N*‐dimethylethanamine). HRMS (ESI): *m/z* 453.2754 (calcd. 453.2748 for C_27_H_37_N_2_O_4_ [*M*H^+^]). ^1^H NMR (600 MHz, CD_3_OD): *δ*=1.82 (dq, *J*=13.8/2.6 Hz, 1H, 3′‐*H*_equ_), 2.00 (ddd, *J*=14.2/12.5/4.3 Hz, 1H, 5′‐*H*_ax_), 2.06 (dq, *J*=14.3/2.8 Hz, 1H, 5′‐*H*_equ_), 2.26 (td, *J*=13.4/4.3 Hz, 1H, 3′‐*H*_ax_), 2.64–2.74 (m, 2H, 2′‐*H*, 6′‐*H*), 2.75–2.86 (m, 7H, 4‐*H*, 3‐*H*_isoquinoline_, NC*H*_2_C*H*_2_N), 2.88 (t, *J*=5.7 Hz, 2H, 4‐*H*_isoquinoline_), 2.91–2.97 (m, 3H, 4‐*H*, 2′‐*H*, 6′‐*H*), 3.55 (s, 3H, 3‐OC*H*_3_), 3.67 (s, 2H, 1‐*H*_isoquinoline_), 3.80 (s, 3H, 7‐OC*H*_3_), 3.81 (s, 3H, 6‐OC*H*_3_), 4.94 (dd, *J*=7.2/3.2 Hz, 1H, 3‐*H*), 6.68 (s, 1H, 8‐*H*_isoquinoline_), 6.71 (s, 1H, 5‐*H*_isoquinoline_), 7.10 (d, *J*=7.5 Hz, 1H, 5‐*H*), 7.18 (ddd, *J*=7.6/6.2/2.4 Hz, 1H, 6‐*H*), 7.19–7.24 ppm (m, 2H, 7‐*H*, 8‐*H*). ^13^C NMR (151 MHz, CD_3_OD): *δ*=29.1 (1 C, *C*‐4_isoquinoline_), 36.1 (1 C, *C*‐4), 37.1 (1 C, *C*‐5′), 39.5 (1 C, *C*‐3′), 50.9 (1 C, *C*‐2′), 51.0 (1 C, *C*‐6′), 52.5 (1 C, *C*‐3_isoquinoline_), 56.1 (1 C, N_isoquinoline_ *C*H_2_CH_2_N), 56.46 (2 C, 3‐O*C*H_3_, 6‐O*C*H_3_ or 7‐O*C*H_3_), 56.51 (1 C, N_isoquinoline_CH_2_ *C*H_2_N), 56.7 (1 C, 6‐O*C*H_3_ or 7‐O*C*H_3_), 56.9 (1 C, *C*‐1_isoquinoline_), 75.6 (1 C, *C*‐1), 97.9 (1 C, *C*‐3), 111.2 (1 C, *C*‐8_isoquinoline_), 113.0 (1 C, *C*‐5_isoquinoline_), 125.7 (1 C, *C*‐8), 127.3 (1 C, *C*‐8a_isoquinoline_), 127.4 (1 C, *C*‐4a_isoquinoline_), 127.6 (1 C, *C*‐7), 127.9 (1 C, *C*‐6), 130.2 (1 C, *C*‐5), 132.7 (1 C, *C*‐4a), 141.9 (1 C, *C*‐8a), 148.9 (1 C, *C*‐7_isoquinoline_), 149.3 ppm (1 C, *C*‐6_isoquinoline_). FTIR (neat): *ν* [cm^−1^]=2947, 2820, 2778 (C−H_alkyl_), 1516, 1466, 1454 (C=C_arom_). Purity (HPLC): 91.2 %, *t*_R_=14.0 min.

### *trans*‐*N*‐[2‐(6,7‐Dimethoxy‐3,4‐dihydroisoquinolin‐2(1*H*)‐yl)ethyl]‐3‐methoxy‐3,4‐dihydrospiro[[2]benzopyran‐1,1′‐cyclohexan]‐4′‐amine (*trans*‐31)


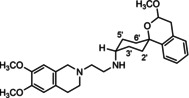


LiAlH_4_ (4 mg, 0.12 mmol, 5.3 equiv) was added to a solution of amide *trans*‐**26** (11 mg, 0.02 mmol) in THF (3 mL) under N_2_. The mixture was heated to reflux for 22 h. After cooling to RT, H_2_O (10 mL) was added, the precipitate was filtered off, and the aqueous phase was extracted with CH_2_Cl_2_ (5×10 mL). The combined organic layers were dried (Na_2_SO_4_), filtered, concentrated in vacuo and the residue was purified twice by fc (*d*=1 cm, *l*=25 cm, *V*=3 mL, CH_2_Cl_2_/CH_3_OH 99 : 1+1 % *N*,*N*‐dimethylethanamine; *d*=1 cm, *l*=25 cm, *V*=3 mL, CH_2_Cl_2_/CH_3_OH 99 : 1→97 : 3+1 % *N*,*N*‐dimethylethanamine). Yellow solid, m.p. 73 °C, yield 9 mg (86 %). C_28_H_38_N_2_O_4_ (466.6 g/mol). *R*_f_=0.33 (CH_2_Cl_2_/CH_3_OH 90 : 10+1 % *N*,*N*‐dimethylethanamine). HRMS (APCI): *m/z* 467.2895 (calcd. 467.2904 for C_28_H_39_N_2_O_4_ [*M*H^+^]). ^1^H NMR (600 MHz, CD_3_OD): *δ*=1.60 (dt, *J*=9.5/2.0 Hz, 1H, 2′‐*H*), 1.78–1.83 (m, 2H, 3′‐*H*, 5′‐*H*), 1.84–1.89 (m, 2H, 6′‐*H*), 2.06–2.13 (m, 1H, 5′‐*H*), 2.13–2.17 (m, 2H, 2′‐*H*, 3′‐*H*), 2.73–2.85 (m, 5H, 4‐*H*, N_isoquinoline_C*H*_2_CH_2_N, 3‐*H*_isoquinoline_), 2.86–2.95 (m, 5H, 4‐*H*, N_isoquinoline_CH_2_C*H*_2_N, 4‐*H*_isoquinoline_), 3.02 (quint, *J*=3.0 Hz, 1H, 4′‐*H*_equ_), 3.55 (s, 3H, 3‐OCH_3_), 3.66 (s, 2H, 1‐*H*_isoquinoline_), 3.78 (s, 3H, 7‐OC*H*_3_), 3.82 (s, 3H, 6‐OC*H*_3_), 4.90 (dd, *J*=7.5/3.1 Hz, 1H, 3‐*H*), 6.68 (s, 1H, 8‐*H*_isoquinoline_), 6.73 (s, 1H, 5‐*H*_isoquinoline_), 7.01–7.08 (m, 2H, 5‐*H*, 7‐*H*), 7.09–7.13 (m, 1H, 6‐*H*), 7.17 ppm (d, *J*=7.8 Hz, 1H, 8‐*H*). A signal for the NH proton is not observed in the spectrum. ^13^C NMR (151 MHz, CD_3_OD): *δ*=25.9 (1 C, *C*‐3′), 26.2 (1 C, *C*‐5′), 29.5 (1 C, *C*‐4_isoquinoline_), 31.5 (1 C, *C*‐6′), 34.1 (1 C, *C*‐2′), 36.2 (1 C, *C*‐4), 44.7 (1 C, N_isoquinoline_CH_2_ *C*H_2_N), 52.5 (1 C, *C*‐4′), 52.5 (1 C, *C*‐3_isoquinoline_ or N_isoquinoline_ *C*H_2_CH_2_N), 56.3 (1 C, 3‐O*C*H_3_), 56.4–56.5 (m, 2 C, 6‐O*C*H_3_, 7‐O*C*H_3_), 56.8 (1 C, *C*‐1_isoquinoline_), 58.0 (1 C, *C*‐3_isoquinoline_ or N_isoquinoline_ *C*H_2_CH_2_N), 77.8 (1 C, *C*‐1), 97.9 (1 C, *C*‐3), 111.3 (1 C, *C*‐8_isoquinoline_), 113.1 (1 C, *C*‐5_isoquinoline_), 125.9 (1 C, *C*‐8), 127.4 (1 C, *C*‐4a_isoquinoline_), 127.5 (1 C, *C*‐7), 127.55 (1 C, *C*‐6), 127.62 (1 C, *C*‐8a_isoquinoline_), 130.0 (1 C, *C*‐5), 132.4 (1 C, *C*‐4a), 143.2 (1 C, *C*‐8a), 148.9 (1 C, *C*‐7_isoquinoline_), 149.3 ppm (1 C, *C*‐6_isoquinoline_). FTIR (neat): *ν* [cm^−1^]=3368 (N−H), 2924, 2832 (C−H_alkyl_), 1516, 1447 (C=C_arom_). Purity (HPLC): 61.5 %, *t*_R_=14.7 min.

### *cis*‐*N*‐[2‐(6,7‐Dimethoxy‐3,4‐dihydroisoquinolin‐2(1*H*)‐yl)ethyl]‐3‐methoxy‐3,4‐dihydrospiro[[2]benzopyran‐1,1′‐cyclohexan]‐4′‐amine (*cis*‐31)


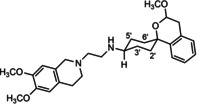


LiAlH_4_ (8 mg, 0.22 mmol, 6.0 equiv.) was added to a solution of amide *cis*‐**26** (17 mg, 0.04 mmol) in THF (3 mL) under N_2_. The mixture was heated to reflux for 19 h. After cooling to RT, H_2_O (10 mL) was added, the precipitate was filtered off, and the aqueous phase was extracted with CH_2_Cl_2_ (4×10 mL). The combined organic layers were dried (Na_2_SO_4_), filtered, concentrated in vacuo and the residue was purified by fc (*d*=1 cm, *l*=25 cm, *V*=3 mL, CH_2_Cl_2_/CH_3_OH 98 : 2→98 : 2+1 % *N*,*N*‐dimethylethanamine). Yellow solid, m.p. 70 °C, yield 13 mg (76 %). C_28_H_38_N_2_O_4_ (466.6 g/mol). *R*_f_=0.26 (CH_2_Cl_2_/CH_3_OH 90 : 10+1 % *N*,*N*‐dimethylethanamine). HRMS (APCI): *m/z* 467.2921 (calcd. 467.2904 for C_28_H_39_N_2_O_4_ [*M*H^+^]). ^1^H NMR (400 MHz, CD_3_OD): *δ*=1.65–1.82 (m, 3H, 2′‐*H*, 3′‐*H*, 5′‐*H*), 1.82–1.96 (m, 3H, 3′‐*H*, 5′‐*H*, 6′‐*H*), 2.03 (td, *J*=13.5/4.2 Hz, 1H, 6′‐*H*), 2.10–2.18 (m, 1H, 2′‐*H*), 2.71–2.78 (m, 3H, 4′‐*H*_ax_, N_isoquinoline_C*H*_2_CH_2_N), 2.78–2.85 (m, 3H, 4‐*H*, 3‐*H*_isoquinoline_), 2.88 (t, *J*=5.9 Hz, 2H, 4‐*H*_isoquinoline_), 2.91–2.98 (m, 3H, 4‐*H*, N_isoquinoline_CH_2_C*H*_2_N), 3.58 (s, 3H, 3‐OC*H*_3_), 3.64 (s, 2H, 1‐*H*_isoquinoline_), 3.82 (s, 3H, 6‐OC*H*_3_ or 7‐OC*H*_3_), 3.82 (s, 3H, 6‐OC*H*_3_ or 7‐OC*H*_3_), 4.92 (dd, *J*=7.4/3.2 Hz, 1H, 3‐*H*), 6.69 (s, 1H, 8‐*H*_isoquinoline_), 6.73 (s, 1H, 5‐*H*_isoquinoline_), 7.10 (d, *J*=7.2 Hz, 1H, 5‐*H*), 7.14–7.22 ppm (m, 3H, 6‐*H*, 7‐*H*, 8‐*H*). A signal for the NH proton was not observed. ^13^C NMR (101 MHz, CD_3_OD): *δ*=29.0 (1 C, *C*‐3′ or *C*‐5′), 29.1 (1 C, *C*‐3′ or *C*‐5′), 29.2 (1 C, *C*‐4_isoquinoline_), 36.2 (1 C, *C*‐4), 36.5 (1 C, *C*‐2′), 39.0 (1 C, *C*‐6′), 44.3 (1 C, N_isoquinoline_CH_2_ *C*H_2_N), 52.4 (1 C, *C*‐3_isoquinoline_), 56.4 (1 C, 3‐O*C*H_3_), 56.47 (1 C, 6‐O*C*H_3_ or 7‐O*C*H_3_), 56.53 (1 C, 6‐O*C*H_3_ or 7‐O*C*H_3_), 56.8 (1 C, *C*‐1_isoquinoline_), 57.4 (1 C, *C*‐4′), 58.2 (1 C, N_isoquinoline_ *C*H_2_CH_2_N), 77.4 (1 C, *C*‐1), 97.8 (1 C, *C*‐3), 111.2 (1 C, *C*‐8_isoquinoline_), 113.0 (1 C, *C*‐5_isoquinoline_), 125.7 (1 C, *C*‐8), 127.4 (1 C, *C*‐4a_isoquinoline_), 127.50 (1 C, *C*‐7), 127.53 (1 C, *C*‐8a_isoquinoline_), 127.7 (1 C, *C*‐6), 130.1 (1 C, *C*‐5), 132.6 (1 C, *C*‐4a), 142.6 (1 C, *C*‐8a), 148.9 (1 C, *C*‐7_isoquinoline_), 149.3 ppm (1 C, *C*‐6_isoquinoline_). FTIR (neat): *ν* [cm^−1^]=3402 (N−H), 2928, 2832 (C−H_alkyl_), 1516, 1447 (C=C_arom_). Purity (HPLC): 69.2 %, *t*_R_=14.5 min.

### Receptor binding studies

The σ_1_ and σ_2_ affinities were recorded as described in ref. [40]. Details of the assays are given in the Supporting Information.

## Conflict of interest

The authors declare no conflict of interest.

## Supporting information

As a service to our authors and readers, this journal provides supporting information supplied by the authors. Such materials are peer reviewed and may be re‐organized for online delivery, but are not copy‐edited or typeset. Technical support issues arising from supporting information (other than missing files) should be addressed to the authors.

Supplementary
