# Supplementary material for: Synthesis of σ Receptor Ligands with a Spirocyclic System Connected with a Tetrahydroisoquinoline Moiety via Different Linkers
Source: ChemMedChem. 2021 Feb 2;16(7):1184–97. doi: 10.1002/cmdc.202000861 (PMC8048568; doi:10.1002/cmdc.202000861)

ChemMedChem

Supporting Information

Synthesis of σ Receptor Ligands with a Spirocyclic System Connected with a Tetrahydroisoquinoline Moiety via Different Linkers

Melanie Bergkemper, Dirk Schepmann, and Bernhard Wünsch*

Table of Contents

Content	page
1. Receptor binding studies	S2
2. References	S5
3. ^{1}H and ^{13}C NMR spectra	S6

1. Receptor binding studies

1.1. Materials

Guinea pig brains and rat livers were commercially available (Harlan-Winkelmann, Borchen, Germany). Homogenizers: Elvehjem Potter (B. Braun Biotech International, Melsungen, Germany) and Soniprep® 150 (MSE, London, UK). Centrifuges: Cooling centrifuge model Eppendorf 5427R (Eppendorf, Hamburg, Germany) and High-speed cooling centrifuge model Sorvall® RC-5C plus (Thermo Fisher Scientific, Langenselbold, Germany). Multiplates: standard 96 well multiplates (Diagonal, Muenster, Germany). Shaker: self-made device with adjustable temperature and tumbling speed (scientific workshop of the institute). Harvester: MicroBeta® FilterMate 96 Harvester. Filter: Printed Filtermat Typ A and B. Scintillator: Meltilex® (Typ A or B) solid state scintillator. Scintillation analyzer: MicroBeta® Trilux (all Perkin Elmer LAS, Rodgau-Jügesheim, Germany).

1.2. Preparation of membrane homogenates from guinea pig brain

5 guinea pig brains were homogenized with the potter (500-800 rpm, 10 up and down strokes) in 6 volumes of cold 0.32 M sucrose. The suspension was centrifuged at 1,200 x g for 10 min at 4 °C. The supernatant was separated and centrifuged at 23,500 x g for 20 min at 4 °C. The pellet was resuspended in 5-6 volumes of buffer (50 mM TRIS, pH 7.4) and centrifuged again at 23,500 x g (20 min, 4 °C). This procedure was repeated twice. The final pellet was resuspended in 5-6 volumes of buffer and frozen (-80 °C) in 1.5 mL portions containing about 1.5 mg protein/mL.

1.3. Preparation of membrane homogenates from rat liver

Two rat livers were cut into small pieces and homogenized with the potter (500-800 rpm, 10 up and down strokes) in 6 volumes of cold 0.32 M sucrose. The suspension was centrifuged at 1,200 x g for 10 min at 4 °C. The supernatant was separated and centrifuged at 31,000 x g for 20 min at 4 °C. The pellet was resuspended in 5-6 volumes of buffer (50 mM TRIS, pH 8.0) and incubated at rt for 30 min. After the incubation, the suspension was centrifuged again at 31,000 x g for 20 min at 4 °C. The final pellet was resuspended in 5-6 volumes of buffer and stored at -80 °C in 1.5 mL portions containing about 2 mg protein/mL.

1.4. Protein determination

The protein concentration was determined by the method of Bradford,[1] modified by Stoscheck.[2] The Bradford solution was prepared by dissolving 5 mg of Coomassie Brilliant Blue G 250 in 2.5 mL of EtOH (95 %, v/v). 10 mL deionized H_2O and 5 mL phosphoric acid (85 %, m/v) were added to this solution, the mixture was stirred and filled to a total volume of 50 mL with deionized water. The calibration was carried out using bovine serum albumin as a standard in 9 concentrations (0.1, 0.2, 0.4, 0.6, 0.8, 1.0, 1.5, 2.0 and 4.0 mg /mL). In a 96 well standard multiplate, 10 µL of the calibration solution or 10 µL of the membrane receptor preparation were mixed with 190 µL of the Bradford solution, respectively. After 5 min, the UV absorption of the protein-dye complex at λ = 595 nm was measured with a plate reader (Tecan Genios®, Tecan, Crailsheim, Germany).

1.1.1 **3.5. General procedures for the receptor binding assays**

The test compound solutions were prepared by dissolving approximately 10 µmol (usually 2-4 mg) of test compound in DMSO so that a 10 mM stock solution was obtained. To obtain the required test solutions for the assay, the DMSO stock solution was diluted with the respective assay buffer. The filtermats were presoaked in 0.5 % aqueous polyethylenimine solution for 2 h at rt before use. All binding experiments were carried out in duplicates in the 96 well multiplates. The concentrations given are the final concentration in the assay. Generally, the assays were performed by addition of 50 µL of the respective assay buffer, 50 µL of test compound solution in various concentrations (10^{-5}, 10^{-6}, 10^{-7}, 10^{-8}, 10^{-9} and 10^{-10} mol/L), 50 µL of the corresponding radioligand solution and 50 µL of the respective receptor preparation into each well of the multiplate (total volume 200 µL). The receptor preparation was always added last. During the incubation, the multiplates were shaken at a speed of 500-600 rpm at the specified temperature. Unless otherwise noted, the assays were terminated after 120 min by rapid filtration using the harvester. During the filtration, each well was washed five times with 300 µL of water. Subsequently, the filtermats were dried at 95 °C. The solid scintillator was melted on the dried filtermats at a temperature of 95 °C for 5 min. After solidifying of the scintillator at rt, the trapped radioactivity in the filtermats was measured with the scintillation analyzer. Each position on the filtermat corresponding to one well of the multiplate was measured for 5 min with the [^{3}H]-counting protocol. The overall counting efficiency was 20 %. The IC_{50} values were

calculated with the program GraphPad Prism® 3.0 (GraphPad Software, San Diego, CA, USA) by non-linear regression analysis. Subsequently, the IC_{50} values were transformed into K_i values using the equation of Cheng and Prusoff.[3] The K_i values are given as mean value ± SEM from three independent experiments.

1.6. σ_1 receptor assay

The assay was performed with the radioligand [^{3}H]-(+)-pentazocine (22.0 Ci/mmol; Perkin Elmer). The thawed membrane preparation of guinea pig brain (about 100 µg of the protein) was incubated with various concentrations of test compounds, 2 nM [^{3}H]-(+)-pentazocine, and TRIS buffer (50 mM, pH 7.4) at 37 °C. The non-specific binding was determined with 10 µM unlabeled (+)-pentazocine. The K_d value of (+)-pentazocine is 2.9 nM.[4]

1.7. σ_2 receptor assay

The assays were performed with the radioligand [^{3}H]di-*o*-tolylguanidine (specific activity 50 Ci/mmol; ARC, St. Louis, MO, USA). The thawed rat liver membrane preparation (about 100 µg protein) was incubated with various concentrations of the test compound, 3 nM [^{3}H]di-*o*-tolylguanidine and buffer containing (+)-pentazocine (500 nM (+)-pentazocine in TRIS buffer (50 mM TRIS, pH 8.0)) at rt. The non-specific binding was determined with 10 µM non-labeled di-*o*-tolylguanidine. The K_d value of di-*o*-tolylguanidine is 17.9 nM.[5]

2. References

1. Bradford, M. M. A rapid and sensitive method for the quantitation of microgram quantities of protein utilizing the principle of protein-dye binding. *Anal. Biochem.* **1976**, *72*, 248–254.
2. Stoscheck, C. Quantification of protein. *Methods Enzymol.* **1990**, *182*, 50–68.
3. Cheng, Y.-C.; Prusoff, W. H. Relationship between the inhibition constant (KI) and the concentration of inhibitor which causes 50 per cent inhibition (I50) of an enzymatic reaction. *Biochem. Pharmacol.*, **1973**, *22*, 3099–3108.
4. DeHaven-Hudkins, D. L.; Fleissner, L. C.; Ford-Rice, F. Y. Characterization of the binding of [^{3}H](+)-pentazocine to σ recognition sites in guinea pig brain. *Eur. J. Pharmacol. Mol. Pharmacol.* **1992**, *227*, 371–378.
5. Mach, R. H.; Smith, C. R.; Childers, S. R. Ibogaine possesses a selective affinity for σ2 receptors. *Life Sci.* **1995**, *57*, PL57–PL62.

3. ^{1}H and ^{13}C NMR spectra

^{1}H- and ^{13}C-NMR-spectrum of *trans*-3-Methoxy-3*H*-spiro[[2]benzofuran-1,1'-cyclohexan]-4'-amine (*trans*-**13**) in CD_3OD.

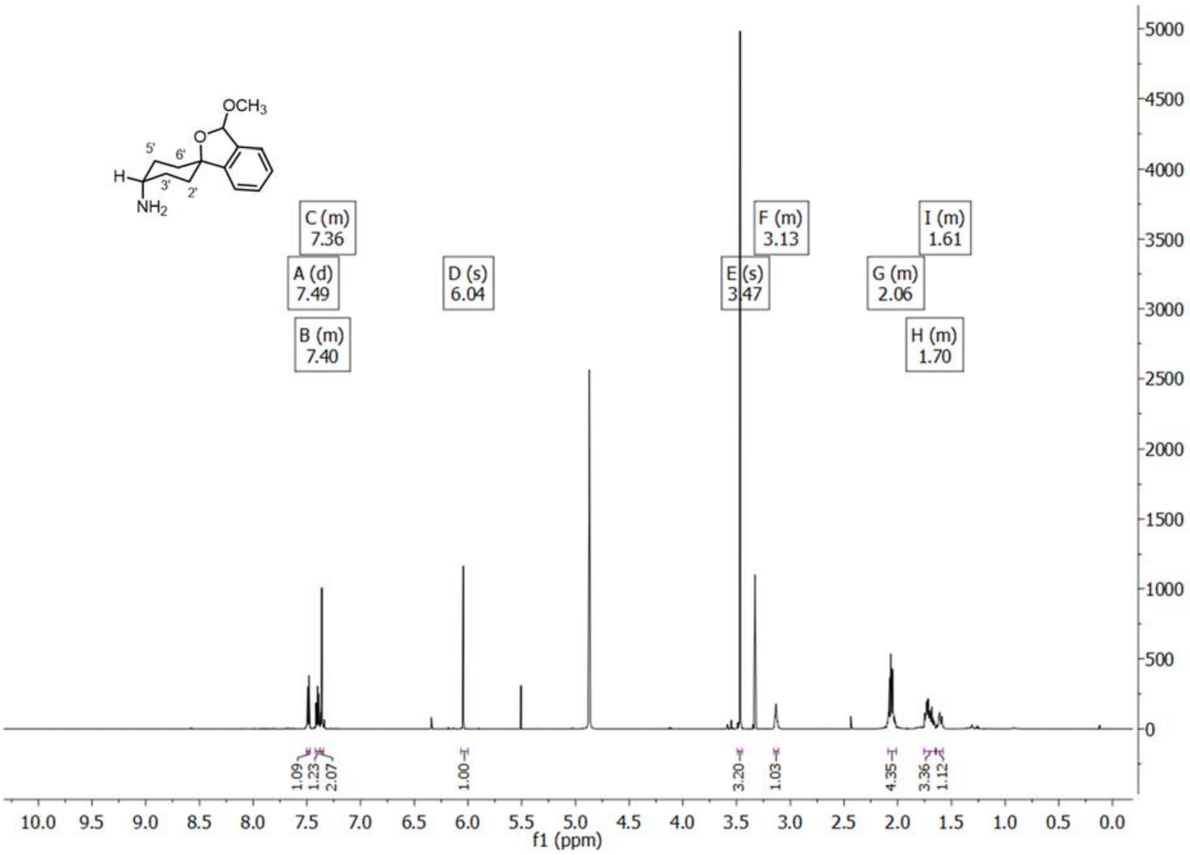

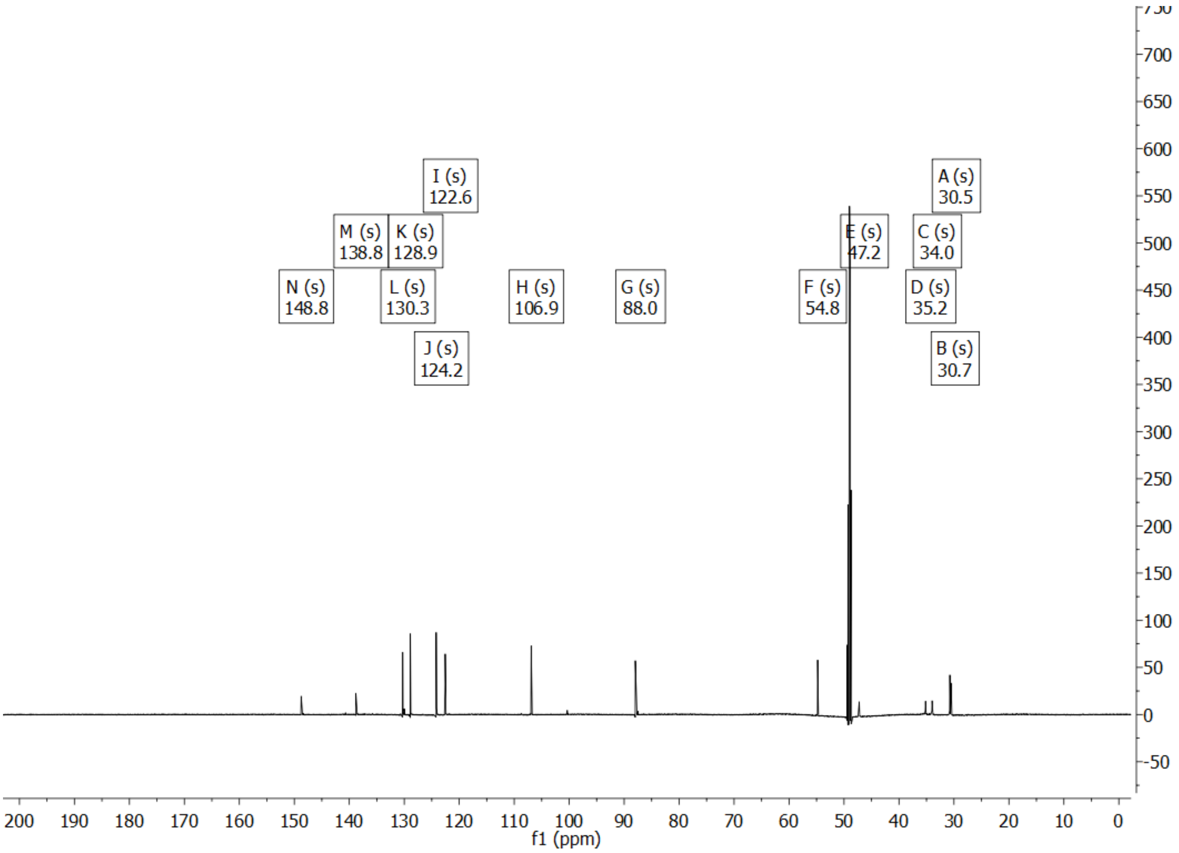

^{1}H- and ^{13}C-NMR-spectrum of *cis*-3-Methoxy-3*H*-spiro[[2]benzofuran-1,1’-cyclohexan]-4’-amine (*ciss*-**13**) in CD_3OD.

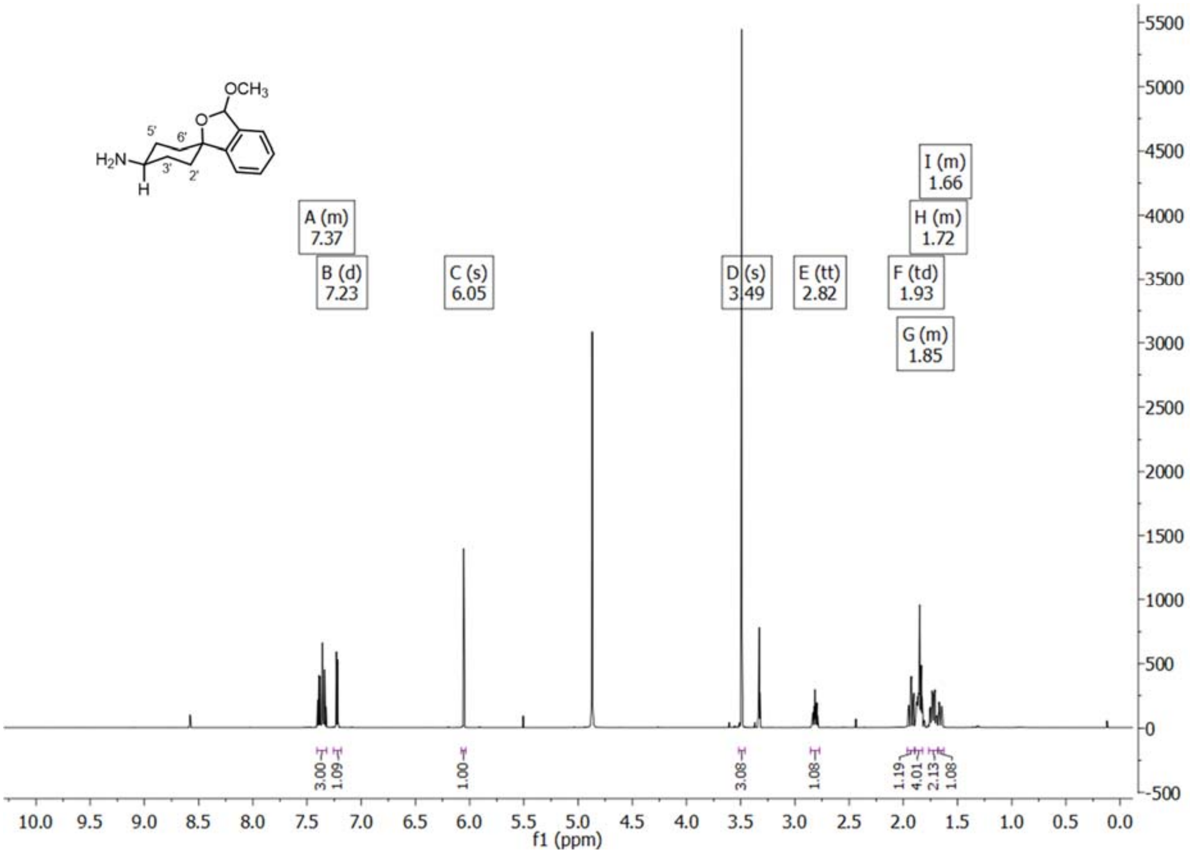

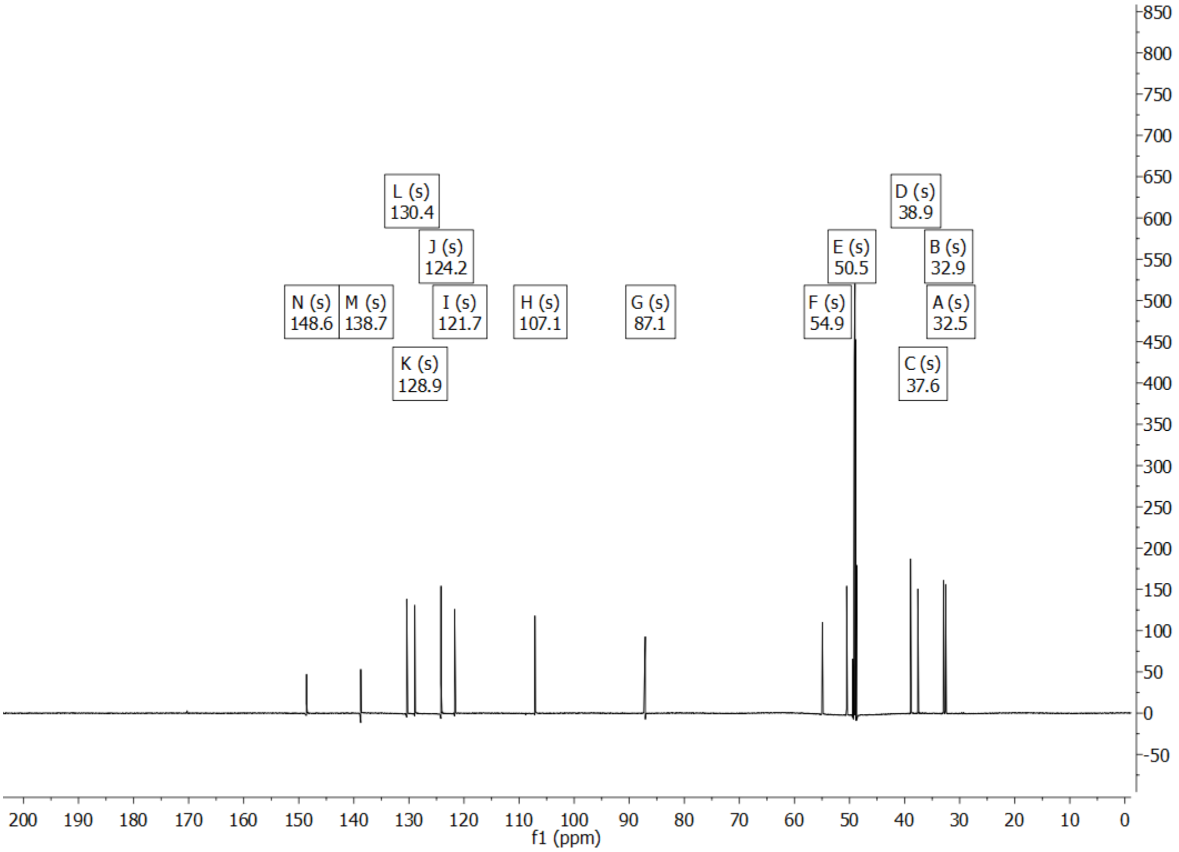

^{1}H- and ^{13}C-NMR-spectrum of 2-Chloro-1-(6,7-dimethoxy-3,4-dihydroisoquinolin-2(1*H*)-yl)ethan-1-one (**15**) in $CDCl_3$.

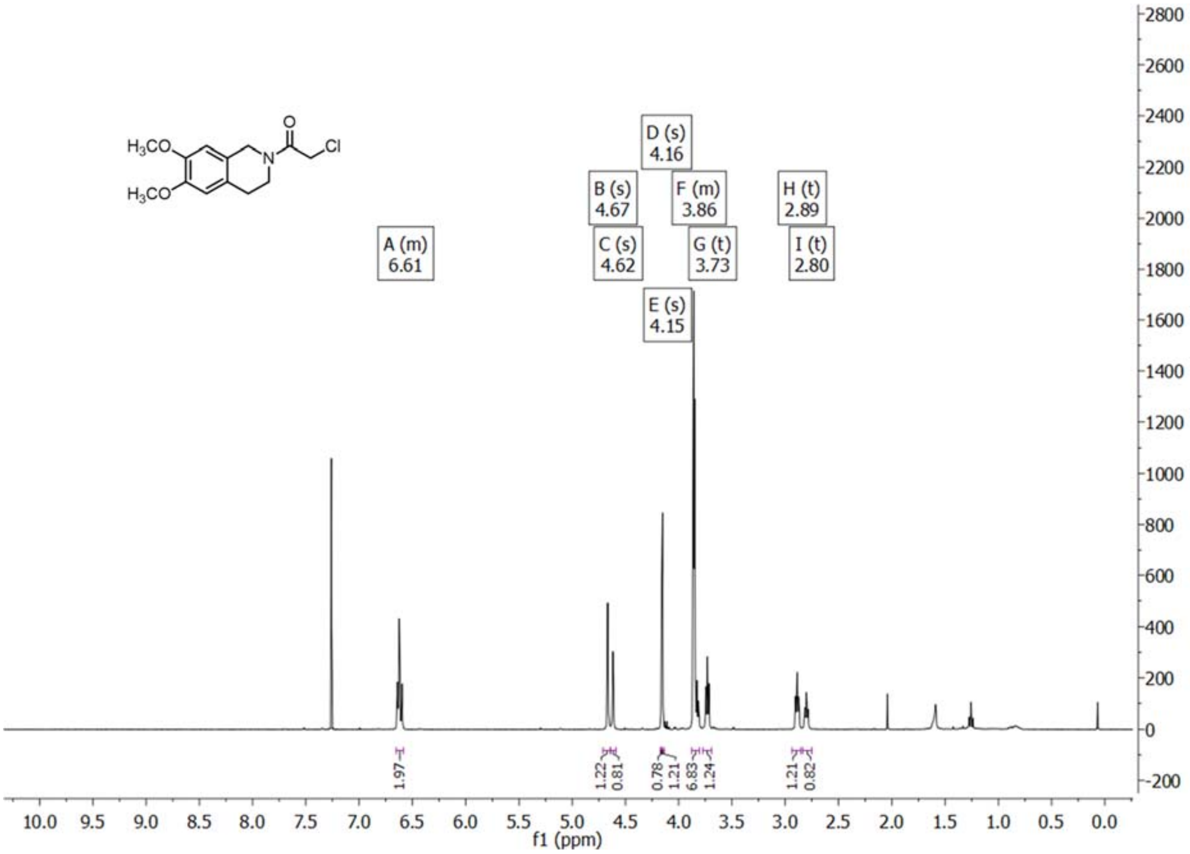

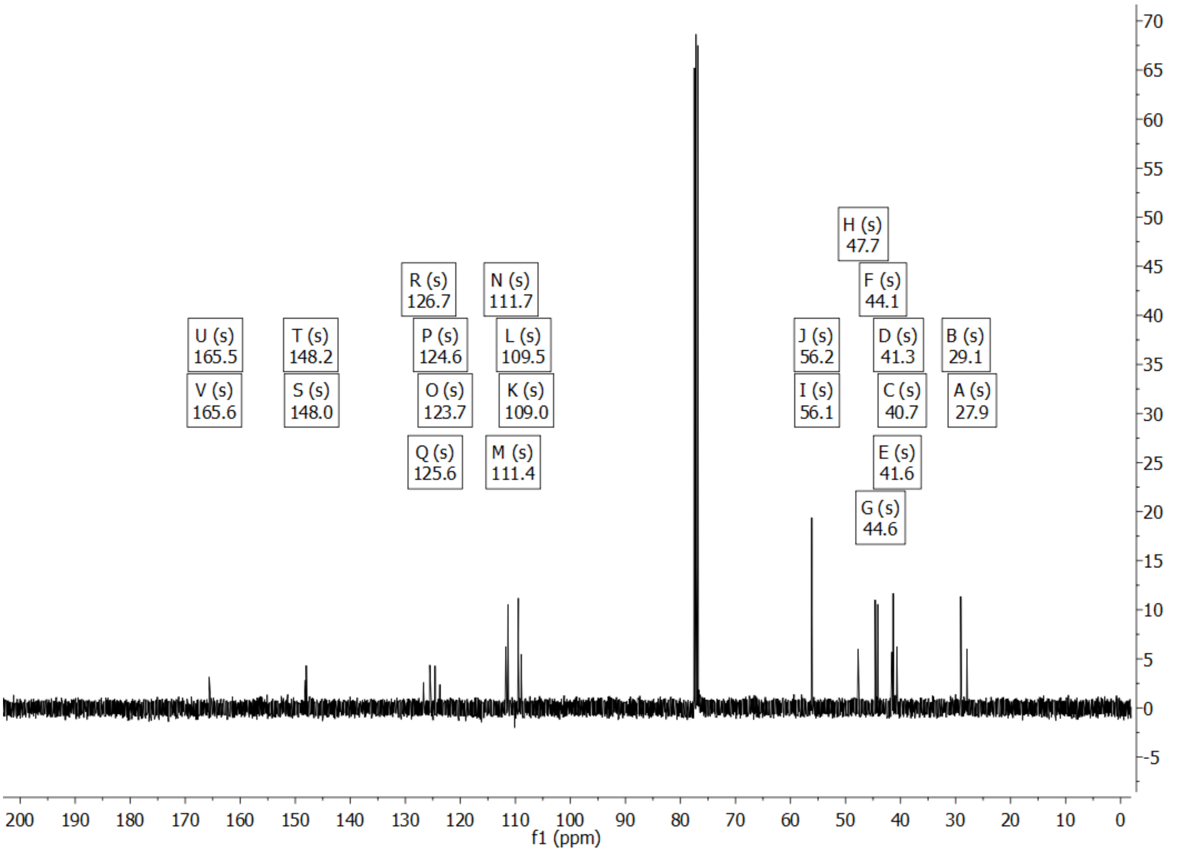

^{1}H- and ^{13}C-NMR-spectrum of *trans*-2-Chloro-N-(3-methoxy-3,4-dihydrospiro[[2]benzopyran-1,1'-cyclohexan]-4'-yl)acetamide (*trans*-**17**) in CD_3OD.

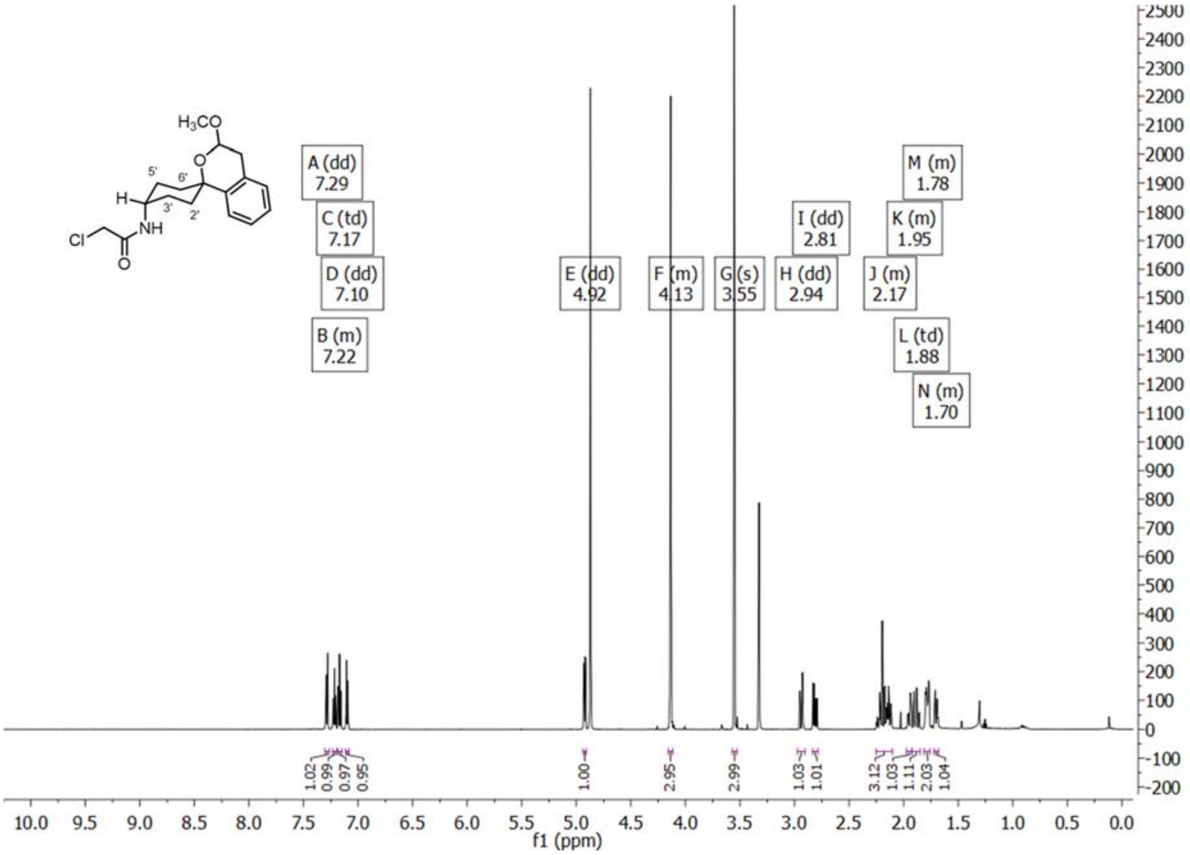

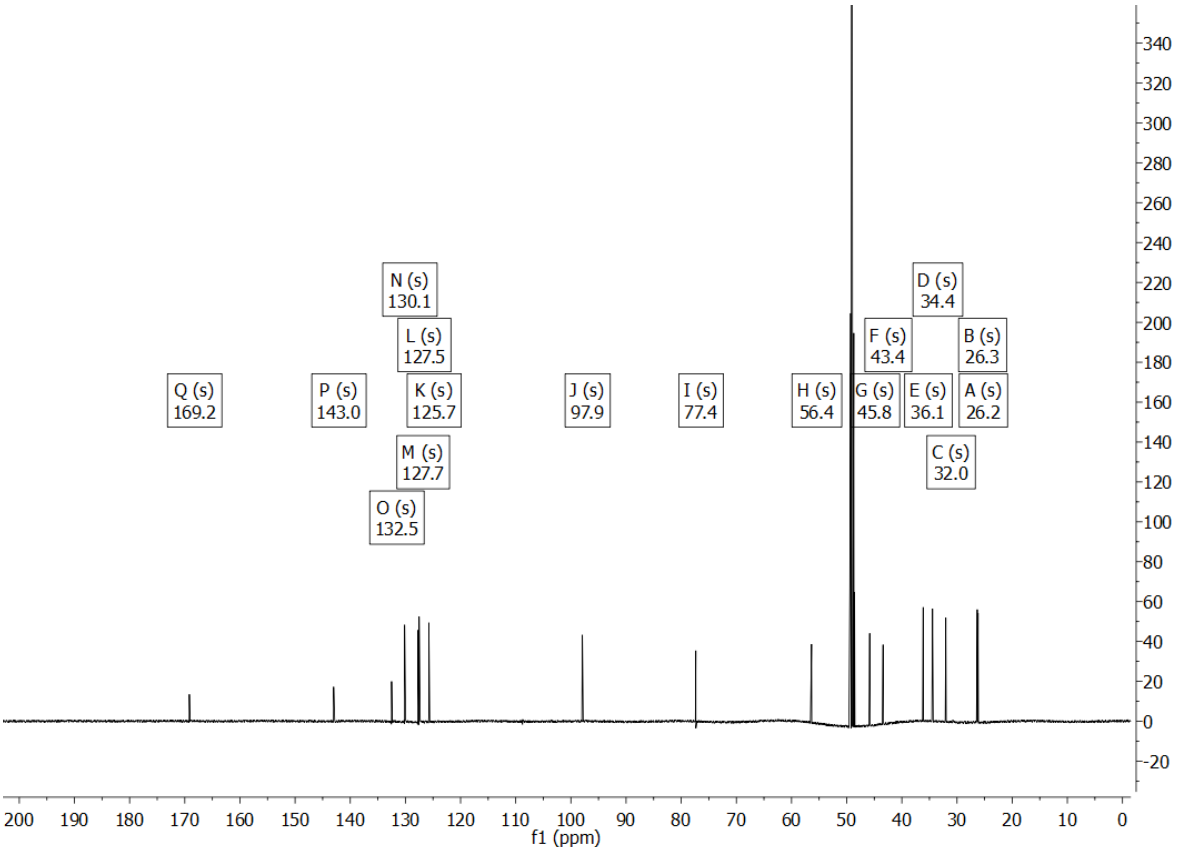

^{1}H- and ^{13}C-NMR-spectrum of *cis*-2-Chloro-N-(3-methoxy-3,4-dihydrospiro[[2]benzopyran-1,1'-cyclohexan]-4'-yl)acetamide (*cis*-**17**) in CD_3OD.

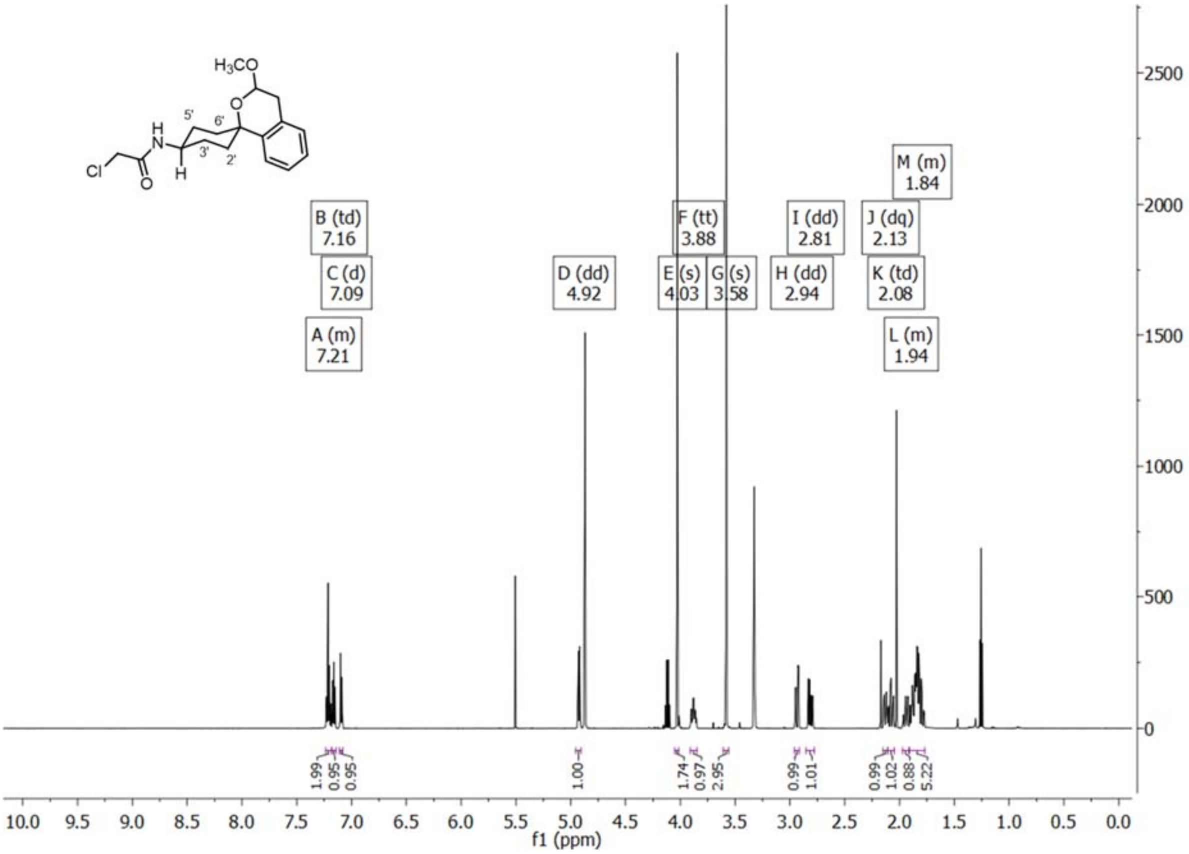

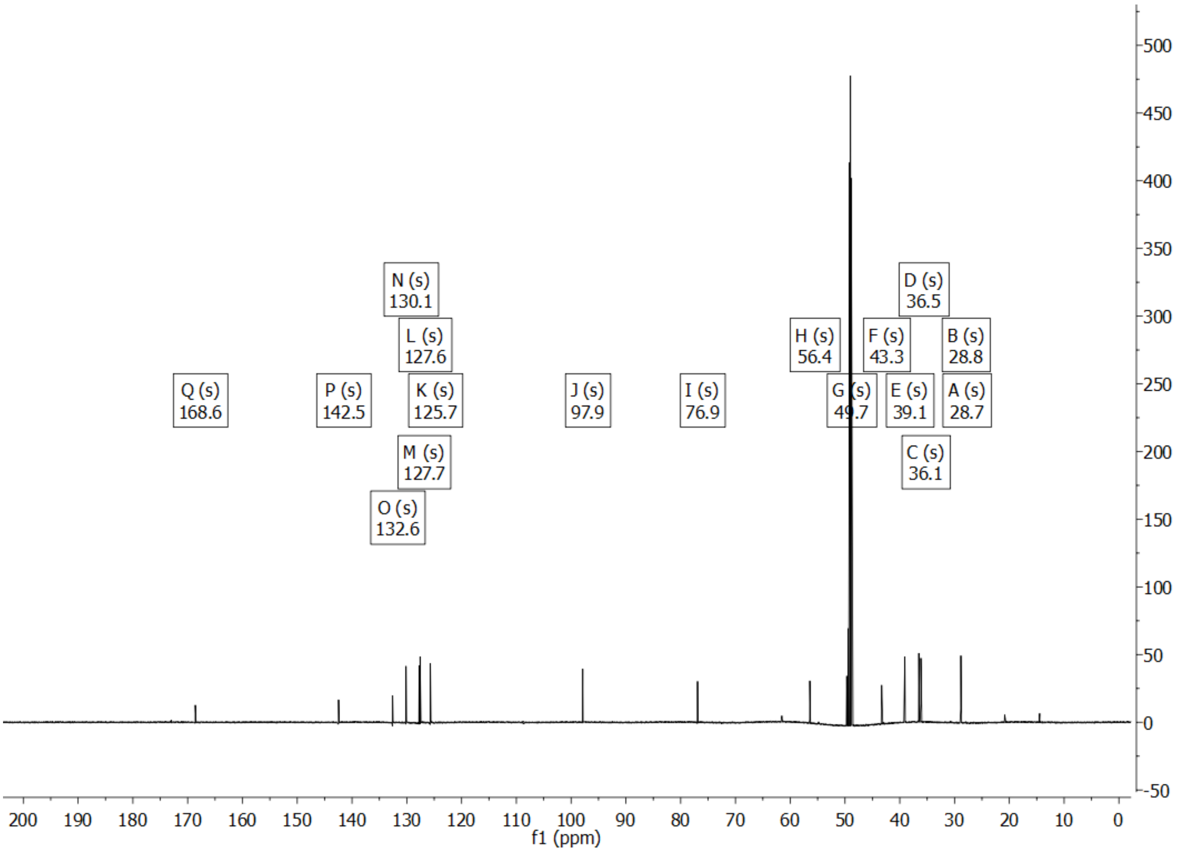

^{1}H- and ^{13}C-NMR-spectrum of *cis*-4-Chloro-N-(3-methoxy-3,4-dihydrospiro[[2]benzopyran-1,1'-cyclohexan]-4'-yl)butanamide (*cis*-**18**) in CD_3OD.

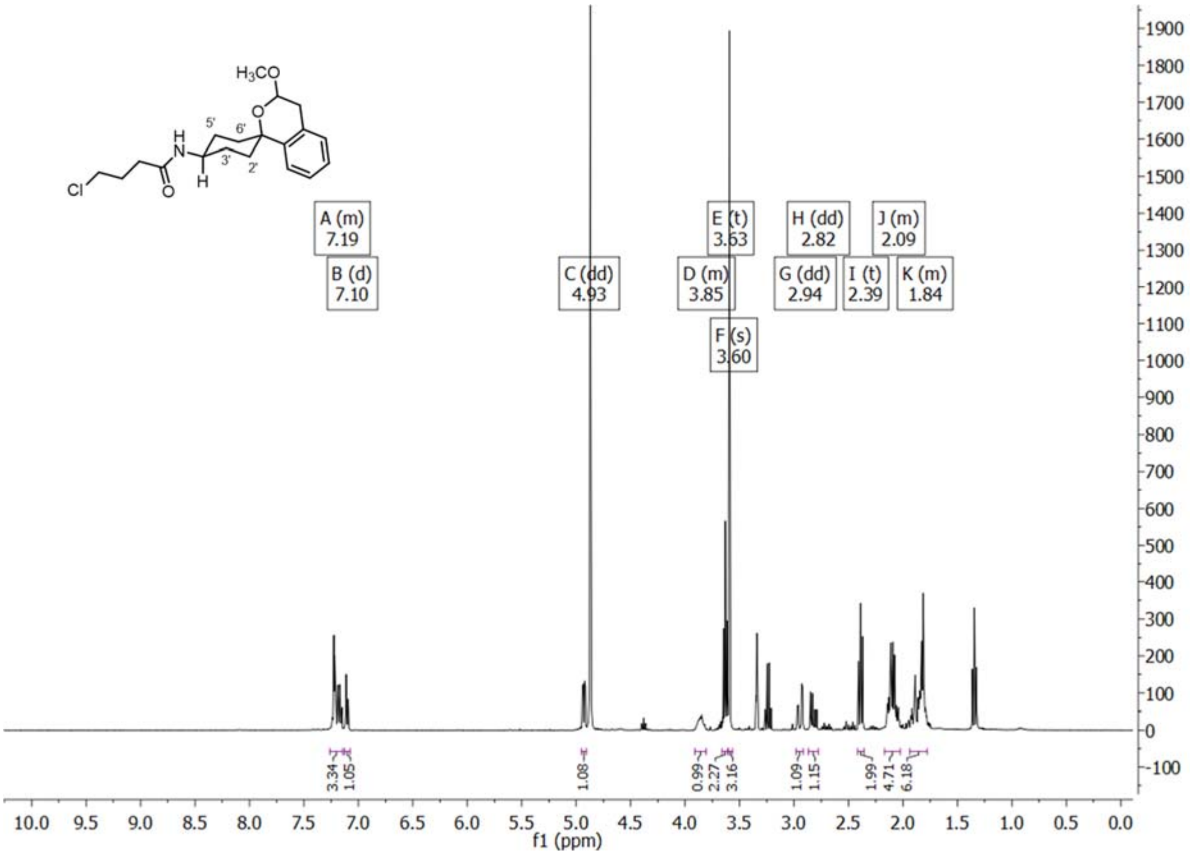

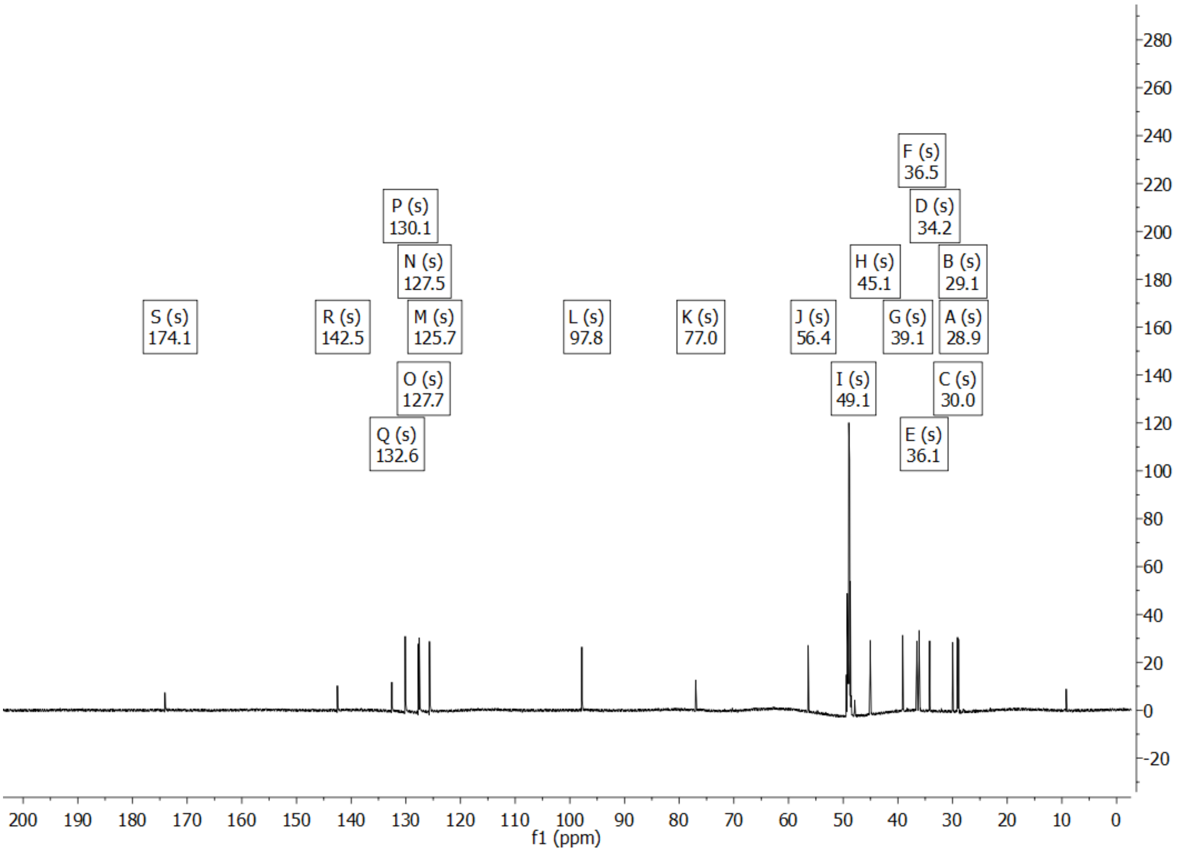

^{1}H- and ^{13}C-NMR-spectrum of *trans*-2-Chloro-N-(3-methoxy-3*H*-spiro[[2]benzopyran-1,1’-cyclohexan]-4’-yl)acetamide (*trans*-**20**) in CD_3OD.

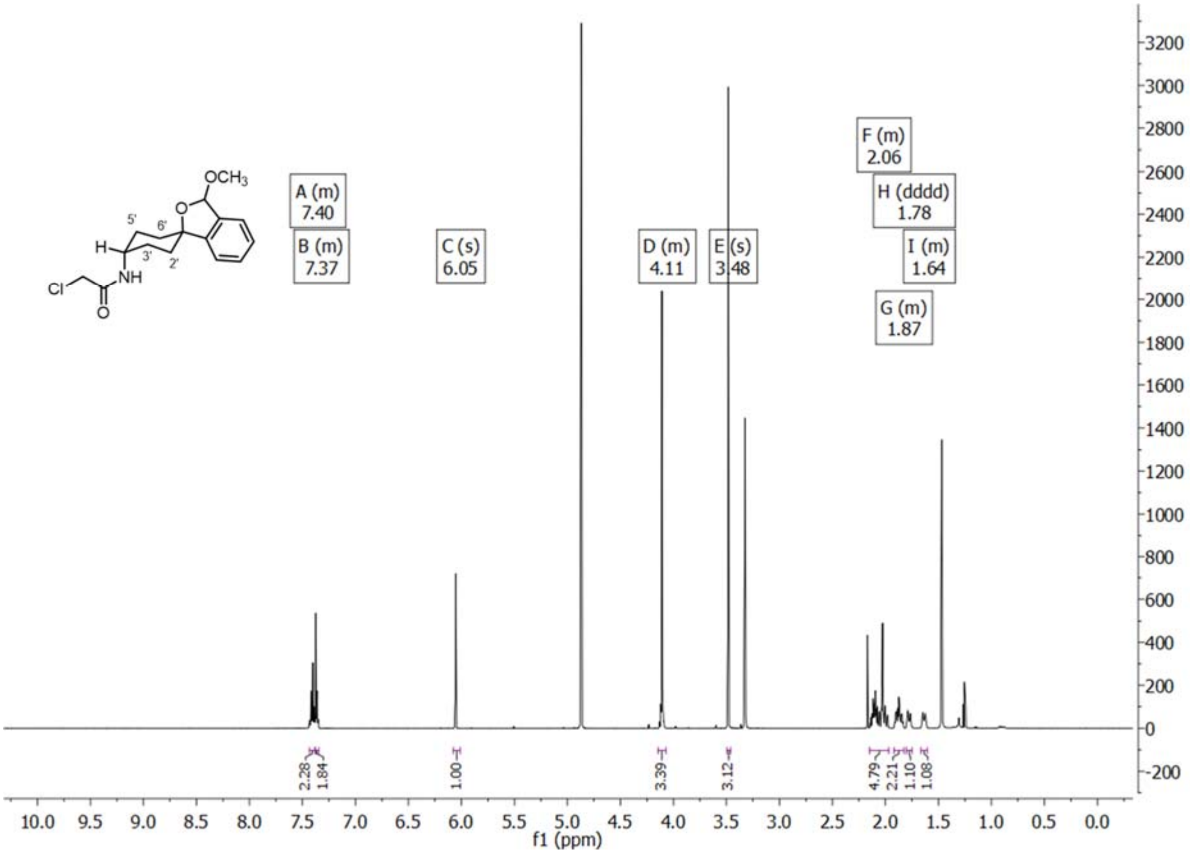

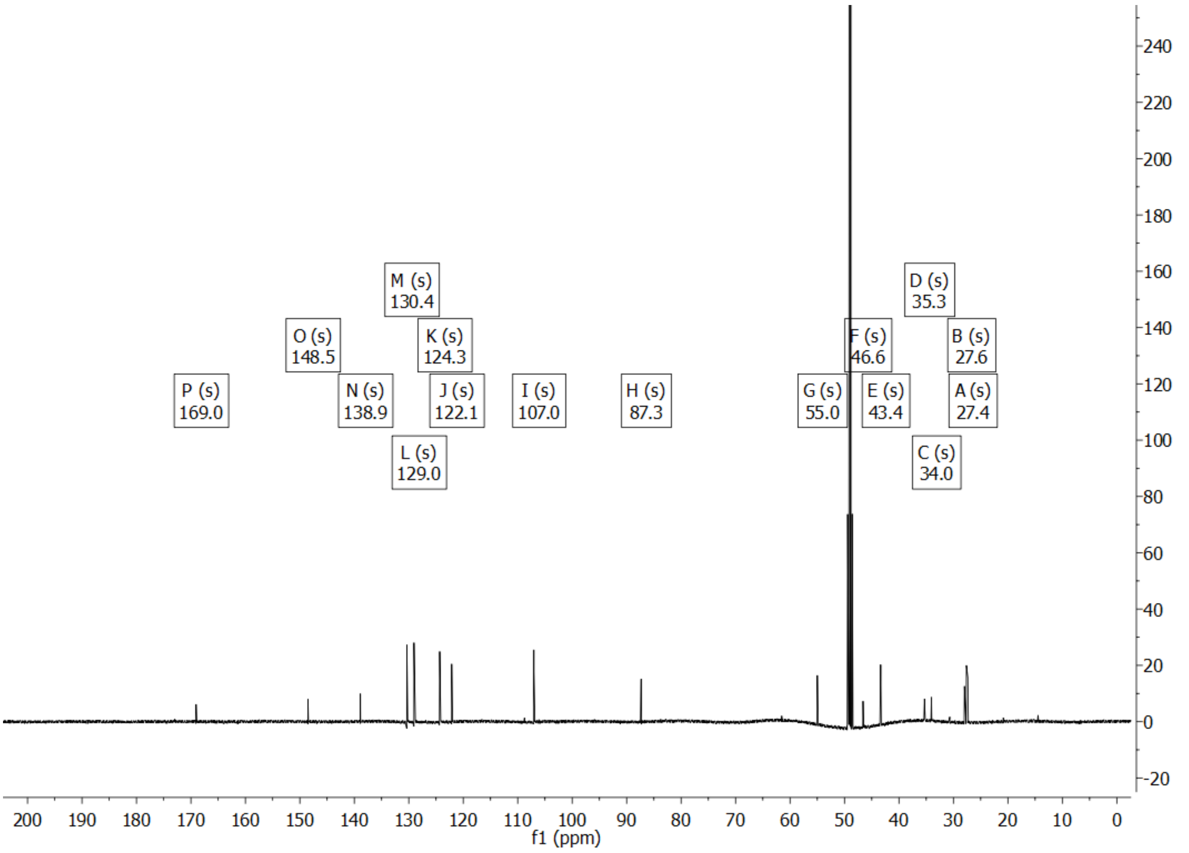

^{1}H- and ^{13}C-NMR-spectrum of *cis*-2-Chloro-N-(3-methoxy-3*H*-spiro[[2]benzopyran-1,1'-cyclohexan]-4'-yl)acetamide (*cis*-**20**) in CD_3OD.

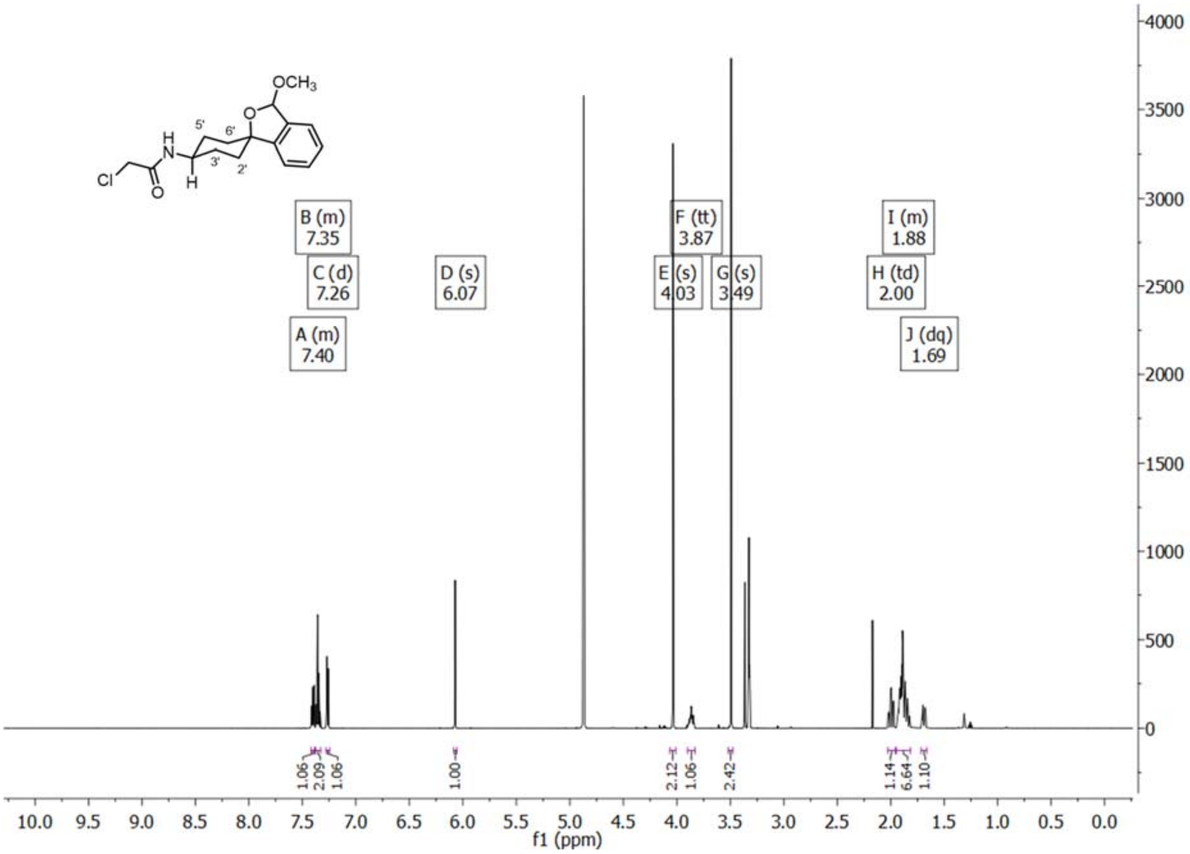

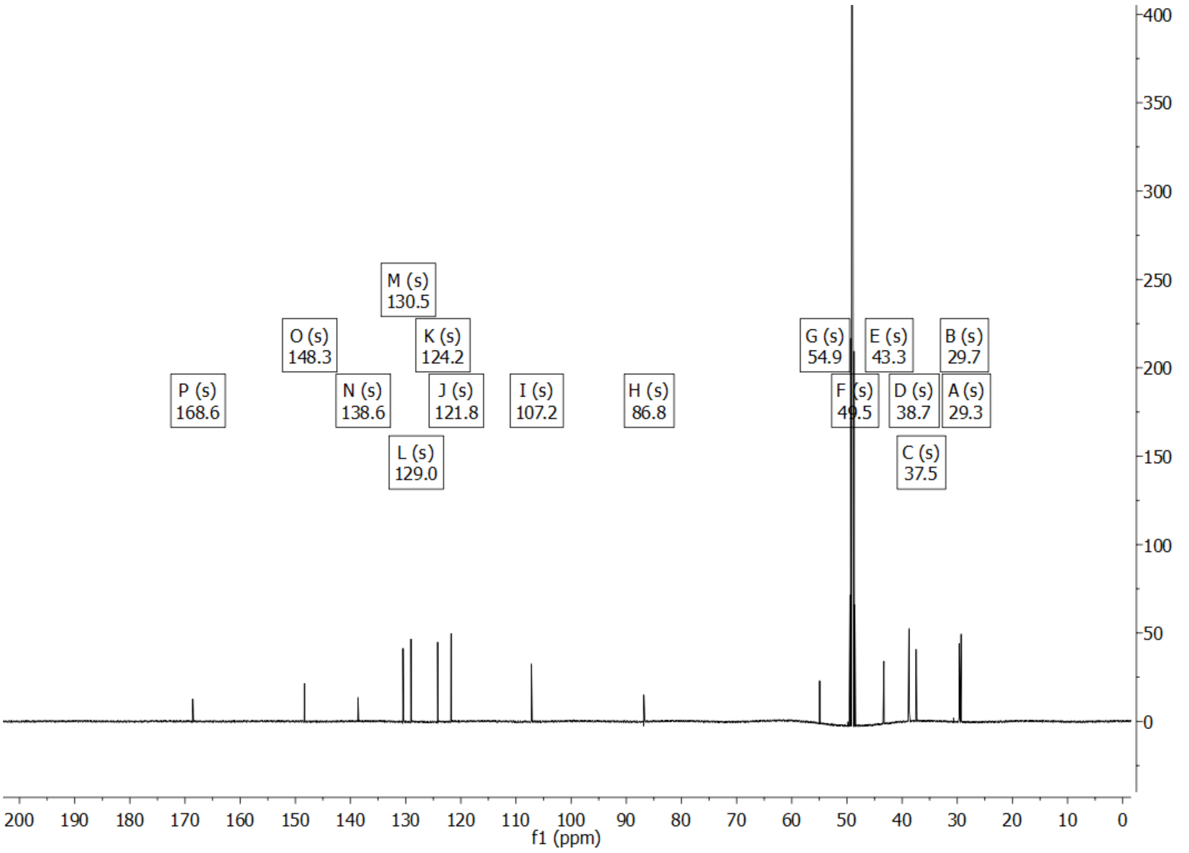

^{1}H- and ^{13}C-NMR-spectrum of 1-(6,7-Dimethoxy-3,4-dihydroisoquinolin-2(1*H*)-yl)-2-{3-methoxy-3,4-dihydrospiro[[2]benzopyran-1,4'-piperidin]-1'-yl}ethan-1-one (**21**) in CD_3OD.

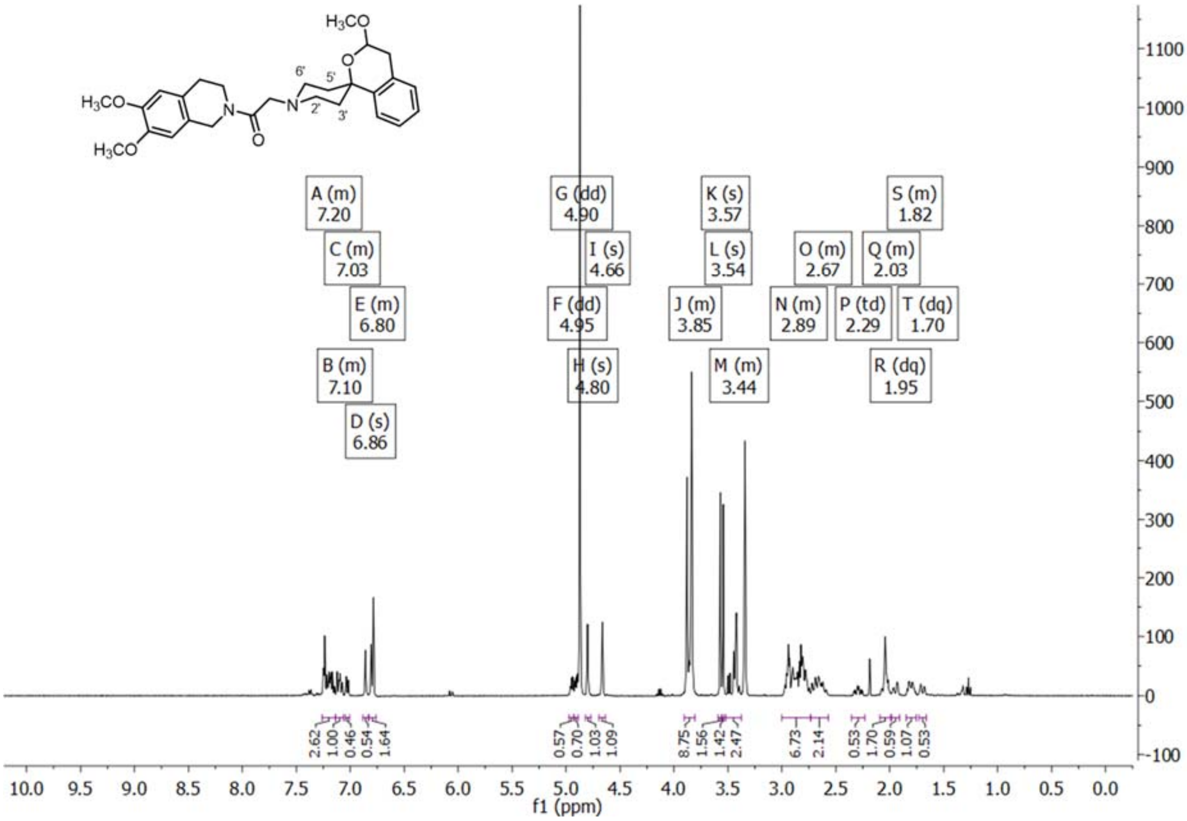

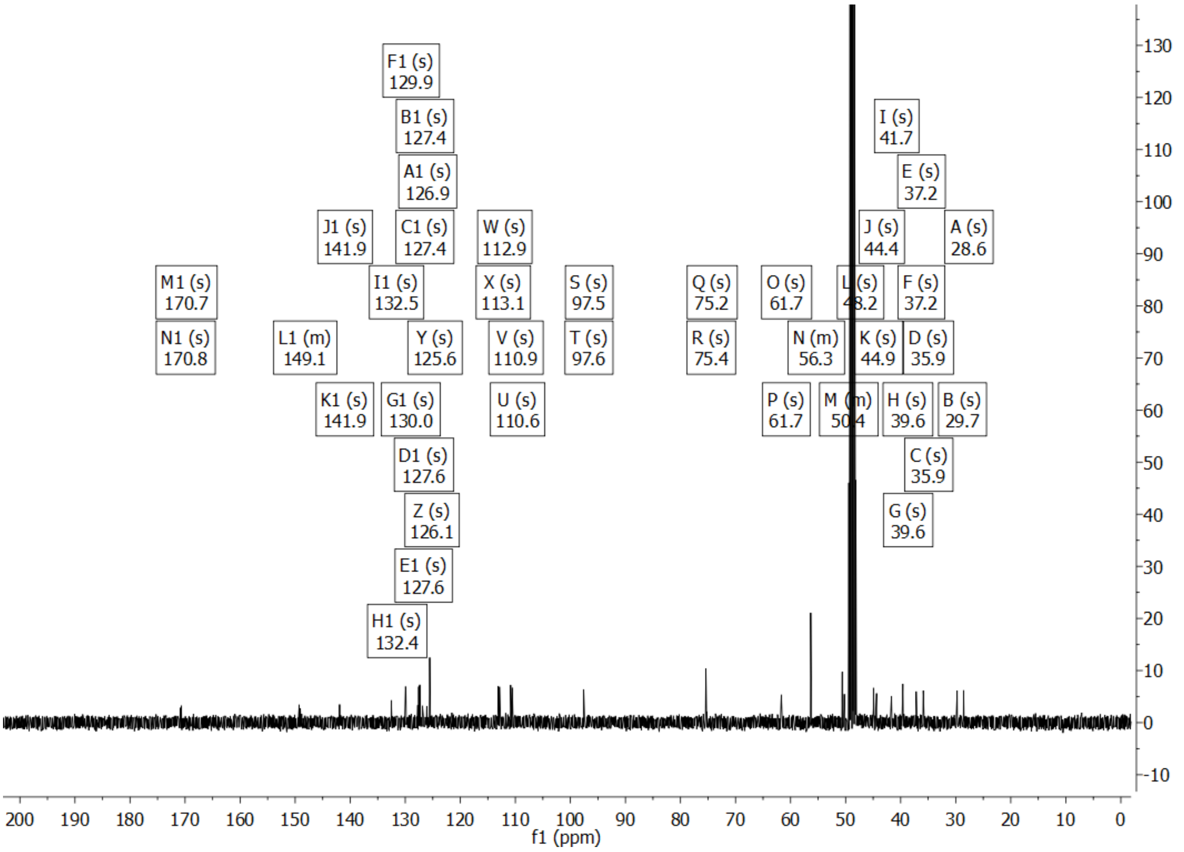

^{1}H- and ^{13}C-NMR-spectrum of *cis*-1-(6,7-Dimethoxy-3,4-dihydroisoquinolin-2(1*H*)-yl)-2-[N-(3-methoxy-3,4-dihydrospiro[[2]benzopyran-1,1'-cyclohexan]-4'-yl)amino]ethan-1-one (*cis*-**22**) in CD3OD.

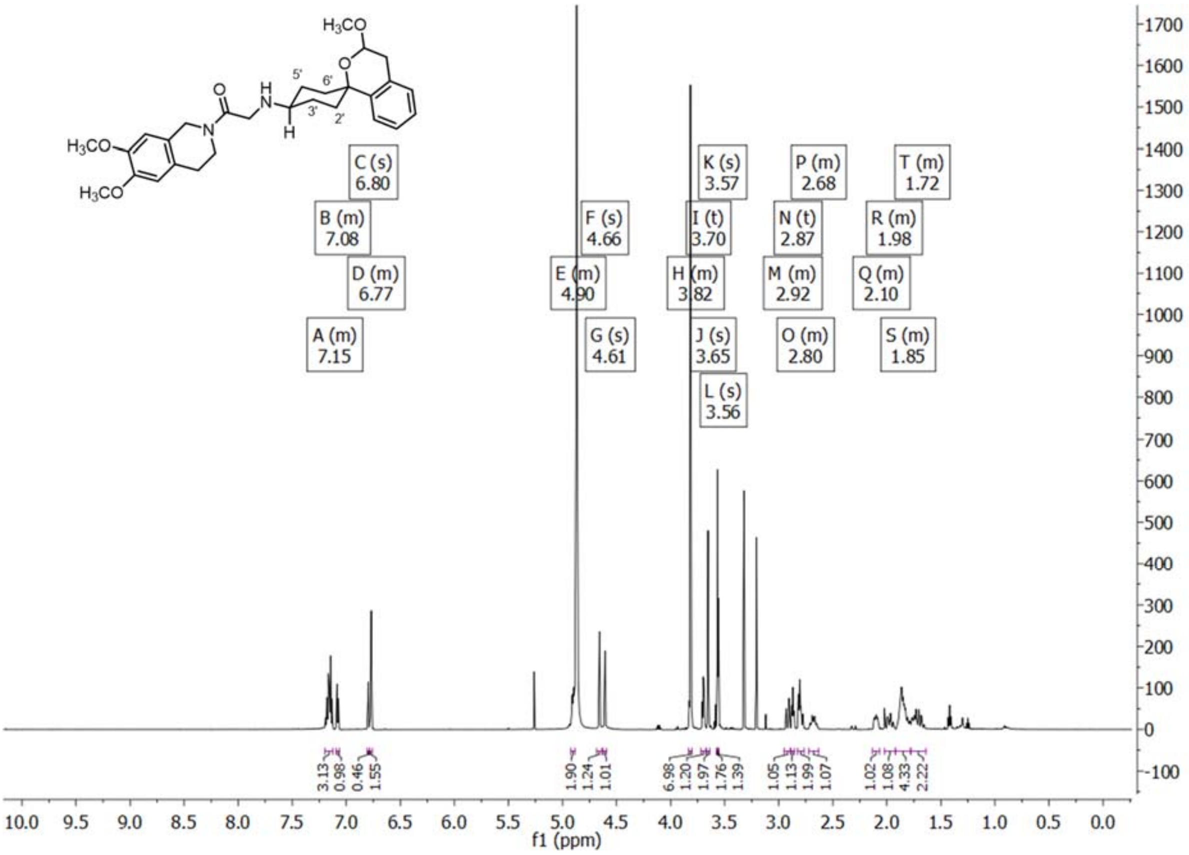

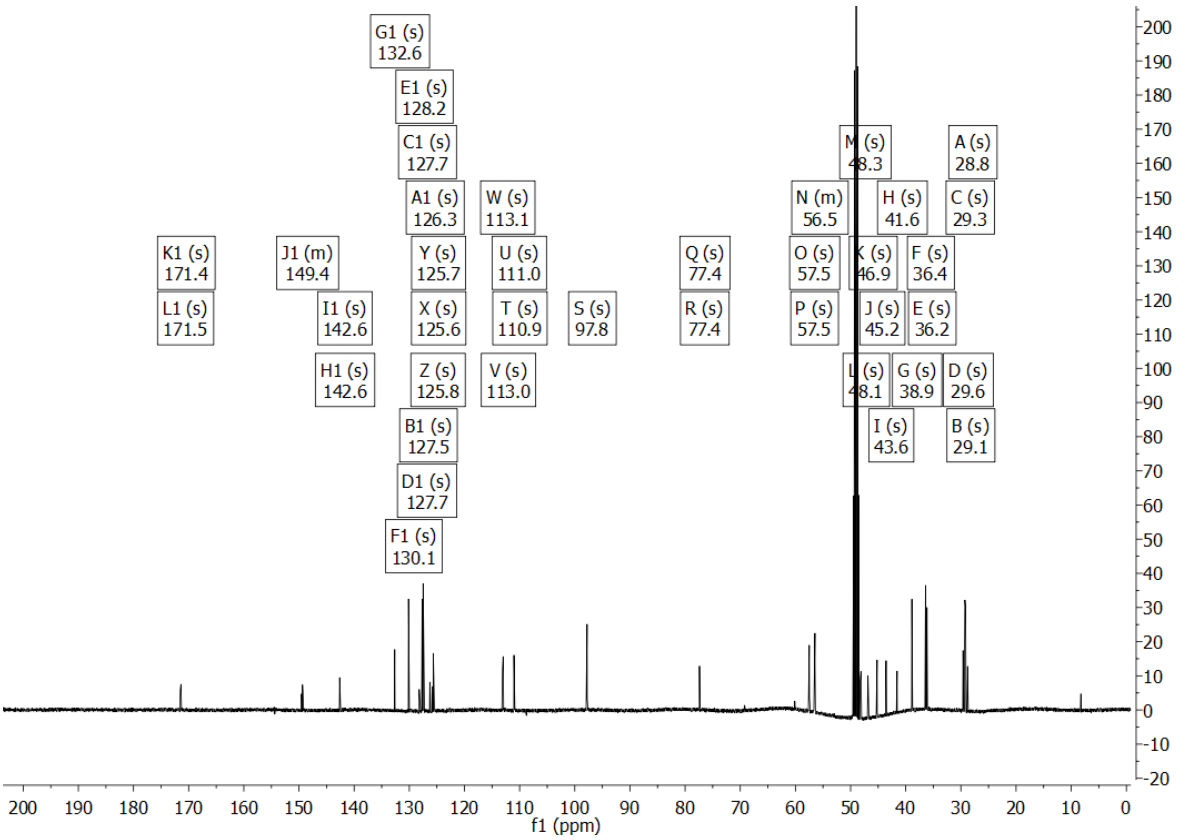

^{1}H- and ^{13}C-NMR-spectrum of 1-(6,7-Dimethoxy-3,4-dihydroisoquinolin-2(1*H*)-yl)-2-{3-methoxy-3*H*-spiro[[2]benzofuran-1,4'-piperidin]-1'-yl}ethan-1-one (**23**) in CD_3OD.

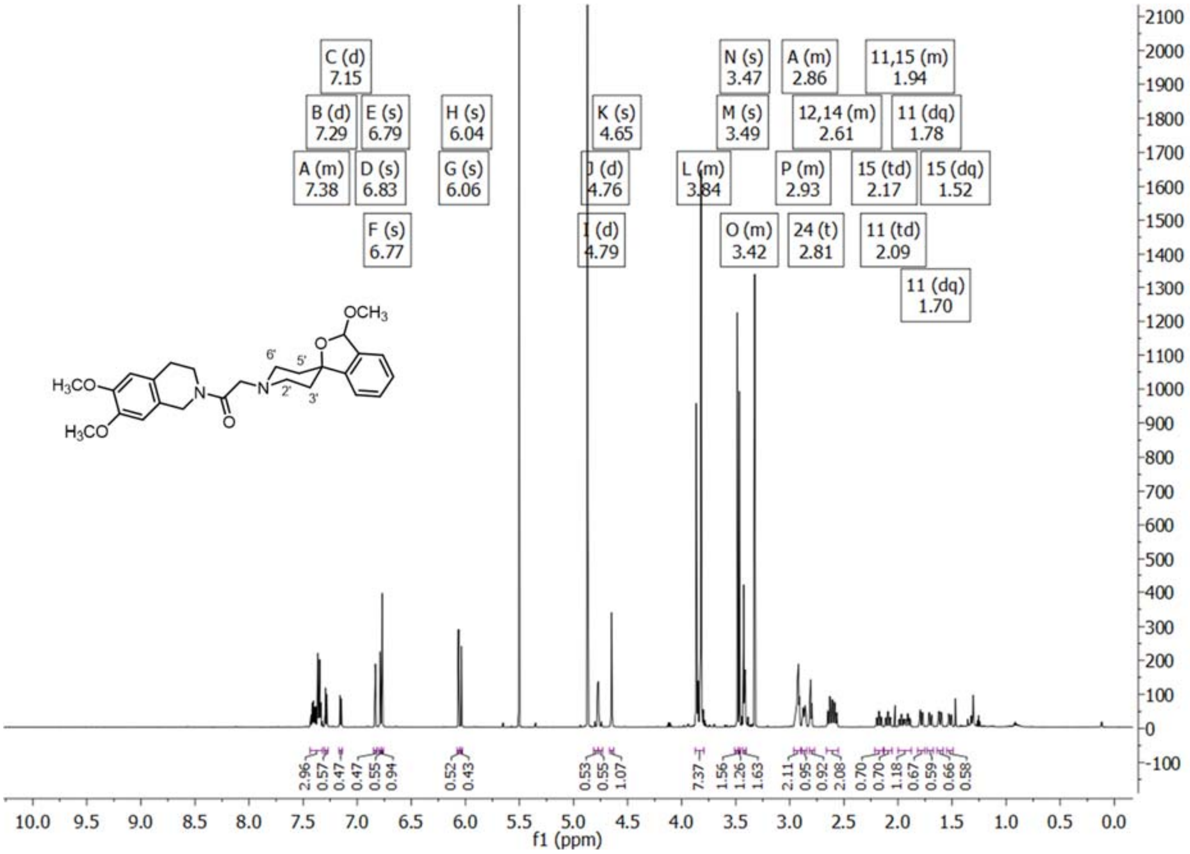

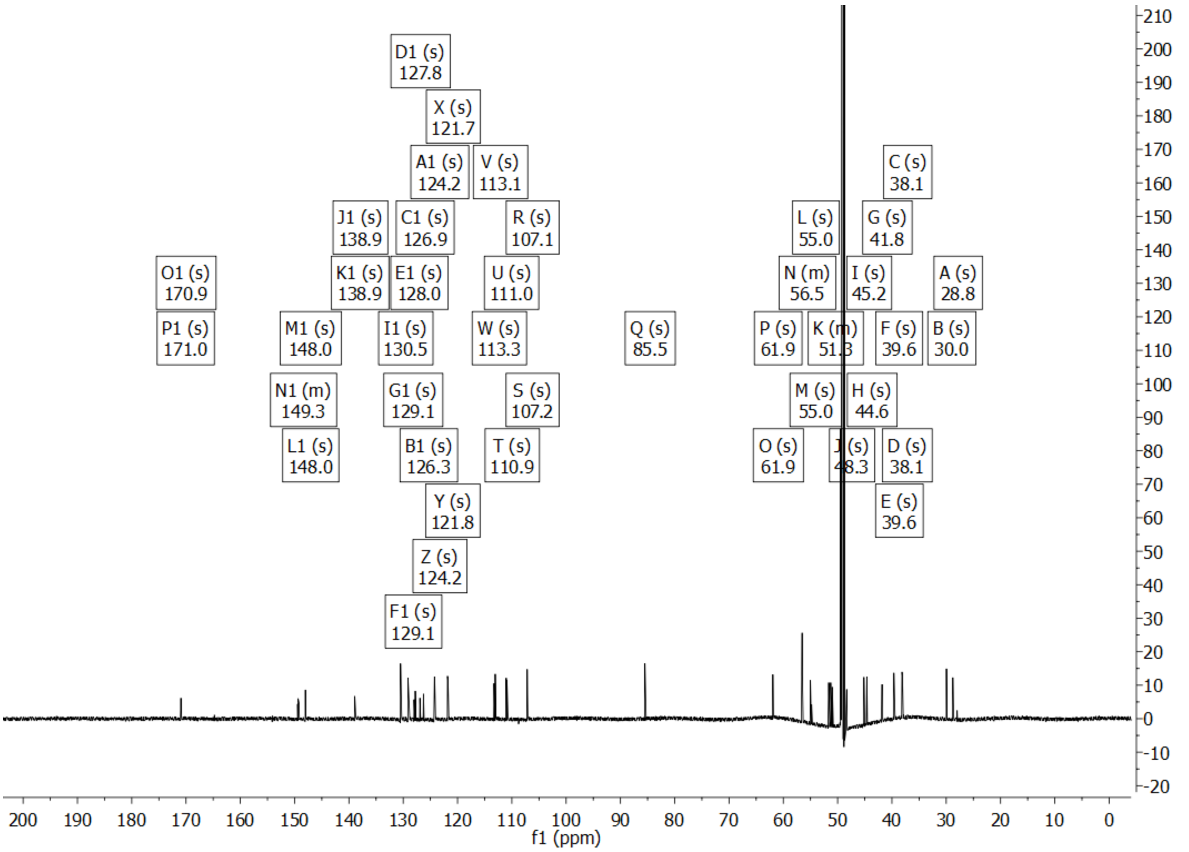

^{1}H- and ^{13}C-NMR-spectrum of *trans*-1-(6,7-Dimethoxy-3,4-dihydroisoquinolin-2(1*H*)-yl)-2-[N-(3-methoxy-3*H*-spiro[[2]benzofuran-1,1’-cyclohexan]-4’-yl)amino]ethan-1-one (*trans*-**24**) in CD_3OD.

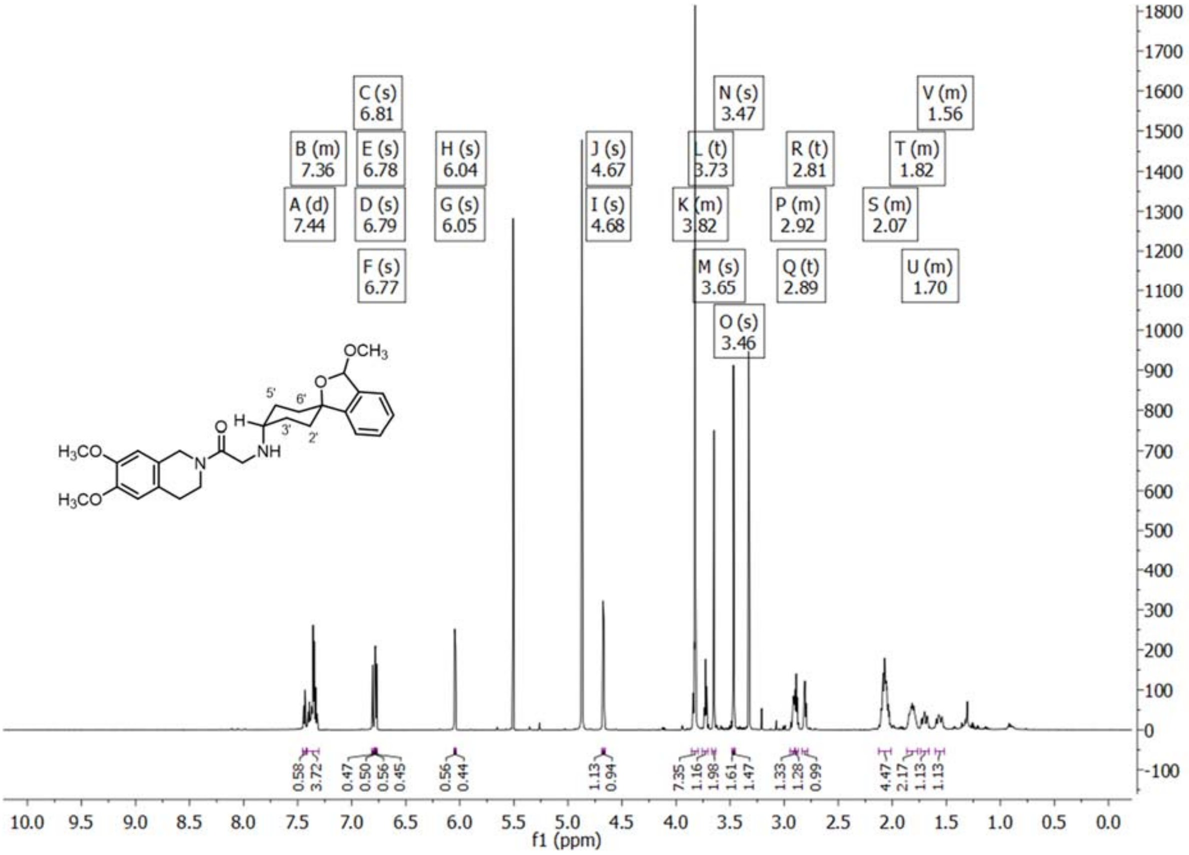

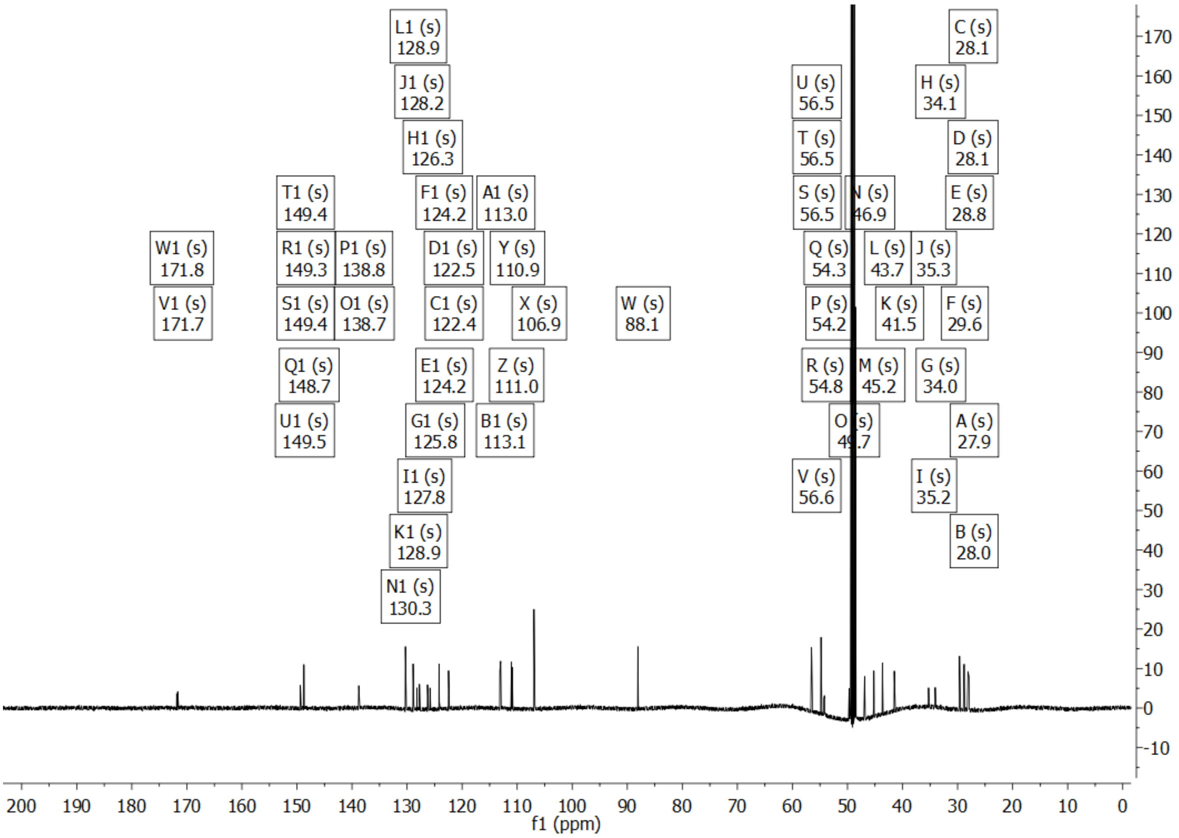

^{1}H- and ^{13}C-NMR-spectrum of *cis*-1-(6,7-Dimethoxy-3,4-dihydroisoquinolin-2(1*H*)-yl)-2-[N-(3-methoxy-3*H*-spiro[[2]benzofuran-1,1'-cyclohexan]-4'-yl)amino]ethan-1-one (*cis*-**24**) in CD_3OD.

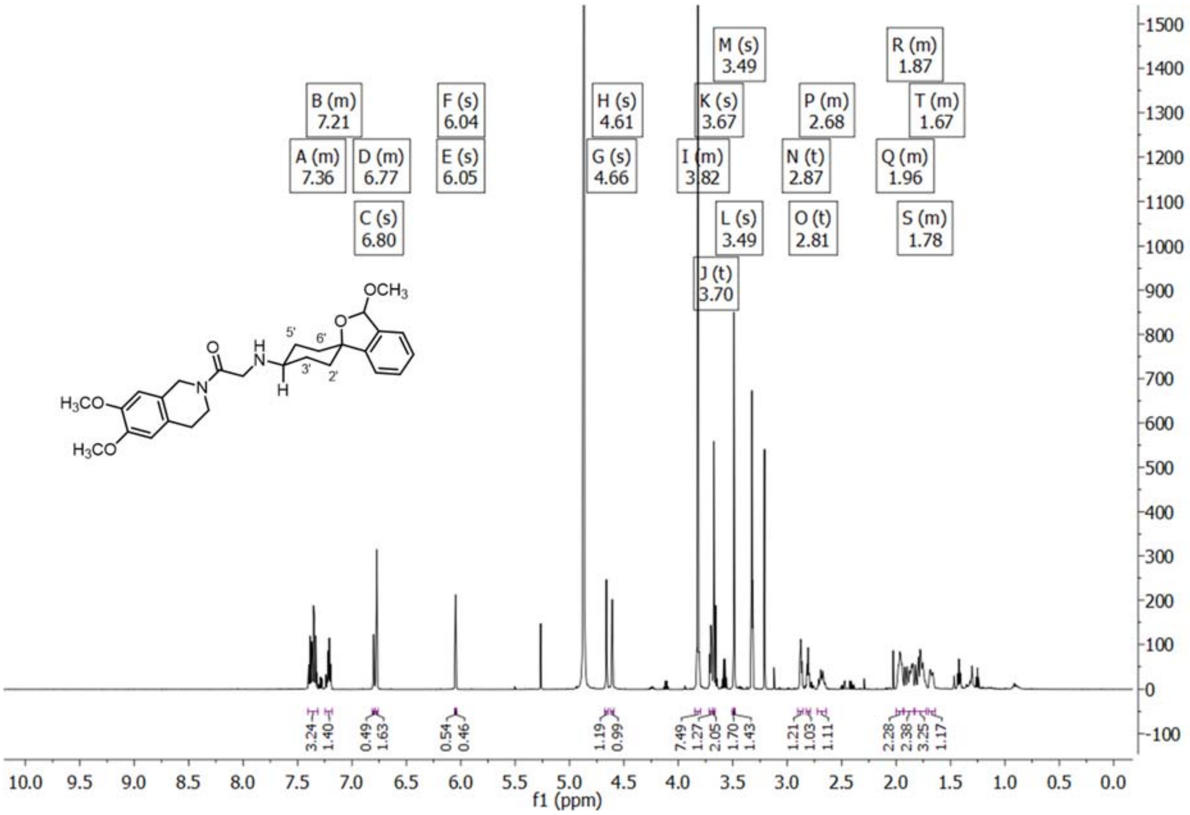

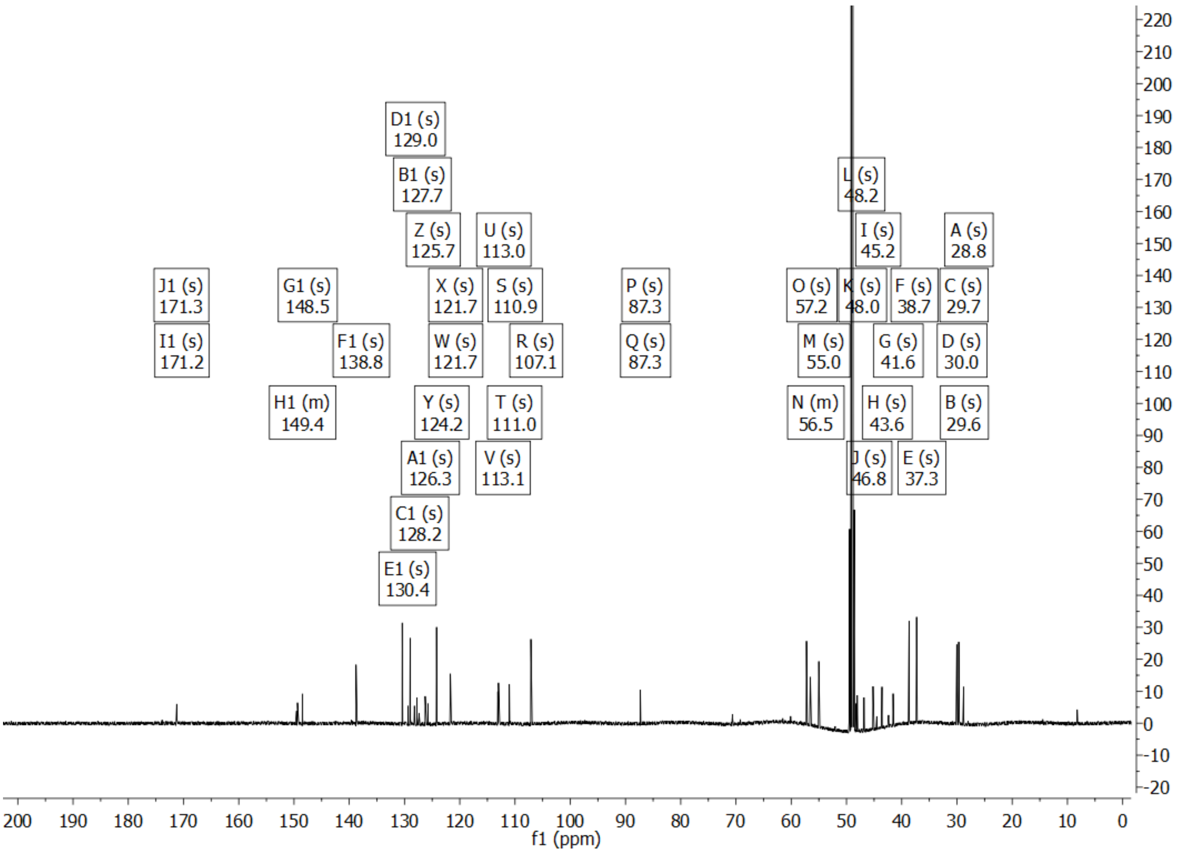

^{1}H- and ^{13}C-NMR-spectrum of 2-(6,7-Dimethoxy-3,4-dihydroisoquinolin-2(1*H*)-yl)-1-(3-methoxy-3,4-dihydrospiro[[2]benzopyran-1,4'-piperidin]-1'-yl)ethan-1-one (**25**) in CD_3OD.

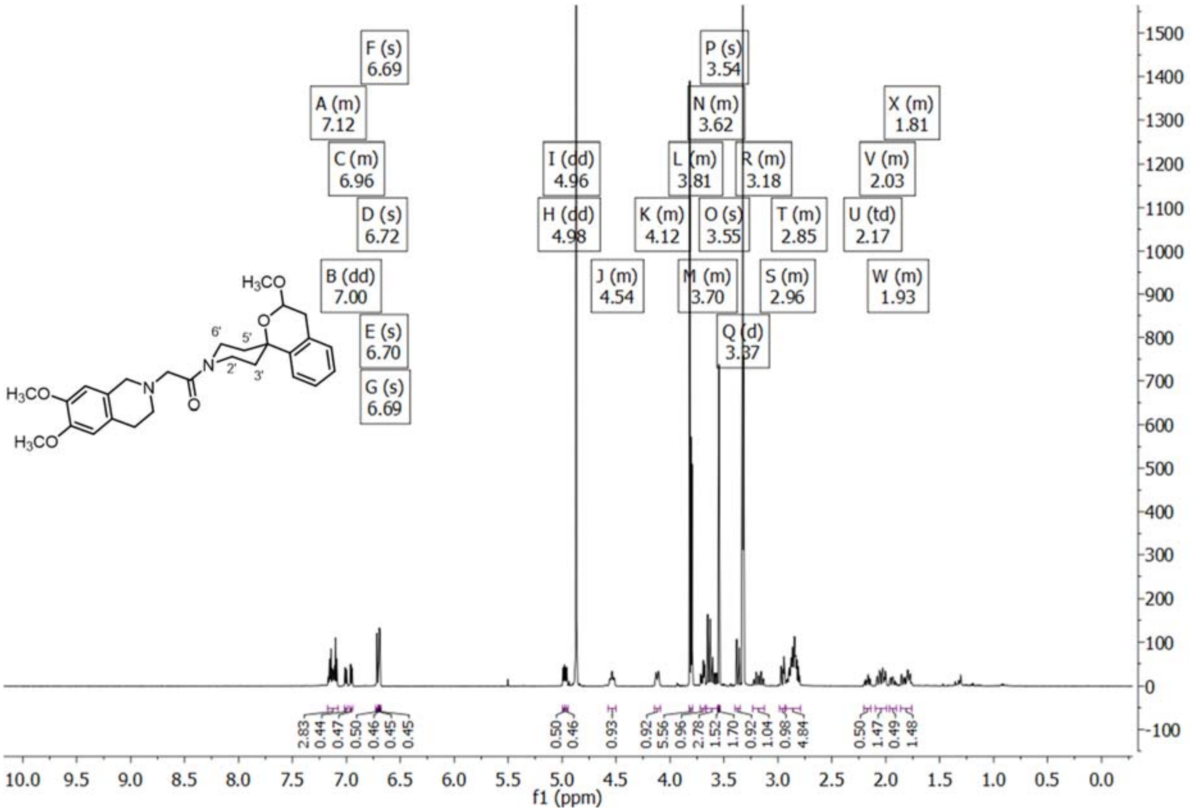

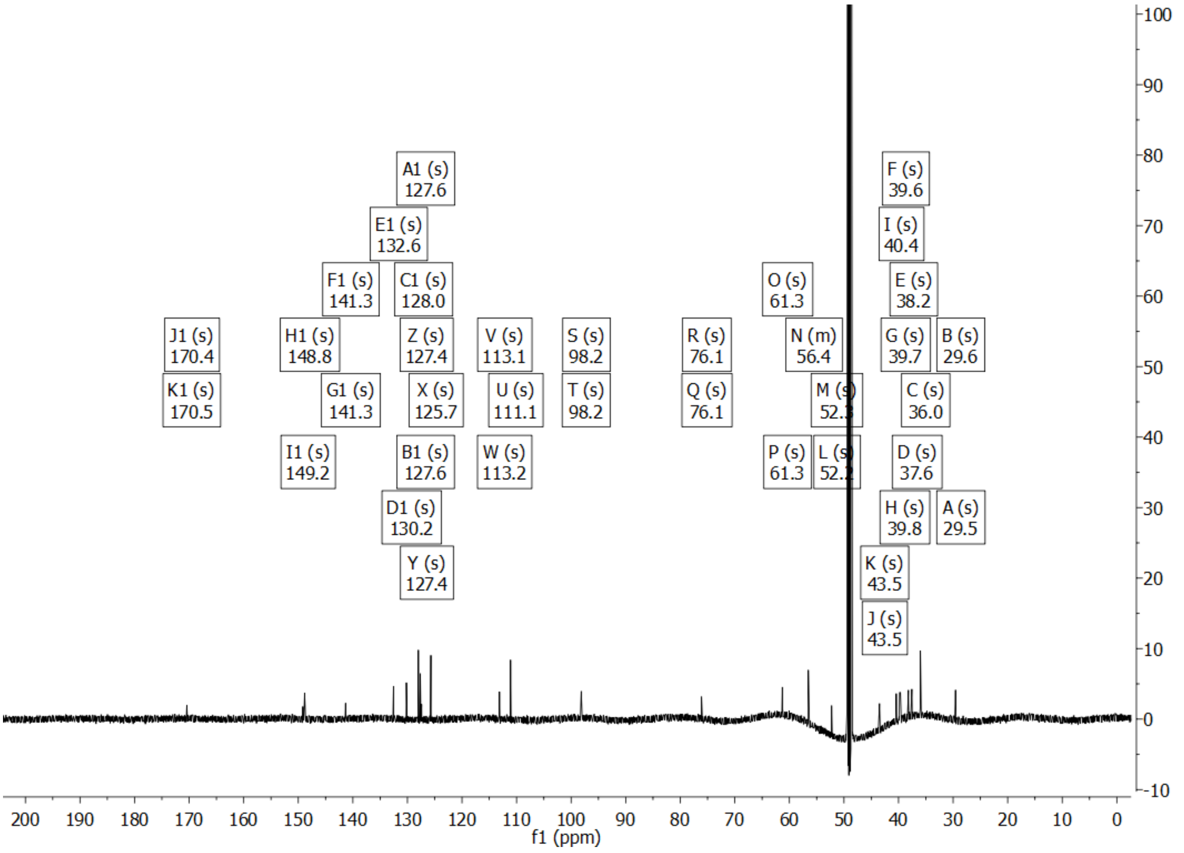

^{1}H- and ^{13}C-NMR-spectrum of *trans*-2-(6,7-Dimethoxy-3,4-dihydroisoquinolin-2(1*H*)-yl)-N-(3-methoxy-3,4-dihydrospiro[[2]benzopyran-1,1'-cyclohexan]-4'-yl)acetamide (*trans*-**26**) in CD_3OD.

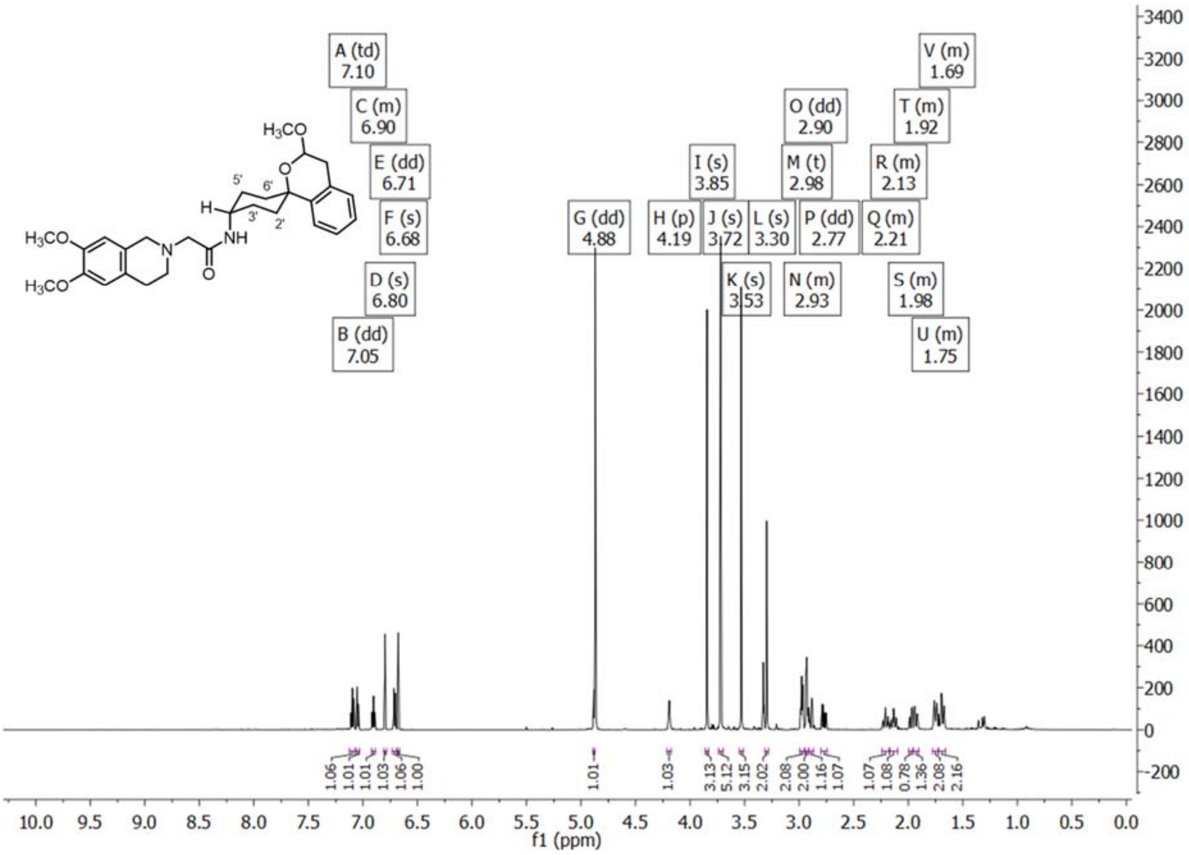

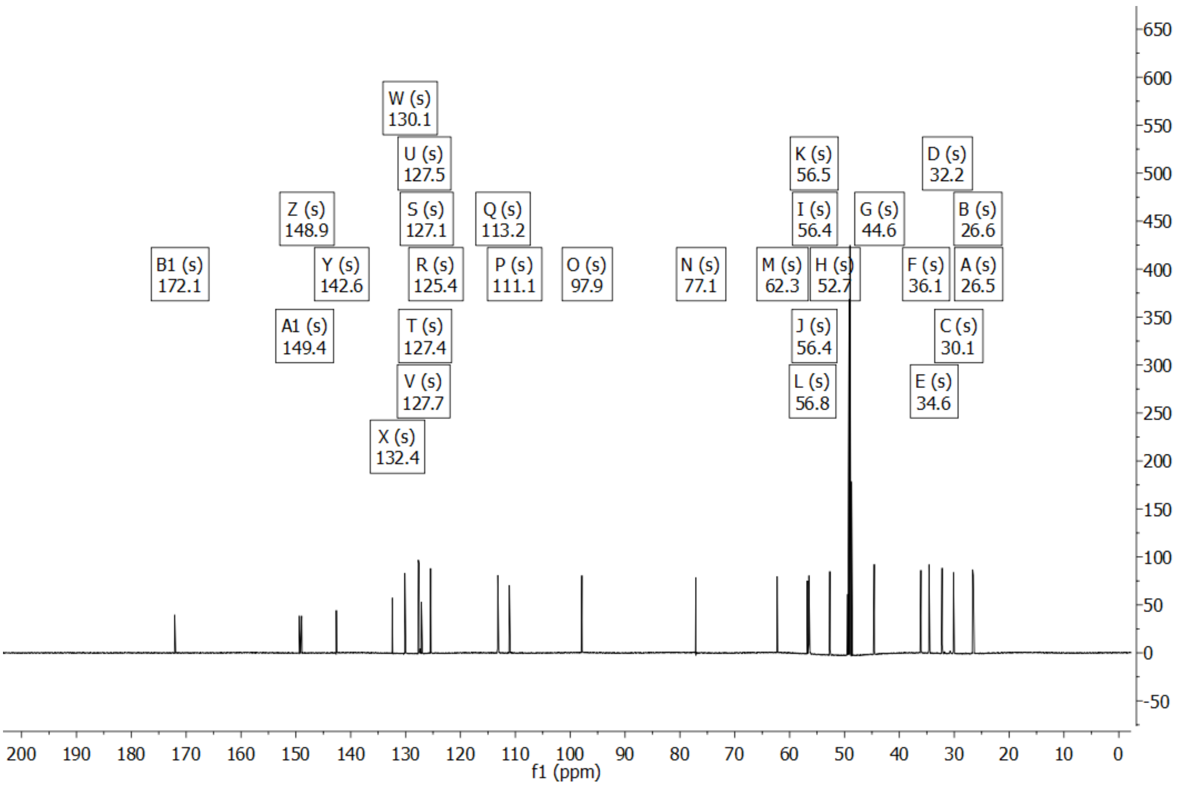

^{1}H- and ^{13}C-NMR-spectrum of *cis*-2-(6,7-Dimethoxy-3,4-dihydroisoquinolin-2(1*H*)-yl)-N-(3-methoxy-3,4-dihydrospiro[[2]benzopyran-1,1'-cyclohexan]-4'-yl)acetamide (*cis*-**26**) in CD3OD.

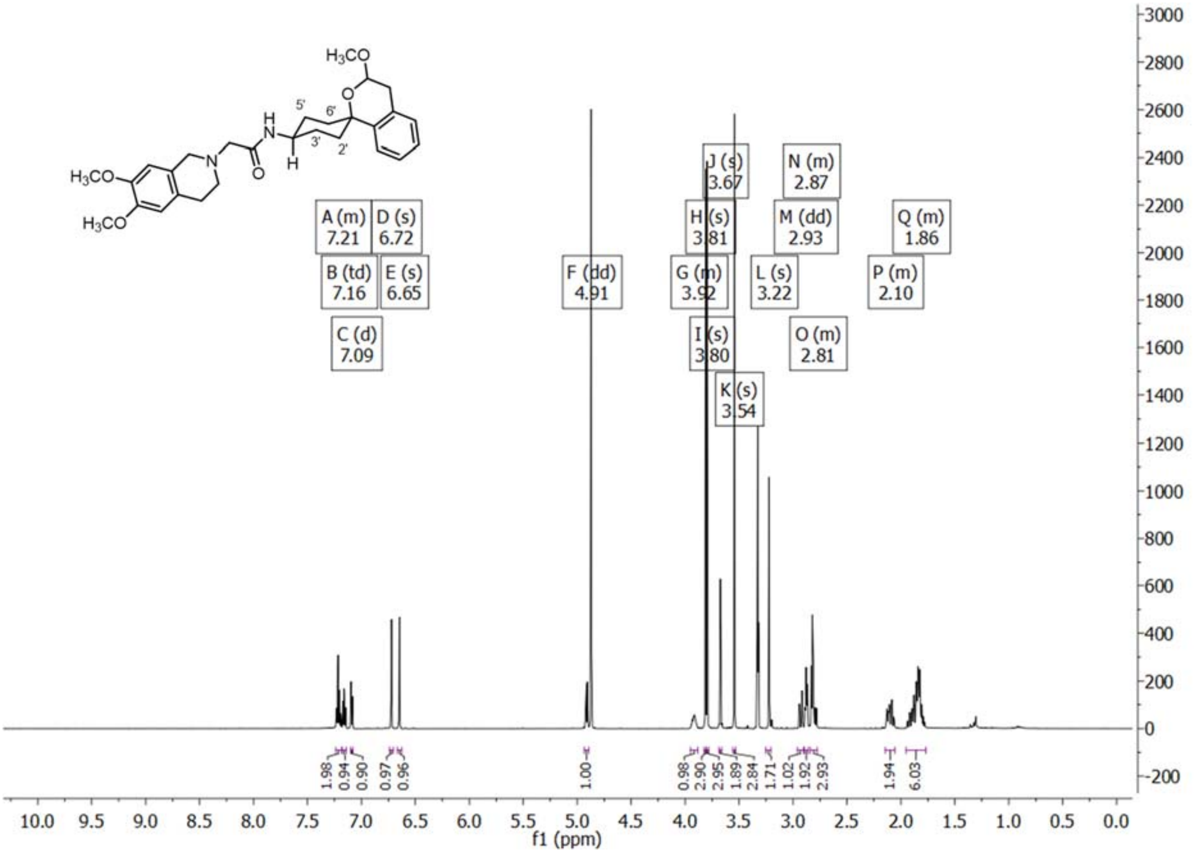

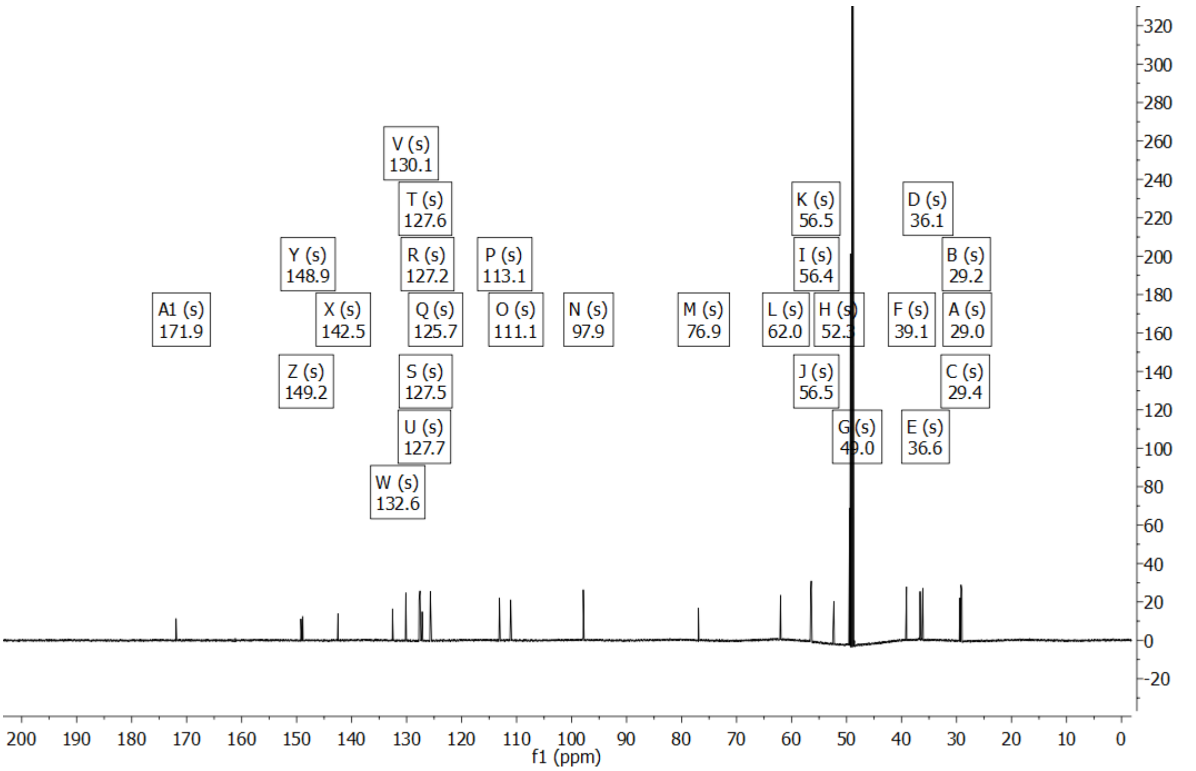

^{1}H- and ^{13}C-NMR-spectrum of *cis*-4-(6,7-Dimethoxy-3,4-dihydroisoquinolin-2(1*H*)-yl)-N-(3-methoxy-3,4-dihydrospiro[[2]benzopyran-1,1'-cyclohexan]-4'-yl)butanamide (*cis*-**27**) in CD_3OD.

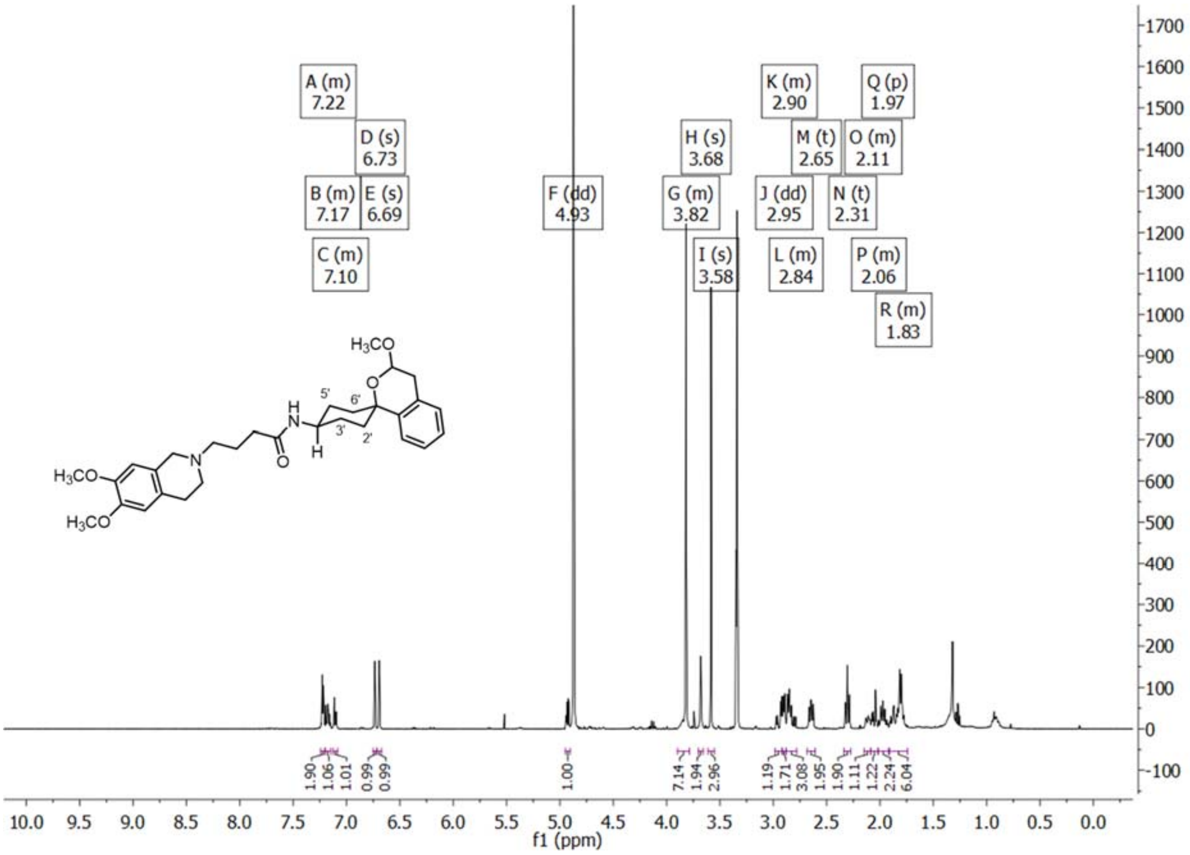

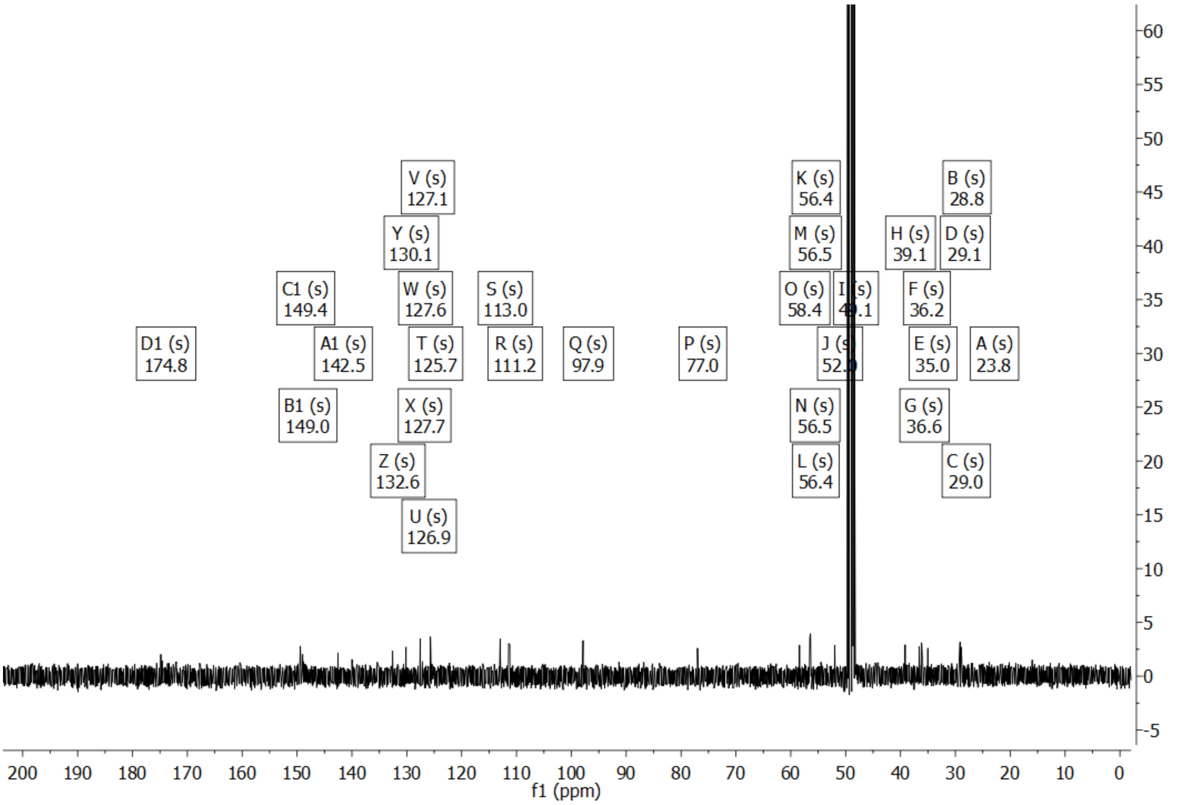

^{1}H- and ^{13}C-NMR-spectrum of 2-(6,7-Dimethoxy-3,4-dihydroisoquinolin-2(1*H*)-yl)-1-{3-methoxy-3*H*-spiro[[2]benzofuran-1,4'-piperidin]-1'-yl}ethan-1-one (**28**) in CD_3OD.

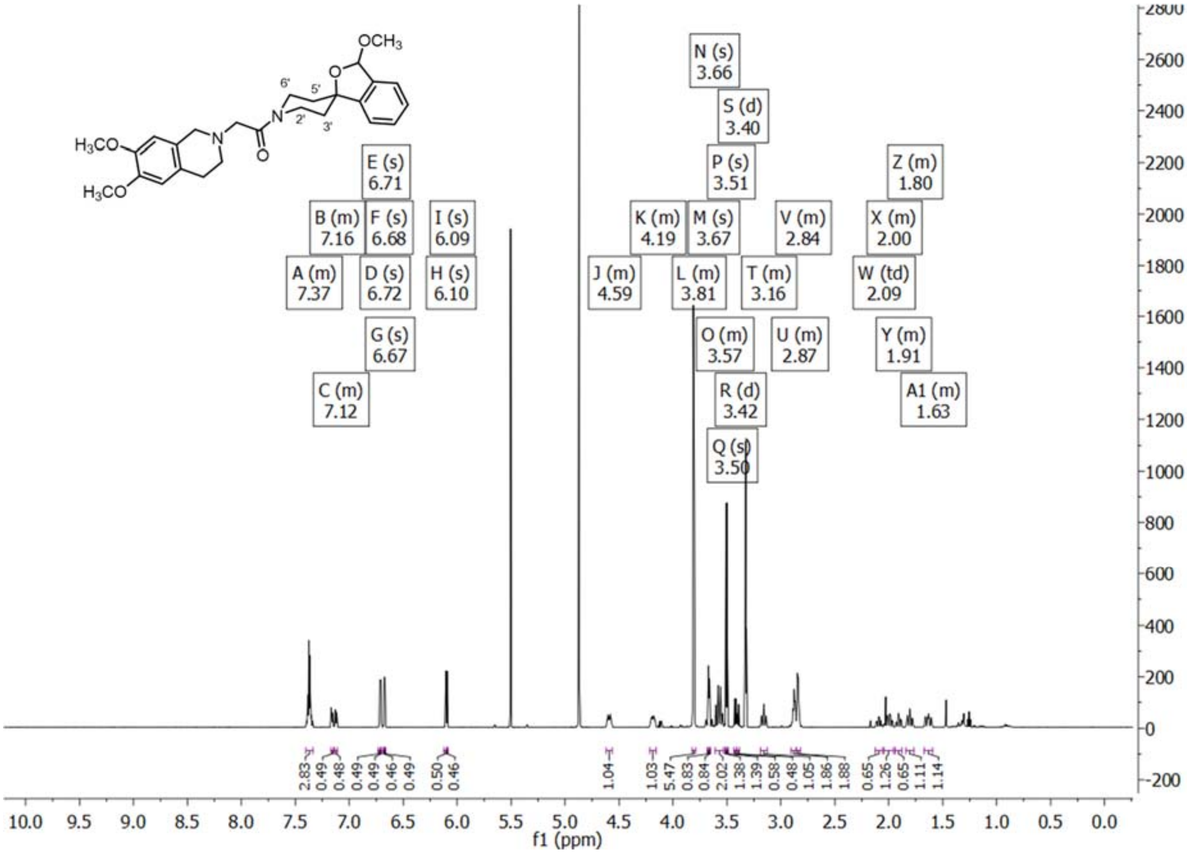

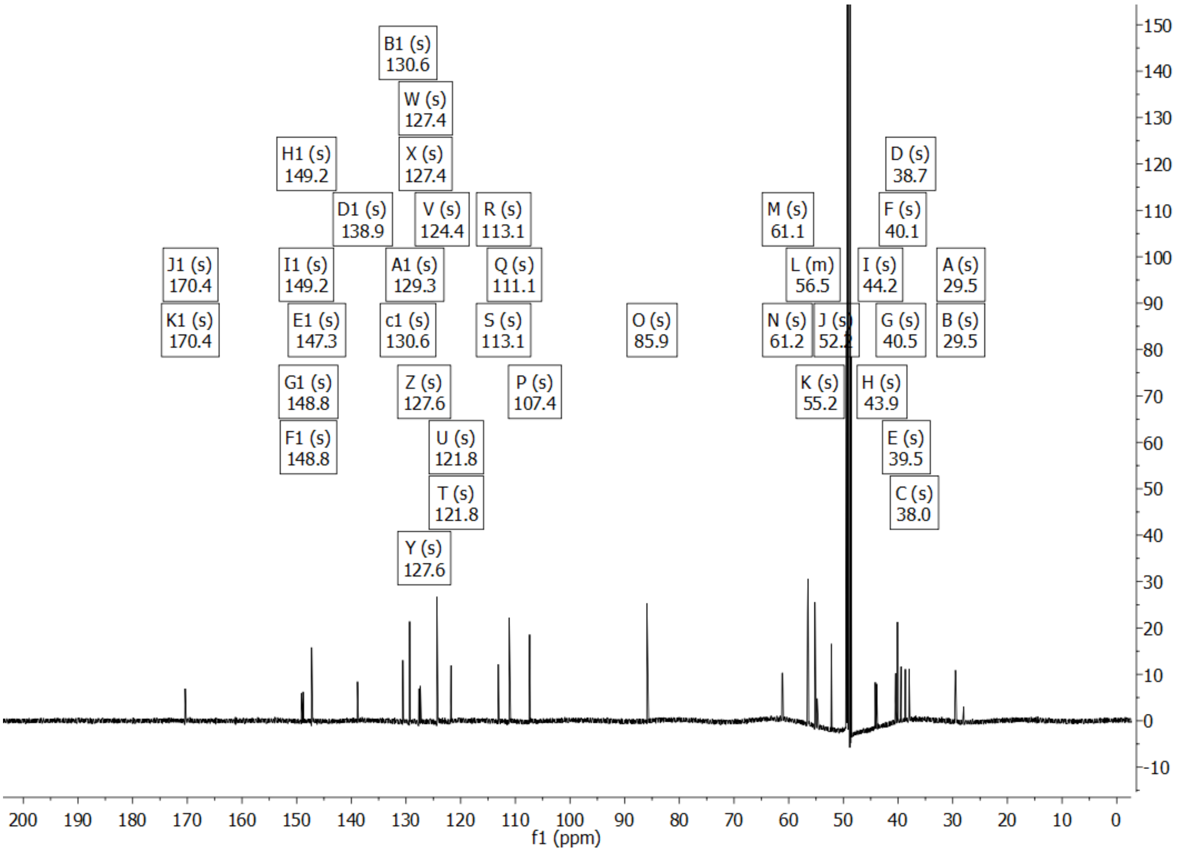

^{1}H- and ^{13}C-NMR-spectrum of *trans*-2-(6,7-Dimethoxy-3,4-dihydroisoquinolin-2(1*H*)-yl)-N-(3-methoxy-3*H*-spiro[[2]benzofuran-1,1'-cyclohexan]-4'-yl)acetamide (*trans*-**29**) in CD_3OD.

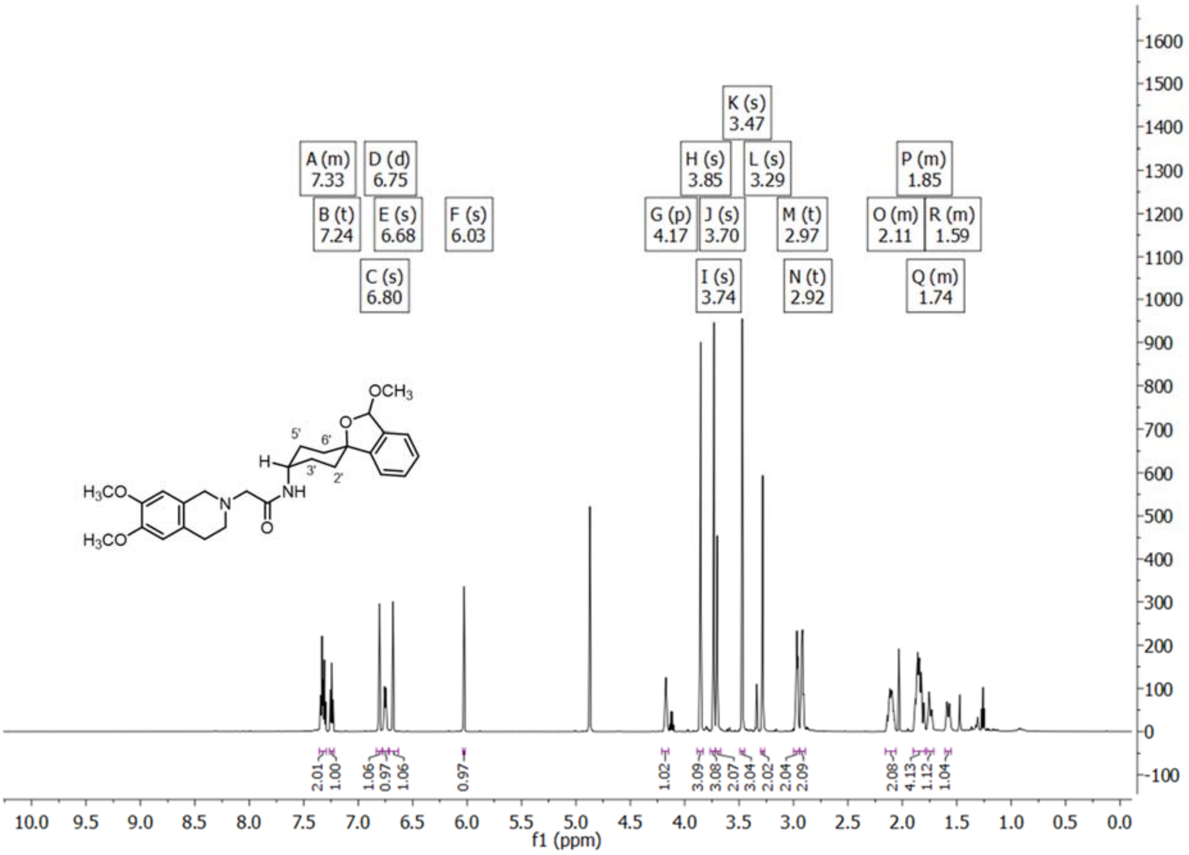

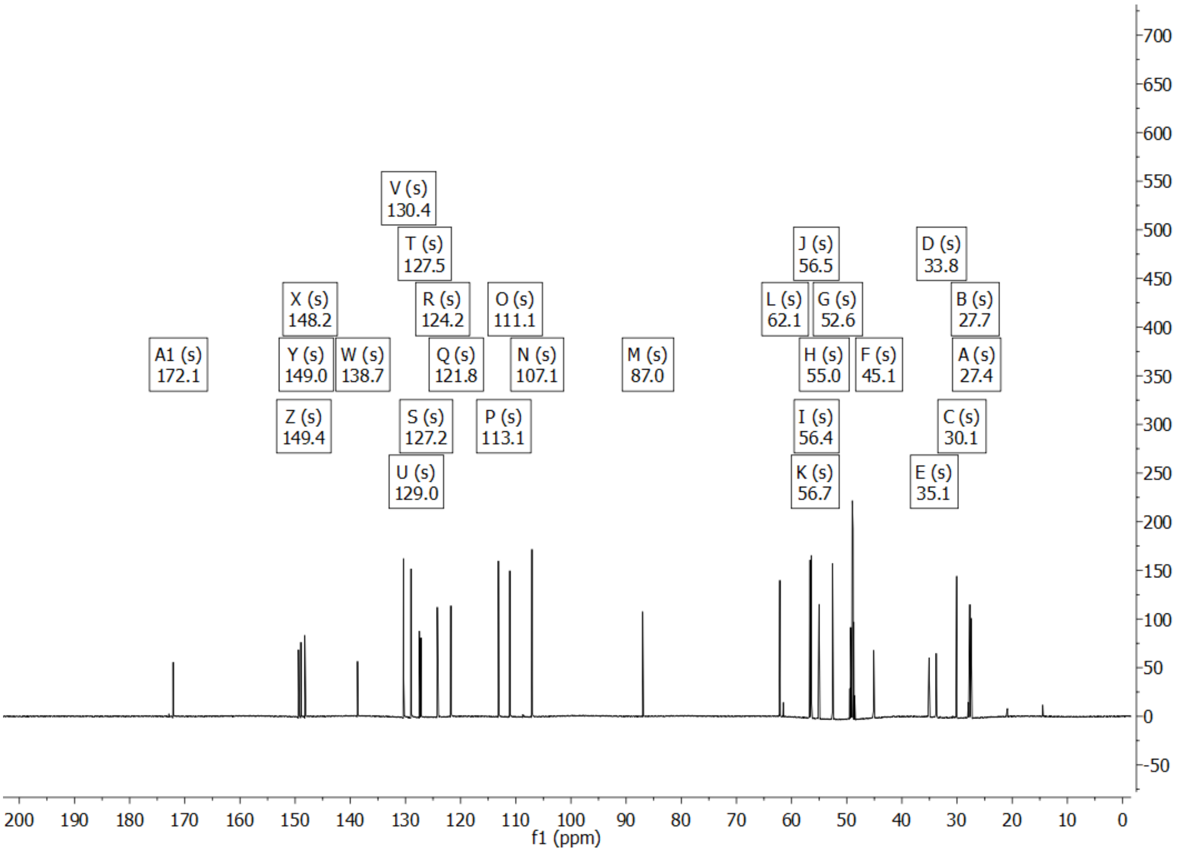

^{1}H- and ^{13}C-NMR-spectrum of *cis*-2-(6,7-Dimethoxy-3,4-dihydroisoquinolin-2(1*H*)-yl)-N-(3-methoxy-3*H*-spiro[[2]benzofuran-1,1'-cyclohexan]-4'-yl)acetamide (*cis*-**29**) in CD_3OD.

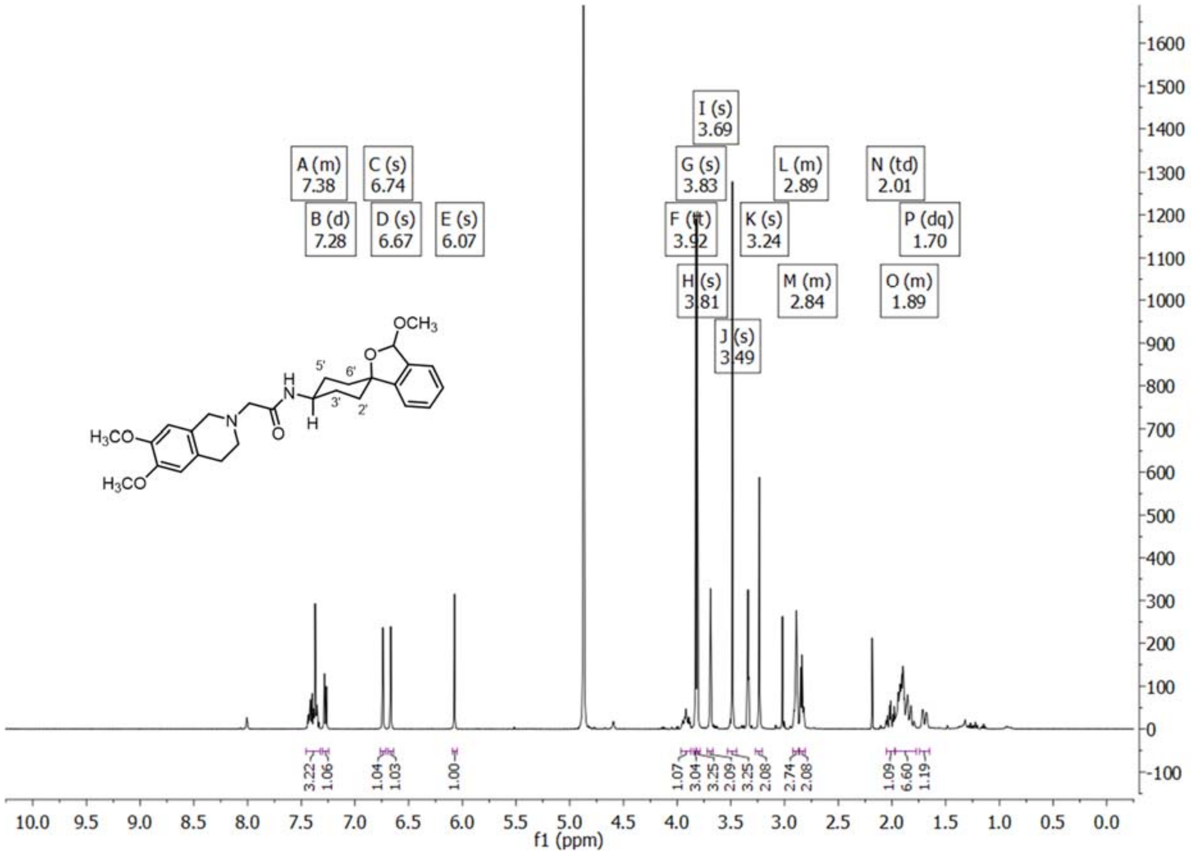

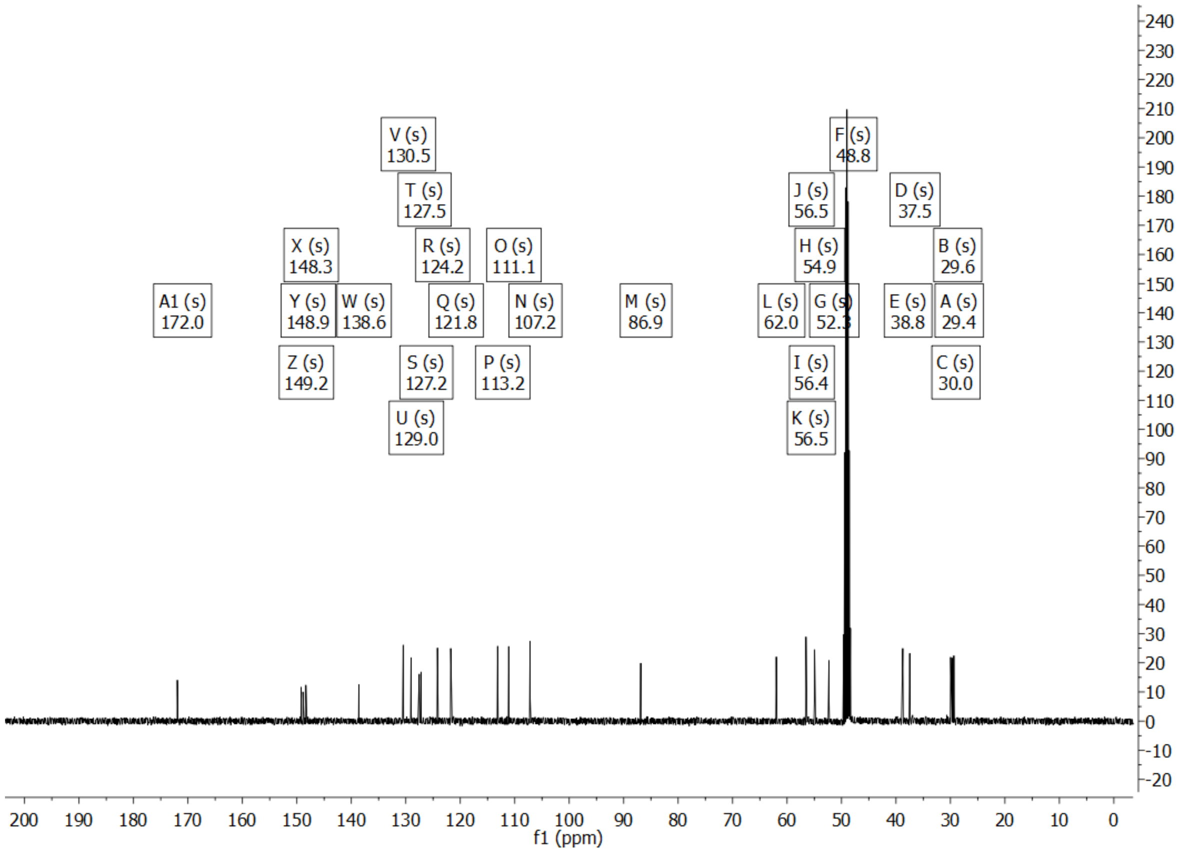

^{1}H- and ^{13}C-NMR-spectrum of 1’-[2-(6,7-Dimethoxy-3,4-dihydroisoquinolin-2(1*H*)-yl)ethyl]-3-methoxy-3,4-dihydrospiro[[2]benzopyran-1,4’-piperidine] (**30**) in CD_3OD.

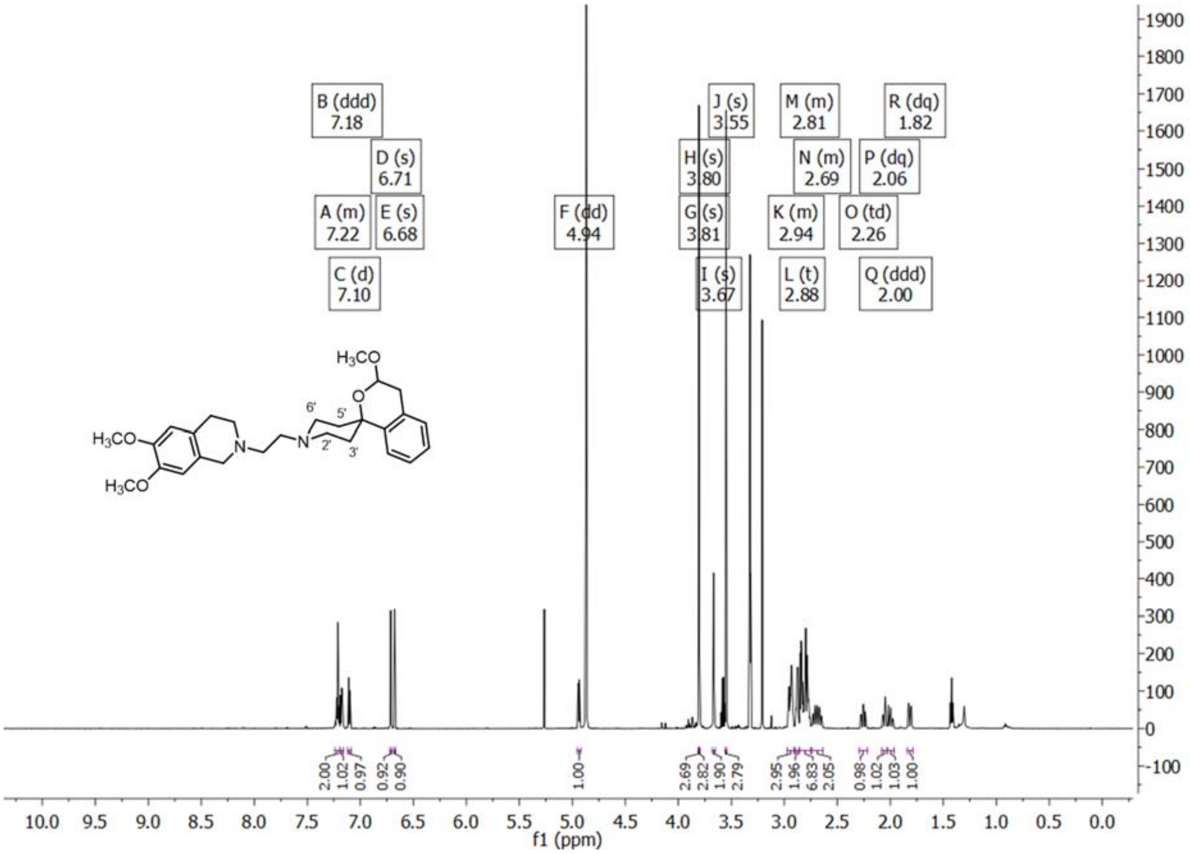

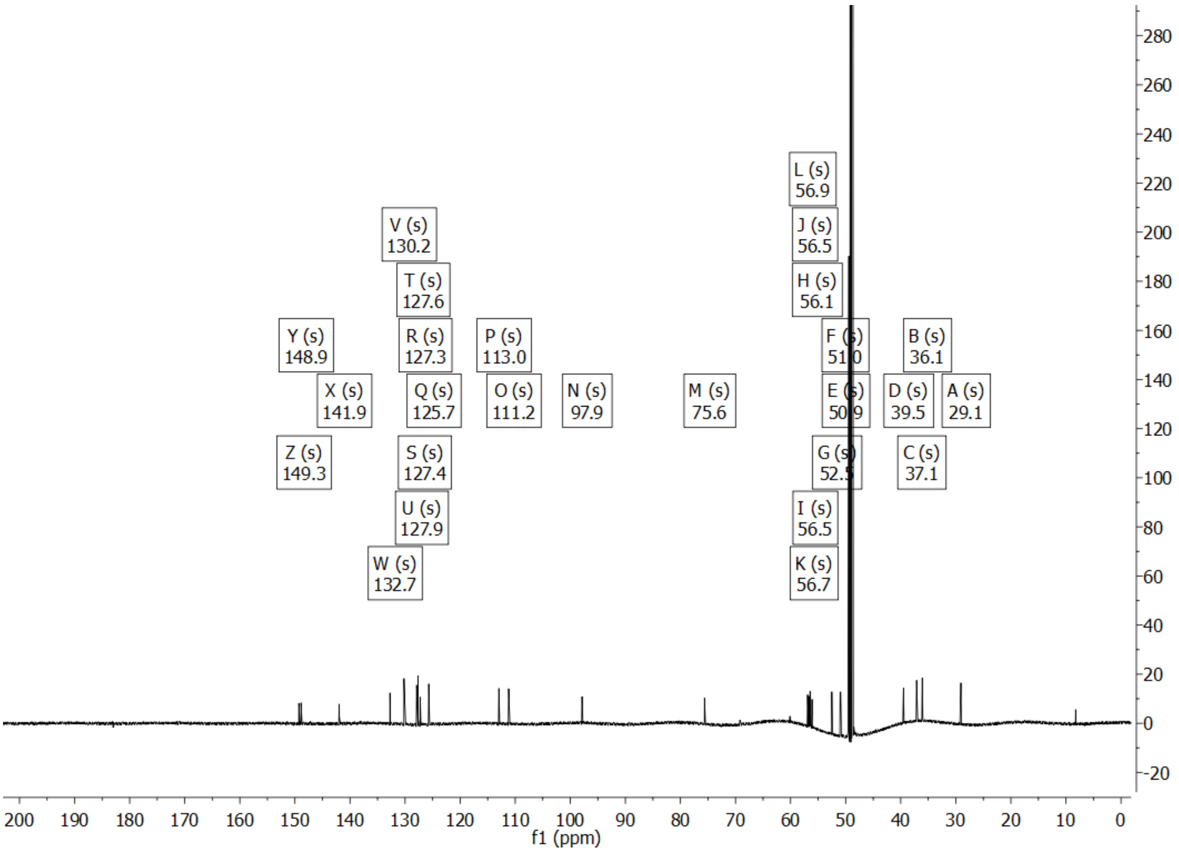

^{1}H- and ^{13}C-NMR-spectrum of *trans*-N-[2-(6,7-Dimethoxy-3,4-dihydroisoquinolin-2(1*H*)-yl)ethyl]-3-methoxy-3,4-dihydrospiro[[2]benzopyran-1,1'-cyclohexan]-4'-amine (*trans*-**31**) in CD_3OD.

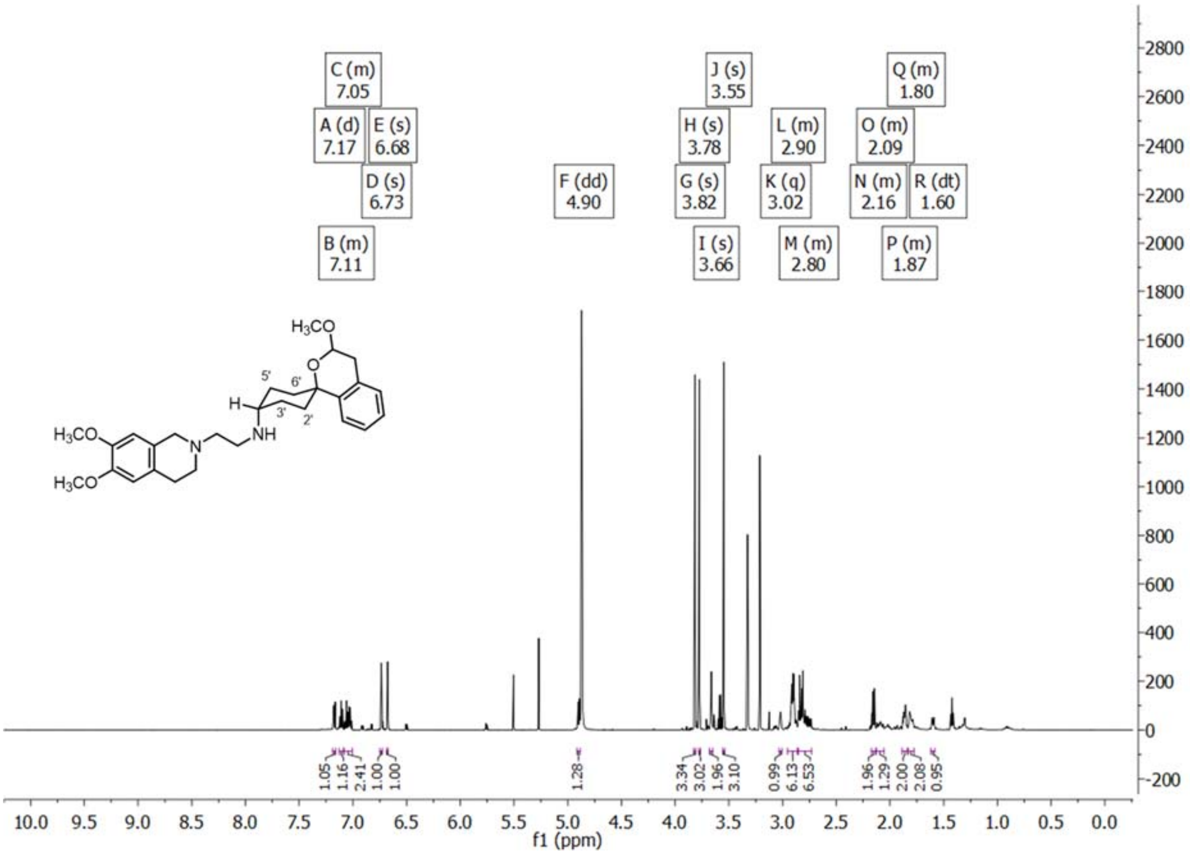

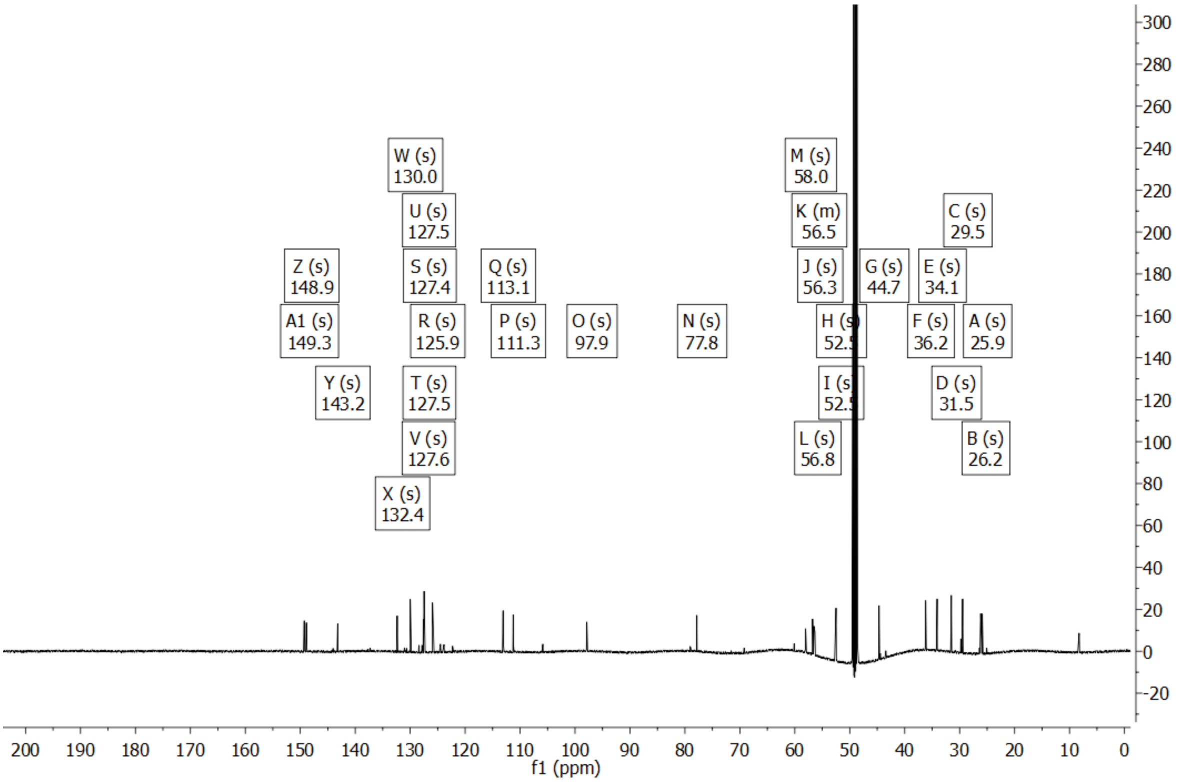

^{1}H- and ^{13}C-NMR-spectrum of *cis*-N-[2-(6,7-Dimethoxy-3,4-dihydroisoquinolin-2(1*H*)-yl)ethyl]-3-methoxy-3,4-dihydrospiro[[2]benzopyran-1,1'-cyclohexan]-4'-amine (*cis*-**31**) in CD_3OD.

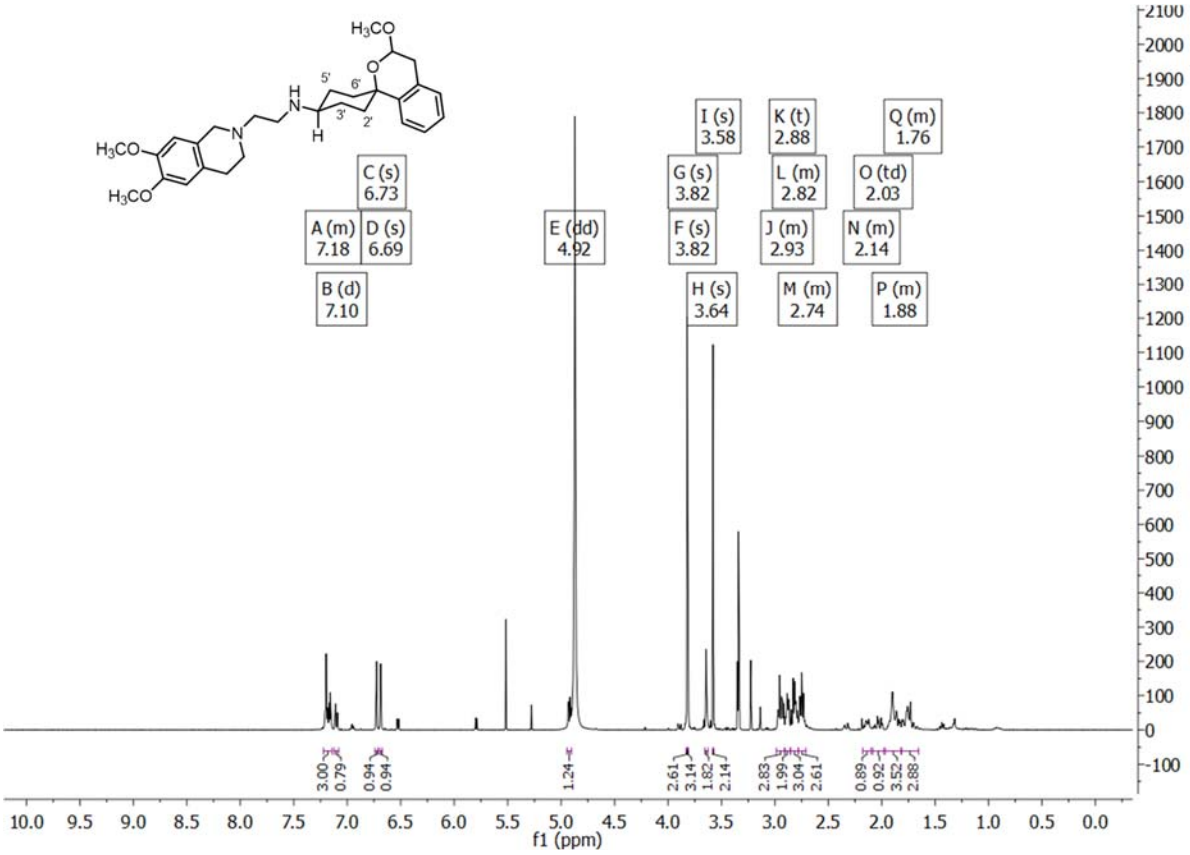

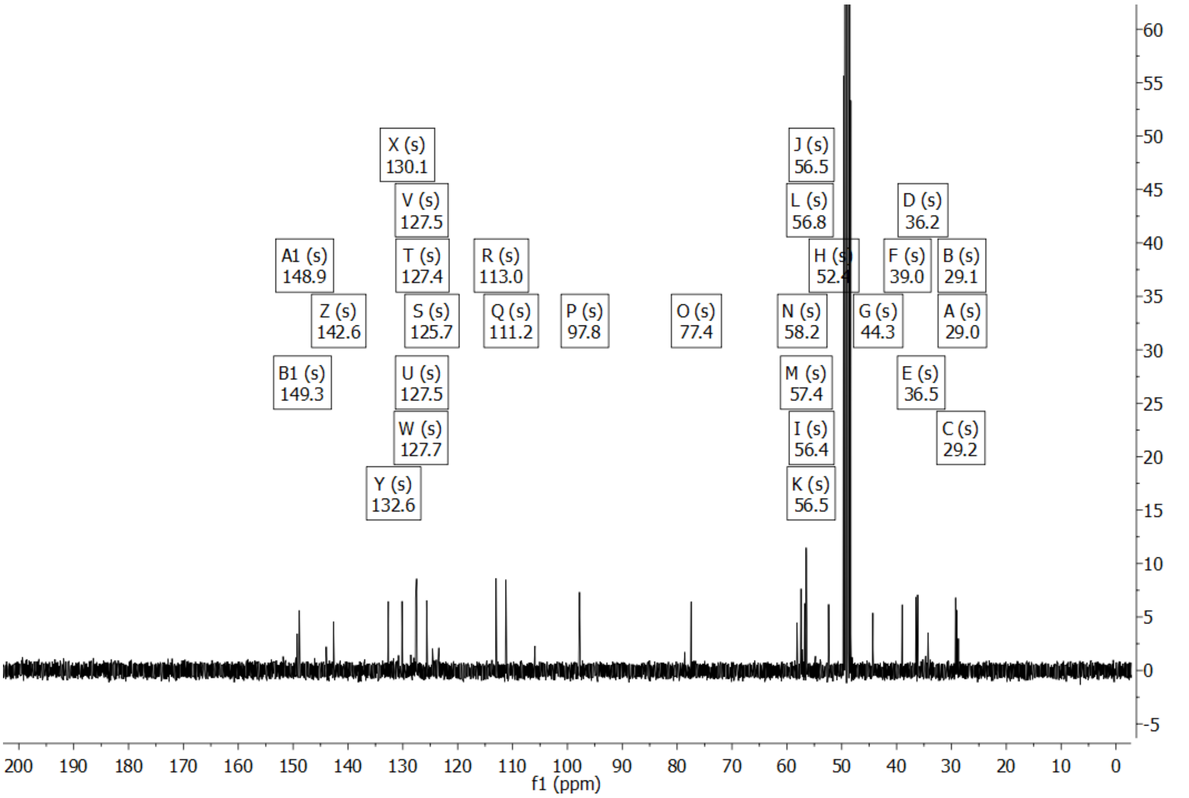

Supplement: Supplementary file 1 — Supplementary [file CMDC-16-1184-s001.pdf]
